# Lewis acid-catalyzed Pudovik reaction–phospha-Brook rearrangement sequence to access phosphoric esters

**DOI:** 10.3762/bjoc.18.123

**Published:** 2022-09-09

**Authors:** Jin Yang, Dang-Wei Qian, Shang-Dong Yang

**Affiliations:** 1 State Key Laboratory of Applied Organic Chemistry, Lanzhou University, Lanzhou 730000, P. R. China; 2 State Key Laboratory for Oxo Synthesis and Selective Oxidation, Lanzhou Institute of Chemical Physics, Lanzhou 730000, P. R. China

**Keywords:** Lewis acid, phospha-Brook rearrangement, phosphoric esters, Pudovik reaction

## Abstract

Herein, we report a Lewis acid-catalyzed Pudovik reaction–phospha-Brook rearrangement sequence between diarylphosphonates or -phosphinates and α-pyridinealdehydes to access valuable phosphoric ester compounds. This transformation provides an extended substrate scope that is complementary to similar previously reported base-catalyzed transformations.

## Introduction

**Scheme 1:**
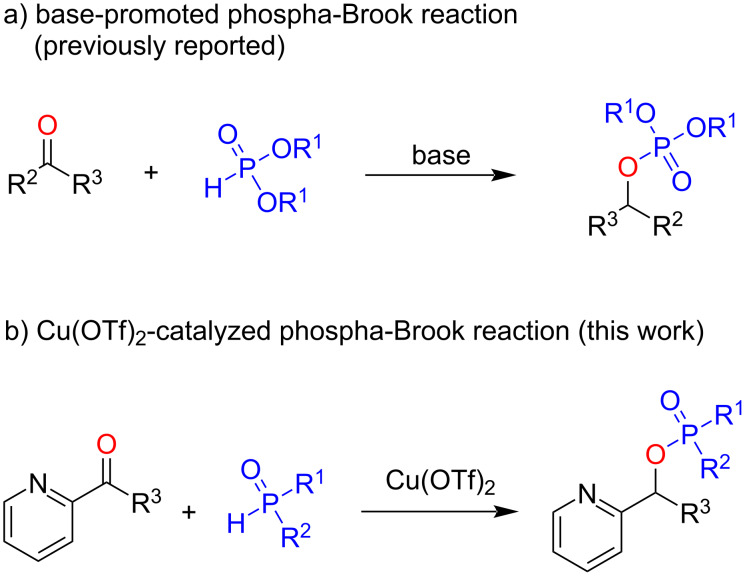
Different strategies for phospha-Brook reactions.

Phosphoric esters are widely used in agrochemistry, biological sciences, clinical treatments, as well as in general organic transformations [1–9]. Therefore, many efficient methods have been developed in the past decades to synthesize different types of phosphoric esters [10–18]. Traditional methods for the construction of P−O bonds in phosphoric esters rely on the phosphorylation of alcohols or phenol with highly air-sensitive and hazardous phosphorus halides, with the assistance of a suitable base [19–24]. As an alternative pathway, the phospha-Brook rearrangement [25–30] represents a green approach to phosphoric esters since it uses α-hydroxyphosphonates, which can be easily prepared by Pudovik reaction (addition of an unsaturated carbonyl compound to a labile P–H bond), to undergo an efficient intramolecular rearrangement, producing phosphoric esters [31–42]. For example, in 2005, Kaïm and co-workers have accomplished the synthesis of phosphoric esters through a 1,8-diazabicyclo[5.4.0]undec-7-ene (DBU)-catalyzed Pudovik reaction–phospha-Brook rearrangement sequence [43]. A decade later, Chakravarty and colleagues reported the efficient synthesis of organic phosphates from ketones and aldehydes using *n*-BuLi as catalyst through a similar transformation under solvent-free conditions [44]. Recently, Zhang’s group disclosed a cesium carbonate-catalyzed Pudovik reaction–phospha-Brook rearrangement sequence and extended the phosphorus source from phosphate to phosphonate [45]. Despite of these important advancements, all of the above transformations were carried out under basic conditions, and thus impose barriers for substrates that bear base-sensitive functional groups. More importantly, heteroatom-containing ketones and aldehydes have been proven to be challenging substrates for all of these existing systems [46–49]. Thus, searching for an alternative catalytic system, for example, a mild Lewis acid-catalyzed system, to achieve a wide applicability and provide a substrate scope complementary to previously reported base-catalyzed reactions, is a highly desirable task. However, such a process is recognized as challenging since there is no single report on such a sequence under Lewis acid catalysis. Herein, we report the synthesis of phosphoric esters by a Lewis acid Cu(OTf)_2_-catalyzed one-pot Pudovik reaction–phospha-Brook rearrangement sequence between pyridinyl-substituted aldehyde or pyridone with diarylphosphonates and -phosphinates (Scheme 1b). The present method is simple and efficient, providing an extended substrate scope that is complementary to classical similar base-prompted reactions.

## Results and Discussion

We used diphenylphosphine oxide (**1a**) and 2-pyridinecarboxaldehyde (**2a**) as the standard substrates to test the suitable conditions for the O-phosphination product **3aa**. Delightfully, in the presence of 10 mol % Cu(OTf)_2_ in toluene at 100 °C, the desired product **3aa** was obtained in 62% yield, and no Pudovik adduct **4aa** was detected (Table 1, entry 1). Inspired by this result, we conducted a careful screening of the other reaction factors to improve the reaction outcome. The screening of solvents was then carried out (Table 1, entries 1−5), and THF was found to be the optimal solvent. The temperature also played a pivotal role in the formation of **3aa**, with traces of, or no desired **3aa** being obtained at a temperature lower than 80 °C, but **4aa** was produced in a high yield (Table 1, entries 7−9). When the reaction was performed at 120 °C, the yield of **3aa** was slightly lower than that at 100 °C.

**Table 1:** Reaction optimization.^a^

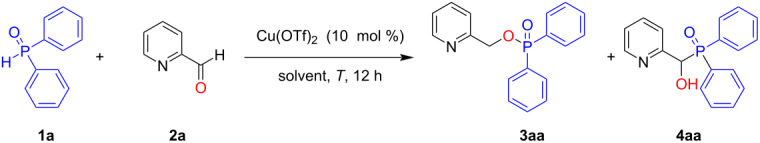				

entry	solvent	*T* (°C)	**3aa** (%)^b^	**4aa** (%)^b^

1	toluene	100	62	n. d.
2	MeCN	100	53	n. d.
3	DCM	100	53	n. d.
4	AcOEt	100	61	n. d.
5	THF	100	90	n. d.
6	THF	80	23	66
7	THF	60	traces	83
8	THF	40	n. d.	72
9	THF	25	n. d.	56
10	THF	120	74	n. d.

^a^Reaction conditions: diphenylphosphine oxide (**1a**, 0.2 mmol), 2-pyridinecarboxaldehyde (**2a**, 0.3 mmol), Cu(OTf)_2_ (10 mol %), solvent (2 mL), under Ar at 100 °C for 12 h. ^b^Isolated yield.

**Scheme 2:**
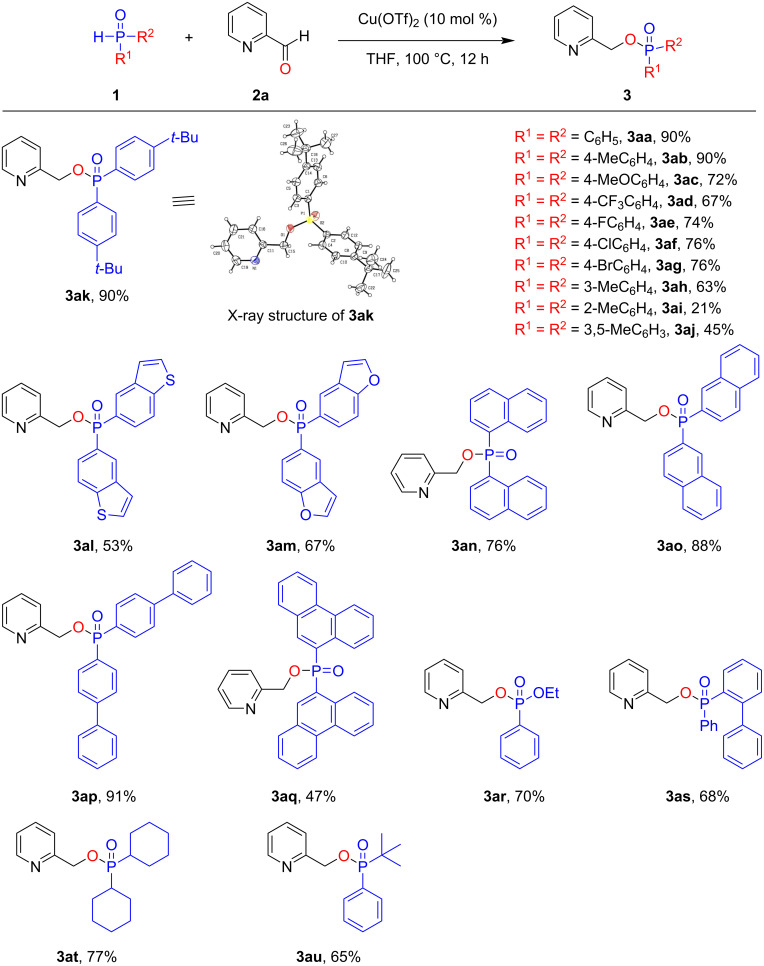
Scope of **1** (secondary phosphine oxides and phosphonate). Reaction conditions: **1** (0.2 mmol), 2-pyridinecarboxaldehyde (**2a**, 0.3 mmol), Cu(OTf)_2_ (10 mol %), THF (2 mL), under Ar at 100 °C for 12 h. The isolated yield is given.

With the high-yielding reaction conditions established (Table 1, entry 5), we examined a series of reactions of symmetric and asymmetric secondary phosphine oxides with 2-pyridinecarboxaldehyde (**2a**), which produced the corresponding O-phosphination products (Scheme 2). Diarylphosphine oxide substrates with either electron-withdrawing or electron-donating groups tethered to the phenyl ring were well tolerated, and the phosphinate products **3ab**–**ah** were obtained in moderate to good yield. The steric hindrance effect had a significant influence on the outcome of the reaction. For the phosphine oxide substrate **1i** bearing an *ortho*-methyl-substituted phenyl group, the desired product **3ai** was obtained in 21% yield, while the *meta*-methyl-substituted derivative **1h** was converted into the corresponding product **3ah** in 63% yield. In addition, the configuration of **3ak** was determined by an X-ray crystallographic analysis (CCDC 2177793). To our delight, phosphorus sources containing a heterocycle, such as a benzothiophene (in **1l**) or a benzofuran unit (in **1m**), could smoothly be transformed into the desired products **3al** and **3am**, respectively, in moderate yield. Phosphinates **3an** and **3ao** could both be prepared under this Pudovik reaction–phospha-Brook rearrangement sequence in moderate to good yield. When the phenyl groups of the diarylphosphine oxide **1a** were formally replaced by biphenyl units in **1p**, **3ap** was produced in 91% yield under the standard conditions. However, in the presence of bulky anthracene groups in **1q**, only a 47% yield of **3aq** was obtained. The applicability of the reaction system was further demonstrated with various unsymmetrically substituted phosphine oxides under the standard conditions. When the aryl group in the diarylphosphine oxide substrate was replaced by one or two alkyl groups or an ethoxy group, the transformation could also be achieved in moderate to good yield (see **3ar**–**au**).

**Scheme 3:**
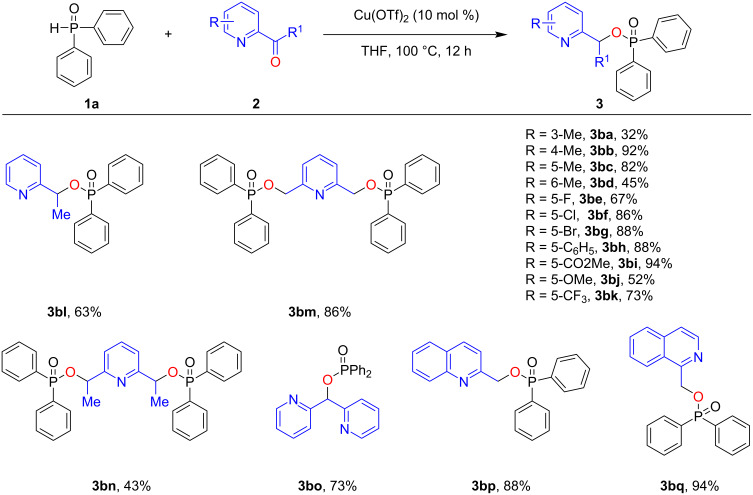
Scope of **2** (α-pyridinealdehydes and α-pyridones). Reaction conditions: diphenylphosphine oxide (**1a**, 0.2 mmol), **2** (0.3 mmol), Cu(OTf)_2_ (10 mol %), THF (2 mL), under Ar at 100 °C for 12 h. The isolated yield is given.

Next, we studied the scope with respect to the α-pyridinecarboxaldehyde by using **1a** as the reaction partner (Scheme 3). Firstly, we investigated the effect of steric hindrance on the pyridine ring of the α-pyridinealdehyde. Under standard conditions, a methyl group was introduced at either the 3-, 4-, or 5- position of the α-pyridinealdehyde, and the desired products **3ba**–**bc** were obtained in 32%, 92%, and 82%, respectively, indicating that the reaction is sensitive to steric effects. We then investigated the electronic effects of the α-pyridinealdehyde on the reaction outcome. However, no clear trend regarding electronic effects could be observed since α-pyridinealdehydes bearing either an electron-donating group (e.g., Me, MeO, Ph) or electron-withdrawing group (e.g., F, Cl, Br, CF_3_, CO_2_Me) in position 5 were all well tolerated, and the desired products were generally obtained in moderate to good yield. Delightfully, in addition to aldehydes, a ketone was also applicable under standard conditions, albeit affording the product in a comparably lower yield, probably due to the lower reactivity and steric hindrance of the substrate (see **3bl**). Moreover, pyridine bearing two formaldehyde or ketone groups could also be transformed into the desired diphosphination products **3bm** and **3bn** in moderate to good yield. The generality of the system was further showcased by tolerating quinoline and isoquinoline groups, and the desired products **3bp** and **3bq** were afforded in a high yield.

**Scheme 4:**
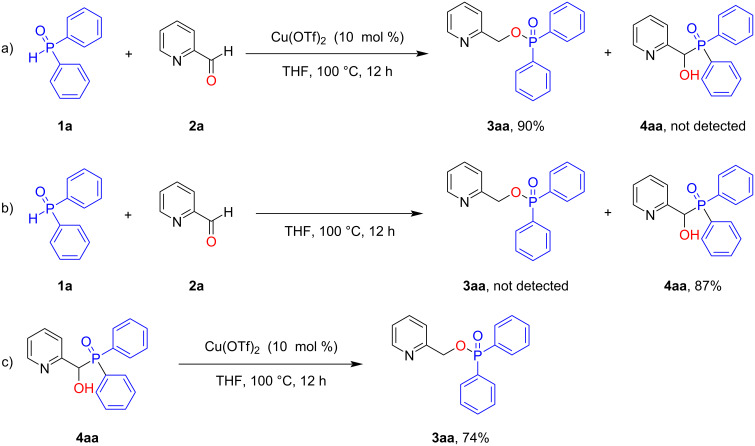
Control experiments.

Additional experiments were conducted in order to clarify the reaction mechanism. Under standard conditions, only pyridin-2-ylmethyl diphenylphosphinate (**3aa**) was produced, and the Pudovik adduct (hydroxy(pyridin-2-yl)methyl)diphenylphosphine oxide (**4aa**) was not detected (Scheme 4a). The control experiment showed that in the absence of Cu(OTf)_2_ catalyst, the reaction produced **4aa** as the sole product in 87% yield (Scheme 4b). When **4aa** was used as the substrate to carry out the phospha-Brook rearrangement under the standard conditions, phosphinate **3aa** was afforded in 74% yield (Scheme 4c). Taken together all of the above results, we concluded that **4aa** is the intermediate of this transformation and that Cu(OTf)_2_ promotes the phospha-Brook rearrangement occurring in the reaction.

**Scheme 5:**
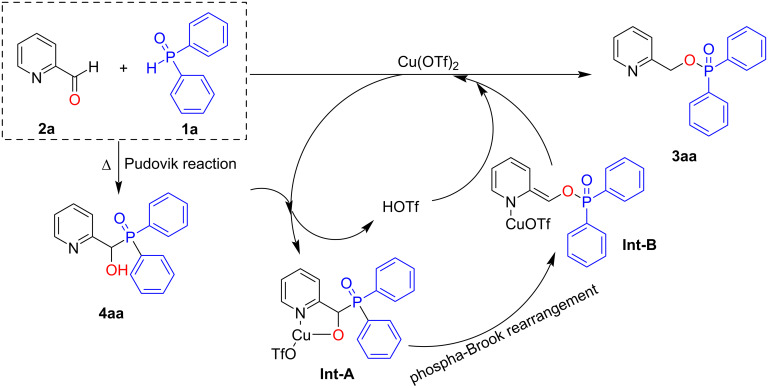
Proposed mechanism.

Based on the above results and literature reports, the proposed mechanism is shown in Scheme 5. First, diphenylphosphine oxide (**1a**) and 2-pyridinecarboxaldehyde (**2a**) undergo the Pudovik reaction to produce the intermediate adduct **4aa**. Then, Cu(OTf)_2_ coordinates with **4aa** to form the intermediate **Int-A**, which goes through the phospha-Brook rearrangement process to form **Int-B**. Finally, **Int-B** is transformed into the product **3aa** and releases Cu(OTf)_2_ to close the catalytic cycle.

## Conclusion

In conclusion, a Lewis acid-catalyzed Pudovik reaction–phospha-Brook rearrangement sequence between diarylphosphonates or -phosphinates and α-pyridinealdehydes was developed. This approach provides an efficient approach towards phosphoric esters and provides a scope complementary to previous similar base-catalyzed transformation.

## Supporting Information

File 1 Experimental details and characterization data (^1^H, ^13^C, and ^31^P NMR as well as chromatograms) of products.
