# Supplementary material for: Lewis acid-catalyzed Pudovik reaction–phospha-Brook rearrangement sequence to access phosphoric esters
Source: Beilstein J Org Chem. 2022 Sep 9;18:1188–94. doi: 10.3762/bjoc.18.123 (PMC9475178; doi:10.3762/bjoc.18.123)

BEILSTEIN JOURNAL OF ORGANIC CHEMISTRY

Supporting Information

for

Lewis acid-catalyzed Pudovik reaction–phospha-Brook rearrangement sequence to access phosphoric esters

Jin Yang, Dang-Wei Qian and Shang-Dong Yang

Beilstein J. Org. Chem. **2022,** *18,* 1188–1194. doi:10.3762/bjoc.18.123

Experimental details and characterization data (^{1}H, ^{13}C, and ^{31}P NMR as well as chromatograms) of products

License and Terms: This is a supporting information file under the terms of the Creative Commons Attribution License (https://creativecommons.org/licenses/by/4.0). Please note that the reuse, redistribution and reproduction in particular requires that the author(s) and source are credited and that individual graphics may be subject to special legal provisions.
The license is subject to the *Beilstein Journal of Organic Chemistry* terms and conditions: (https://www.beilstein-journals.org/bjoc/terms)

Table of Contents

1. General Information S3

2. General Procedure for the Synthesis of secondary phosphine oxides S3

3. General Procedure S5

4. Experimental procedure for gram-scale reaction S6

5. References S7

6. Characterization Data S7

7. X-ray crystallographic data for 3ak. S26

8. Scanned ^{1}H NMR, ^{13}C NMR, ^{31}P NMR and ^{19}F NMR Spectra of All products S28

1.General Information

^{1}H NMR, ^{13}C NMR, ^{31}P NMR and ^{19}F NMR spectra were recorded at room temperature using a Avance-400 instruments (^{1}H NMR at 400 MHz, ^{13}C NMR at 125 MHz, ^{31}P NMR at 121 MHz and ^{19}F-NMR at 282 MHz), NMR spectra of all products were reported in ppm with reference to solvent signals [^{1}H NMR: $CD(H)Cl_3$ (7.26 ppm), ^{13}C NMR: $CD(H)Cl_3$ (77.00 ppm)]. Signal patterns are indicated as s, singlet; d, doublet; dd, doublets of doublet; t, triplet, and m, multiplet. The mass spectrometry was performed in the positive electrospray ionization (ESI+) mode. Reactions were monitored by thin-layer chromatography Column chromatography (petroleum ether/ethyl acetate) was performed on silica gel (200-300 mesh). Analytical grade solvents and commercially available reagents were purchased from commercial sources and used directly without further purification unless otherwise stated. Anhydrous tetrahydrofuran (THF) was prepared by refluxing the analyzed tetrahydrofuran with sodium.

2. General Procedure for the Synthesis of secondary phosphine oxides

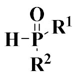

1a. $R^1=R^2= C_6H_5$
1b. $R^1=R^2 = 4\text{-}MeC_6H_4$
1c. $R^1=R^2= 4\text{-}MeOC_6H_4$
1d. $R^1=R^2 = 4\text{-}CF_3C_6H_4$
1e. $R^1=R^2 = 4\text{-}FC_6H_4$
1f. $R^1=R^2 = 4\text{-}ClC_6H_4$
1g. $R^1=R^2 = 4\text{-}BrC_6H_4$,
1h. $R^1=R^2 = 3\text{-}MeC_6H_4$
1i. $R^1=R^2 = 2\text{-}MeC_6H_4$
1j. $R^1=R^2 = 3,5\text{-}MeC_6H_3$
1k. $R^1=R^2 = 4\text{-}{}^tBuC_6H_4$

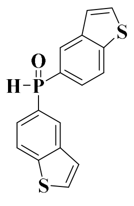

1l

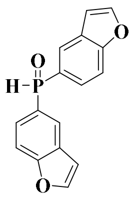

1m

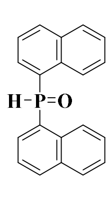

1n

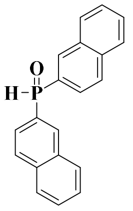

1o

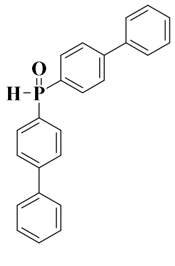

1p

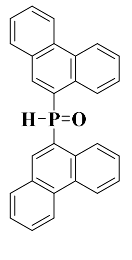

1q

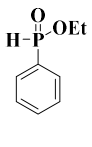

1r

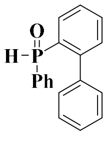

1s

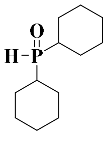

1t

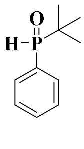

1u

General procedure A: Synthesis of symmetric secondary phosphine oxides

Synthesis of **1b** is representative

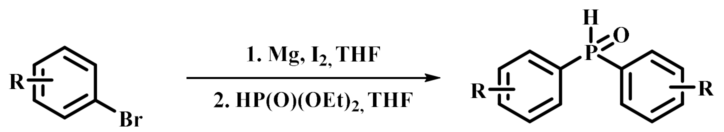

To a mixture of magnesium (0.53 g, 22 mmol) and THF (10 mL) was added a solution of 1-bromo-4-methylbenzene (2.46 mL, 20 mmol) in THF (10 mL) dropwise with a dropping funnel at 0 °C and the resulting mixture was stirred for 1 h. After cooled to 0 °C, a solution of diethylphosphite (0.65 mL, 5.0 mmol) in THF (10 mL) was added dropwise with a dropping funnel at 0 °C. After stirring at 0 °C for 1 h, the reaction was quenched with 100 ml 0.1 N HCl and the product was extracted with AcOEt. The combined organic layer was washed with brine, dried over Na_2SO4 and concentrated under reduced pressure. The residue was purified by silica gel column chromatography (petroleum ether/ethyl acetate= 1:1) followed by recrystallization from a petroleum ether/ethyl acetate mixture to afford **1b**.

The spectral data were in accordance with those previously reported in the literature.[1]

General procedure B: Synthesis of asymmetric secondary phosphine oxides

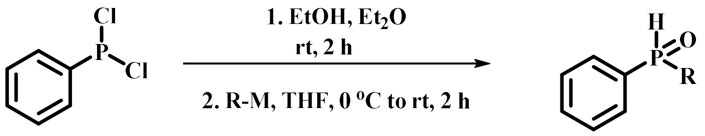

A 50 mL round-bottomed flask equipped with a magnetic stirrer under air atmosphere was charged with dichlorophenylphosphine (1.35 mL, 10.0 mmol) and Et_2O (25 mL). EtOH (1.45 mL, 25.0 mmol) was added dropwise over 5 mins and the resulting solution was stirred at r.t for 2 hours. The solvent was removed under vacuum and the resulting crude ethyl phosphinate was dissolved in THF (12 mL) under a nitrogen atmosphere. The appropriate organometallic reagent (2.2 equiv.) in THF was cooled to

0 °C under an inert atmosphere and the ethyl phosphinate solution was added dropwise at 0 °C over 30 mins. The reaction was stirred at r.t for 2 hours then quenched with sat. aq. NH_4Cl solution. Water (70 mL) was then added and the aqueous phase was then extracted with $CHCl_2$ (3 × 150 mL). The combined organic fractions were dried over anhydrous Na_2SO_4, concentrated in vacuo, and the crude residue purified by column chromatography to afford the desired secondary phosphine oxide.

The spectral data were in accordance with those previously reported in the literature.[2,3,4]

3.General Procedure

3.1 Table S1. Screening of Optimal Reaction Conditions

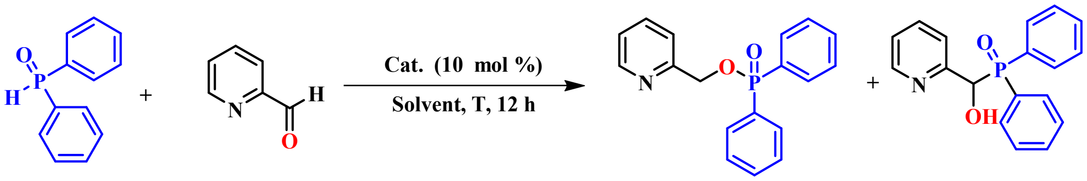

1a 2a 3aa 4aa

entry	cat.	Solvent	Temp/°C	3aa(%)[b]	3aa(%)[b]
1	$Cu(OTf)_2$	THF	100	90	N.D.
2	$Zn(OTf)_2$	THF	100	80	trace
3	$Al(OTf)_3$	THF	100	83	trace
4	$Bi(OTf)_3$	THF	100	86	N.D.
5	AgOTf	THF	100	68	trace
6	$Sn(OTf)_2$	THF	100	71	trace
7	$Sc(OTf)_3$	THF	100	74	trace
8	$Cu(OTf)_2$	MeCN	100	53	N.D.
9	$Cu(OTf)_2$	DCM	100	53	N.D.
10	$Cu(OTf)_2$	AcOEt	100	61	N.D.
11	$Cu(OTf)_2$	Tol	100	62	N.D.
12	$Cu(OTf)_2$	THF	80	23	66
13	$Cu(OTf)_2$	THF	60	trace	83
14	$Cu(OTf)_2$	THF	40	N.D.	72
15	$Cu(OTf)_2$	THF	25	N.D.	56
16	$Cu(OTf)_2$	THF	120	74	N.D.

Table S1[a]Reaction conditions:diphenylphosphine oxide (0.2 mmol),2-Pyridinecarboxaldehyde (0.3 mmol), catalyst (10 mol%), solvent (2 mL) underArat 100 °C for 12 h.[b]Isolated yields unlessotherwise noted.

3.2General Procedure for the Synthesis of O-Phosphination

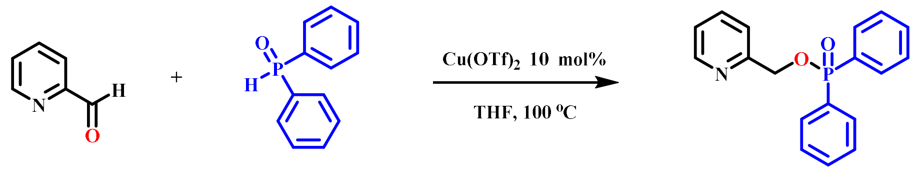

To a 10 mL glass tube equipped with a stir bar was added picolinaldehyde (0.3 mmol, 1.5equiv, 28.5 ul), diphenylphosphine oxide (0.2 mmol, 1.0equiv, 40.0 mg), $Cu(OTf)_2$ (0.01 mmol, 10 mol%, 7.2 mg). The glass tube is sealed with a rubber stopper and the air in the tube is replaced with argon three times, then the anhydrous THF (2 ml) was added to the mixed solution by microinjector and stirred at 100 °C for 12 h until complete consumption of starting material diphenylphosphine oxide as monitored by TLC analysis. After the reaction was finished, the resulting solution was filtered and washed with ethyl acetate. The combined filtrates were concentrated under reduced pressure to give the crude product The residue was purified on a silica-gel column chromatography (eluent: petroleum ether/ethyl acetate. Df= 1 : 2) to provide the desired product (90%, 55.6 mg).

4. Experimental procedure for gram-scale reaction

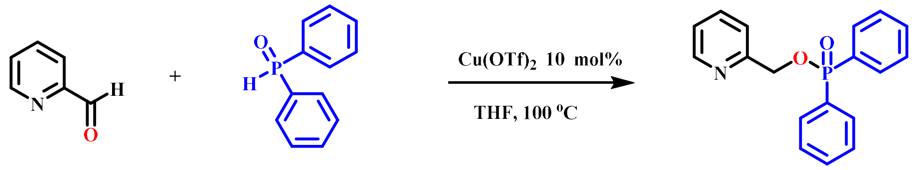

To a 100 mL glass tube equipped with a stir bar was added picolinaldehyde (15 mmol, 1.5equiv, 1.43 ml), diphenylphosphine oxide (10 mmol, 1.0equiv, 2.02 g), $Cu(OTf)_2$ (0.01 mmol, 10 mol%, 0.362 g). The glass tube is sealed with a rubber stopper and the air in the tube is replaced with argon three times, then the anhydrous

THF (40 ml) was added to the mixed solution by microinjector and stirred at 100 °C for 12 h until complete consumption of starting material diphenylphosphine oxide as monitored by TLC analysis. After the reaction was finished, the resulting solution was filtered and washed with ethyl acetate. The combined filtrates were concentrated under reduced pressure to give the crude product The residue was purified on a silica-gel column chromatography (eluent: petroleum ether/ethyl acetate. Df= 1 : 2) to provide the desired product .(83%, 2.56g).

5. References

1.Zhang, X.; Wang, J.; Yang,S.D. ACS Catal. 2021, 11, 14008-14015.

2.Kuo,L. Y.; Baker,D. C.;Dortignacq,A. K.; Dill,K. M. Organometallics. 2013, 32, 4759-4765.

3. Beaud,R.; Phipps,R. J.; Gaunt, M. J. J. Am. Chem. Soc. 2016, 138, 13183-13186.

4. Petit,C.; Favre-Réguillon,A.;Mignani,G.; Lemaire,M. Green Chem. 2010, 12, 326-330.

6. Characterization Data

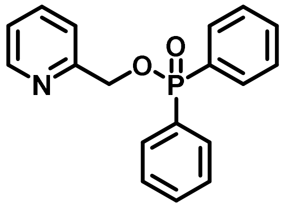

pyridin-2-ylmethyl diphenylphosphinate (3aa)

Compound 3aa was isolated in 90% yield;**^{1}H NMR** (400 MHz, $CDCl_3$) δ 8.52 (d, *J* = 4.6 Hz, 1H), 7.94 – 7.78 (m, 4H), 7.74 – 7.62 (m, 1H), 7.60 – 7.34 (m, 7H), 7.17 (dd, *J* = 7.0, 5.3 Hz, 1H), 5.19 (d, *J* = 7.4 Hz, 2H).**^{13}C NMR** (101 MHz, $CDCl_3$) δ 156.18 (d, *J* = 8.1 Hz), 149.06 (s), 136.84 (s), 132.34 (d, *J* = 2.7 Hz), 131.60 (d, *J* = 10.2 Hz), 130.21 (s), 128.64 (t, *J* = 11.8 Hz), 122.84 (s), 121.49 (s), 66.76 (d, *J* = 5.4 Hz). **^{31}P NMR** (162 MHz, $CDCl_3$) δ 32.93 (s).**HRMS (ESI):** $[M+H]^+$calcd for $C_{18}H_{16}NO_2P$310.0991, found 310.0988.

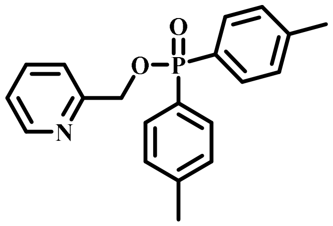

pyridin-2-ylmethyl di-p-tolylphosphinate (3ab)

Compound 3ab was isolated in 90% yield; ^{1}**H NMR** (400 MHz, $CDCl_3$) δ 8.53 (d, *J* = 4.6 Hz, 1H), 7.80 – 7.64 (m, 5H), 7.54 (t, *J* = 10.0 Hz, 1H), 7.26 (dd, *J* = 7.9, 3.0 Hz, 4H), 7.24 – 7.17 (m, 1H), 5.14 (d, *J* = 7.3 Hz, 2H), 2.38 (s, 7H).^{13}**C NMR** (101 MHz, $CDCl_3$) δ 156.67 (d, *J* = 8.4 Hz), 149.12 (s), 142.84 (d, *J* = 2.8 Hz), 136.86 (s), 131.72 (d, *J* = 10.6 Hz), 129.36 (d, *J* = 13.6 Hz), 128.62 (s), 127.23 (s), 122.78 (s), 121.50 (s), 66.68 (d, *J* = 5.3 Hz), 21.66 (s).^{31}**P NMR** (162 MHz, $CDCl_3$) δ 33.98 (s).**HRMS (ESI):** $[M+H]^+$calcd for $C_{20}H_{20}NO_2P$338.1304, found 338.1306.

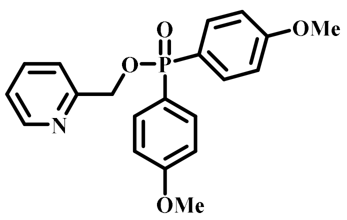

pyridin-2-ylmethyl bis(4-methoxyphenyl)phosphinate(3ac)

Compound 3ac was isolated in 72% yield; ^{1}**H NMR** (400 MHz, $CDCl_3$) δ 8.54 (d, *J* = 4.1 Hz, 1H), 7.79 (dd, *J* = 11.5, 8.7 Hz,4H), 7.70 (t, *J* = 7.7 Hz, 1H), 7.56 (t, *J* = 7.0 Hz, 1H), 7.19 (dd, *J* = 17.6, 10.3 Hz, 1H), 6.96 (dd, *J* = 8.5, 2.1 Hz, 4H), 5.13 (d, *J* = 7.3 Hz, 2H),δ 3.83 (d, *J* = 2.1 Hz, 6H).^{13}**C NMR** (101 MHz, $CDCl_3$) δ 162.68 (d, *J* = 3.0 Hz), 156.70 (d, *J* = 8.3 Hz), 149.12 (s), 136.84 (s), 133.71 (dd, *J* = 27.9, 11.4 Hz), 123.33 (s), 122.76 (s), 121.90 (s), 121.49 (s), 114.05 (t, *J* = 15.9 Hz), 66.59 (d, *J* = 5.2 Hz), 55.33 (s).^{31}**P NMR** (162 MHz, $CDCl_3$) δ 33.82 (s).**HRMS (ESI):** $[M+H]^+$calcd for $C_{20}H_{20}NO_4P$370.1203, found 370.1204.

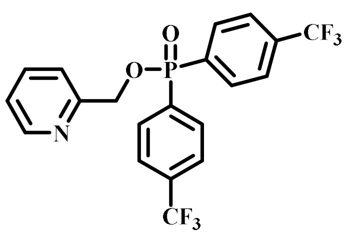

pyridin-2-ylmethyl bis(4-(trifluoromethyl)phenyl)phosphinate (3ad)

Compound 3ad was isolated in 67% yield;^{1}**H NMR** (400 MHz, $CDCl_3$) δ 8.57 (d, *J* = 4.3 Hz, 1H), 8.02 (dd, *J* = 12.1, 8.0 Hz, 2H), 7.81 – 7.67 (m, 2H), 7.51 (t, *J* = 11.5 Hz, 1H), 7.26 (dd, *J* = 7.0, 5.1 Hz, 1H), 5.22 (d, *J* = 8.0 Hz, 1H).^{13}**C NMR** (101 MHz, $CDCl_3$) δ 155.32 (d, *J* = 7.6 Hz), 149.49 (s), 136.96 (s), 134.45 (ddd, *J* = 53.5, 32.8, 25.7 Hz), 132.26 (d, *J* = 10.6 Hz), 125.67 (dq, *J* = 13.4, 3.7 Hz), 124.73 (s), 123.29 (s), 121.99 (s), 65.15 (t, *J* = 362.7 Hz). ^{19}**F NMR** (376 MHz, $CDCl_3$) δ -59.38 – -73.33 (m).^{31}**P NMR** (162 MHz, $CDCl_3$) δ 29.04 (s).**HRMS (ESI):** $[M+H]^+$calcd for $C_{20}H_{14}F_6NO_2P$ 446.0739, found 446.0740.

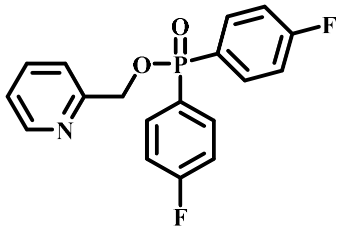

pyridin-2-ylmethyl bis(4-fluorophenyl)phosphinate (3ae)

Compound 3ae was isolated in 74% yield; ^{1}**H NMR** (400 MHz, $CDCl_3$) δ 8.56 (d, *J* = 4.5 Hz, 1H), 7.86 (ddd, *J* = 12.0, 8.5, 5.7 Hz, 4H), 7.71 (t, *J* = 7.7 Hz, 1H), 7.51 (t, *J* = 9.6 Hz, 1H), 7.26 – 7.21 (m, 1H), 7.16 (td, *J* = 8.6, 2.3 Hz, 4H), 5.15 (d, *J* = 7.7 Hz, 2H).^{13}**C NMR** (101 MHz, $CDCl_3$) δ 166.62 (d, *J* = 3.6 Hz), 164.09 (d, *J* = 3.5 Hz), 155.95 (d, *J* = 8.0 Hz), 149.36 (s), 136.93 (s), 134.29 (dd, *J* = 11.7, 8.9 Hz), 127.60 (d, *J* = 3.5 Hz), 126.19 (d, *J* = 3.3 Hz), 123.07 (s), 121.77 (s), 116.14 (dd, *J* = 21.5, 14.5 Hz), 67.05 (d, *J* = 5.4 Hz).^{19}**F NMR** (376 MHz, $CDCl_3$) δ -105.54 (d, *J* = 1.0 Hz). ^{31}**P** NMR (162 MHz, CDCl3) δ 31.14 (s).**HRMS (ESI):** $[M+H]^+$calcd for $C_{18}H_{14}F_2NO_2P$346.0803, found 346.0796.

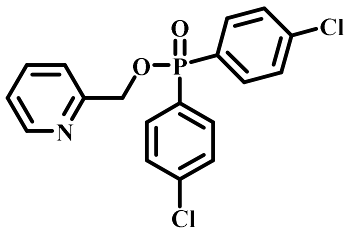

pyridin-2-ylmethyl bis(4-chlorophenyl)phosphinate (3af)

Compound 3af was isolated in 76% yield;**^{1}H NMR** (400 MHz, $CDCl_3$) δ 8.56 (d, *J* = 4.5 Hz, 1H), 7.78 (dd, *J* = 11.9, 8.3 Hz, 4H), 7.71 (t, *J* = 7.7 Hz, 1H), 7.51 – 7.41 (m, 5H), 7.26 – 7.20 (m, 1H), 5.16 (d, *J* = 7.7 Hz, 2H).**^{13}C NMR** (101 MHz, $CDCl_3$) δ 155.78 (d, *J* = 7.9 Hz), 149.39 (s), 139.24 (d, *J* = 3.6 Hz), 136.95 (s), 133.10 (d, *J* = 11.1 Hz), 129.88 (s), 129.14 (d, *J* = 13.9 Hz), 128.49 (s), 123.12 (s), 121.81 (s), 67.16 (d, *J* = 5.4 Hz). 31P NMR (162 MHz, CDCl3) δ 31.01(s).**HRMS (ESI):** [M+H]$^{+}$calcd for $C_{18}H_{14}Cl_2NO_2P$378.0212, found 378.0209.

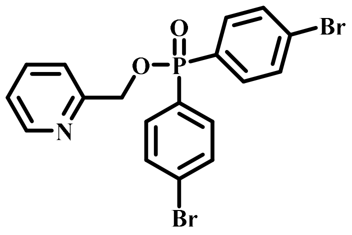

pyridin-2-ylmethyl bis(4-bromophenyl)phosphinate (3ag)

Compound 3ag was isolated in 76% yield; **^{1}H NMR** (400 MHz, $CDCl_3$) δ 8.56 (d, *J* = 4.5 Hz, 1H), 7.71 (dd, *J* = 12.0, 8.4 Hz, 5H), 7.61 (dd, *J* = 8.4, 3.0 Hz, 4H), 7.48 (d, *J* = 7.8 Hz, 1H), 7.24 (dd, *J* = 7.2, 5.1 Hz, 1H), 5.16 (d, *J* = 7.7 Hz, 2H).**^{13}C NMR** (101 MHz, $CDCl_3$) δ 155.74 (d, *J* = 8.0 Hz), 149.39 (s), 136.98 (s), 133.19 (d, *J* = 11.1 Hz), 132.10 (d, *J* = 13.8 Hz), 130.33 (s), 128.95 (s), 127.91 (d, *J* = 3.7 Hz), 123.15 (s), 121.84 (s), 67.17 (d, *J* = 5.4 Hz).**^{31}P NMR** (162 MHz, $CDCl_3$) δ 31.27 (s).**HRMS (ESI):** [M+H] $^{+}$calcd for $C_{18}H_{14}Br_2NO_2P$ 465.9202, found 465.9213.

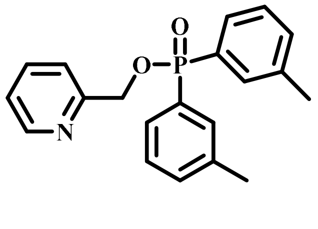

pyridin-2-ylmethyl di-m-tolylphosphinate (3ah)

Compound 3ah was isolated in 63% yield;**^{1}H NMR** (400 MHz, $CDCl_3$) δ 8.54 (s, 1H), 7.73 – 7.61 (m, 5H), 7.56 (d, *J* = 7.8 Hz, 1H), 7.36 – 7.31 (m, 4H), 7.21 (dd, *J* = 6.9, 5.1 Hz, 1H), 5.16 (d, *J* = 7.6 Hz, 2H), 2.37 (s, 6H). **^{13}C NMR** (101 MHz, $CDCl_3$) δ 156.53 (d, *J* = 8.0 Hz), 149.07 (s), 138.48 (d, *J* = 13.2 Hz), 136.88 (s), 133.14 (d, *J* = 2.9 Hz), 132.20 (d, *J* = 10.3 Hz), 131.59 (s), 130.23 (s), 128.63 (dd, *J* = 22.1, 12.0 Hz), 122.85 (s), 121.69 (s), 66.82 (d, *J* = 5.4 Hz), 21.37 (s).**^{31}P NMR** (162 MHz, $CDCl_3$) δ 35.90 (s).**HRMS (ESI):** [M+H] $^+$calcd for $C_{20}H_{20}NO_2P$338.1304, found 338.1304.

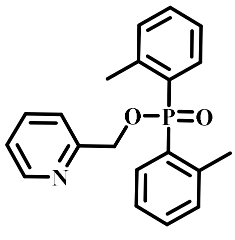

pyridin-2-ylmethyl di-o-tolylphosphinate(3ai)

Compound 3ai was isolated in 21% yield; **^{1}H NMR** (400 MHz, $CDCl_3$) δ 8.55 (d, *J* = 4.7 Hz, 1H), 8.05 – 7.90 (m, 2H), 7.71 (td, *J* = 7.7, 1.7 Hz, 1H), 7.56 (d, *J* = 7.8 Hz, 1H), 7.49 – 7.37 (m, 2H), 7.30 (td, *J* = 7.5, 2.6 Hz, 2H), 7.21 (dd, *J* = 13.0, 8.0 Hz, 3H), 5.15 (d, *J* = 7.3 Hz, 2H), 2.36 (s, 6H).**^{13}C NMR** (101 MHz, $CDCl_3$) δ 156.54 (d, *J* = 8.2 Hz), 149.22 (s), 141.74 (d, *J* = 11.3 Hz), 136.86 (s), 133.61 (d, *J* = 9.9 Hz), 132.45 (d, *J* = 2.7 Hz), 131.50 (d, *J* = 12.7 Hz), 130.19 (s), 128.88 (s), 125.64 (d, *J* = 12.7 Hz), 122.85 (s), 121.83 (s), 66.57 (d, *J* = 5.1 Hz), 21.25 (d, *J* = 4.3 Hz). 31P NMR (121 MHz, CDCl3) δ 34.1 (s).**HRMS (ESI):** [M+H] $^+$calcd for $C_{20}H_{20}NO_2P$338.1304, found 338.1301.

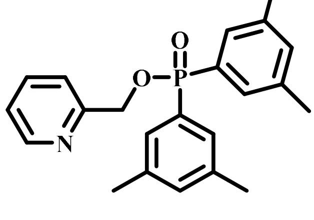

pyridin-2-ylmethyl bis(3,5-dimethylphenyl)phosphinate (3aj)

Compound 3aj was isolated in 45% yield;**^{1}H NMR** (400 MHz, $CDCl_3$) δ 8.69 – 8.39 (m, 1H), 7.72 (td, *J* = 7.7, 1.7 Hz, 1H), 7.58 (d, *J* = 7.8 Hz, 1H), 7.50 (d, *J* = 12.6 Hz, 4H), 7.25 – 7.20 (m, 1H), 7.16 (s, 2H), 5.17 (d, *J* = 7.7 Hz, 2H), 2.34 (s, 12H).**^{13}C NMR** (101 MHz, $CDCl_3$) δ 156.69 (d, *J* = 7.8 Hz), 149.08 (s), 138.30 (d, *J* = 13.9 Hz), 136.82 (s), 134.06 (d, *J* = 3.0 Hz), 131.50 (s), 130.15 (s), 129.25 (d, *J* = 10.2 Hz), 122.78 (s), 121.66 (s), 66.80 (d, *J* = 5.4 Hz), 21.28 (s).**^{31}P NMR** (162 MHz, $CDCl_3$) δ 34.38 (s).**HRMS (ESI):** $[M+H]^+$calcd for $C_{22}H_{24}NO_2P$366.1617, found 366.1612.

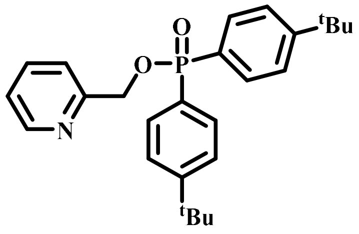

pyridin-2-ylmethyl bis(4-(tert-butyl)phenyl)phosphinate (3ak)

Compound 3ak was isolated in 90% yield;**^{1}H NMR** (400 MHz, $CDCl_3$) δ 8.53 (d, *J* = 4.7 Hz, 1H), 7.80 (dd, *J* = 12.0, 8.2 Hz, 4H), 7.74 – 7.67 (m, 1H), 7.57 (d, *J* = 7.8 Hz, 1H), 7.47 (dd, *J* = 8.3, 3.2 Hz, 4H), 7.24 – 7.17 (m, 1H), 5.14 (d, *J* = 7.3 Hz, 2H), 1.31 (s, 18H).**^{13}C NMR** (101 MHz, DMSO) δ 152.02 (d, *J* = 8.4 Hz), 150.98 (d, *J* = 2.9 Hz), 144.34 (s), 132.08 (s), 126.87 (d, *J* = 10.6 Hz), 123.91 (s), 122.52 (s), 120.86 (d, *J* = 13.4 Hz), 117.98 (s), 116.77 (s), 61.95 (d, *J* = 5.3 Hz), 30.28 (s), 26.34 (s). **^{31}P NMR** (162 MHz, $CDCl_3$) δ 33.77 (s). **HRMS (ESI):** $[M+H]^+$calcd for $C_{26}H_{32}NO_2P$ 422.2243, found 422.2242.

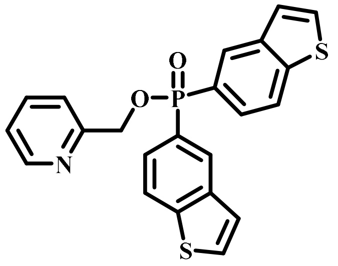

pyridin-2-ylmethyl bis(benzo[b]thiophen-5-yl)phosphinate (3al)

Compound 3al was isolated in 53% yield;^{1}**H NMR** (400 MHz, $CDCl_3$) δ 8.58 – 8.50 (m, 1H), 8.43 (d, *J* = 13.4 Hz, 2H), 7.95 (dd, *J* = 8.3, 2.8 Hz, 2H), 7.79 (dd, *J* = 10.3, 9.2 Hz, 2H), 7.75 – 7.65 (m, 1H), 7.58 (d, *J* = 7.8 Hz, 1H), 7.50 (dd, *J* = 5.3, 3.7 Hz, 2H), 7.37 (dt, *J* = 12.0, 5.9 Hz, 2H), 7.24 – 7.14 (m, 1H), 5.23 (d, *J* = 7.5 Hz, 2H).^{13}**C NMR** (101 MHz, $CDCl_3$) δ 156.44 (d, *J* = 8.1 Hz), 149.23 (s), 143.64 (d, *J* = 2.9 Hz), 139.32 (d, *J* = 15.1 Hz), 136.89 (s), 128.23 – 127.63 (m), 127.55 (s), 126.38 – 125.71 (m), 124.20 (s), 122.98 (d, *J* = 14.7 Hz), 121.73 (s), 67.04 (d, *J* = 5.4 Hz).^{31}**P NMR** (162 MHz, $CDCl_3$) δ 34.58 (s).**HRMS (ESI):** [M+H] $^{+}$calcd for $C_{22}H_{16}NO_2PS_2$ 422.0433, found 422.0433.

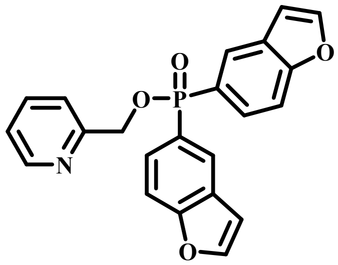

pyridin-2-ylmethyl di(benzofuran-5-yl)phosphinate (3am)

Compound 3am was isolated in 67% yield;^{1}**H NMR** (400 MHz, $CDCl_3$) δ 8.64 – 8.51 (m, 1H), 8.24 (dd, *J* = 12.8, 0.9 Hz, 2H), 7.84 (ddd, *J* = 11.6, 8.5, 1.4 Hz, 2H), 7.77 – 7.67 (m, 3H), 7.65 – 7.55 (m, 3H), 7.23 (dd, *J* = 7.0, 5.4 Hz, 1H), 6.91 – 6.79 (m, 2H), 5.22 (d, *J* = 7.4 Hz, 2H).^{13}**C NMR** (101 MHz, $CDCl_3$) δ 156.93 (d, *J* = 3.0 Hz), 156.54 (d, *J* = 8.2 Hz), 149.20 (s), 146.29 (s), 136.88 (s), 124.87 (s), 122.87 (s), 121.67 (s), 106.88 (s), 66.91 (d, *J* = 5.3 Hz).^{31}**P NMR** (162 MHz, $CDCl_3$) δ 34.81 (s).**HRMS (ESI):** [M+H] $^{+}$calcd for $C_{22}H_{16}NO_4P$ 390.0890, found 390.0888.

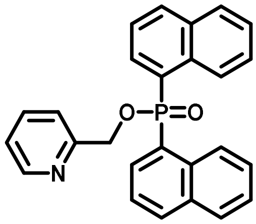

pyridin-2-ylmethyl di(naphthalen-1-yl)phosphinate(3an)

Compound 3an was isolated in 76% yield; **^{1}H NMR** (400 MHz, CDCl3) δ 8.68 – 8.59 (m, 2H), 8.45 (dd, J = 15.9, 4.8 Hz, 1H), 8.21 (ddd, J = 15.9, 7.1, 1.1 Hz, 2H), 8.01 (d, J = 8.2 Hz,2H), 7.84 (dd, J = 15.6, 8.4 Hz, 2H), 7.60 (t, J = 7.7 Hz, 1H), 7.47 (ddd, J = 14.8, 13.5, 7.4 Hz, 7H), 7.12 (dd, J = 12.7, 6.5 Hz, 1H), 5.27 (d, J = 7.4 Hz, 2H). **^{13}C NMR** (101 MHz, CDCl3) δ 156.38 (d, J = 8.4 Hz), 149.15 (s), 136.76 (s), 134.16 (d, J = 10.4 Hz), 133.88 – 133.49 (m), 133.00 (d, J = 10.5 Hz), 128.95 (d, J = 1.5 Hz), 128.11 (s), 127.55 (s), 126.79 (s), 126.55 (d, J = 4.9 Hz), 126.43 (s), 124.72 (d, J = 15.0 Hz), 122.80 (s), 121.71 (s), 67.13 (d, J = 5.3 Hz). **^{31}P NMR** (162 MHz, CDCl3) δ 35.72 (s).**HRMS (ESI):** [M+H] $^+$calcd for $C_{26}H_{20}NO_2P$ 410.1304, found 410.1306.

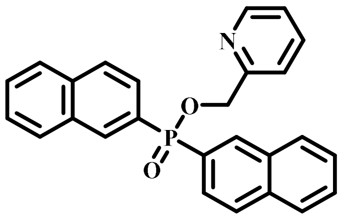

pyridin-2-ylmethyl di(naphthalen-2-yl)phosphinate (3ao)

Compound 3ao was isolated in 88% yield; **^{1}H NMR** (400 MHz, $CDCl_3$) δ 8.56 (t, J = 9.9 Hz, 3H), 7.96 – 7.78 (m, 8H), 7.69 (td, J = 7.7, 1.7 Hz, 1H), 7.64 – 7.48 (m, 5H), 7.19 (dd, J = 6.7, 5.3 Hz, 1H), 5.27 (d, J = 7.6 Hz, 2H).**^{13}C NMR** (101 MHz, $CDCl_3$) δ 156.41 (d, J = 8.0 Hz), 149.26 (s), 136.92 (s), 134.96 (d, J = 2.4 Hz), 134.04 (d, J = 10.0 Hz), 132.48 (d, J = 14.5 Hz), 129.05 (s), 128.82 (s), 128.70 (s), 128.57 (s), 128.41 (s), 127.86 (s), 127.45 (s), 127.00 (s), 126.34 (d, J = 10.8 Hz), 122.94 (s), 121.75 (s), 67.13 (d, J = 5.4 Hz).**^{31}P NMR** (162 MHz, $CDCl_3$) δ 33.39 (s).**HRMS (ESI):** [M+H] $^+$calcd for $C_{26}H_{20}NO_2P$ 410.1304, found 410.1300.

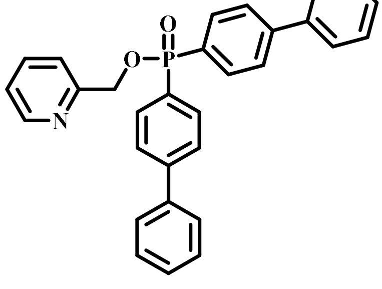

pyridin-2-ylmethyl di([1,1'-biphenyl]-4-yl)phosphinate (3ap)

Compound 3ap was isolated in 91% yield;**^{1}H NMR** (400 MHz, $CDCl_3$) δ 8.34 (d, *J* = 4.6 Hz, 1H), 7.76 (dd, *J* = 12.0, 8.3 Hz, 4H), 7.50 (ddd, *J* = 11.3, 8.0, 2.4 Hz, 5H), 7.41 – 7.34 (m, 5H), 7.24 (t, *J* = 7.5 Hz, 4H), 7.17 (t, *J* = 7.3 Hz, 2H), 7.03 – 6.97 (m, 1H), 5.01 (d, *J* = 7.5 Hz, 2H).**^{13}C NMR** (101 MHz, $CDCl_3$) δ 156.47 (d, *J* = 8.1 Hz), 149.26 (s), 145.22 (d, *J* = 2.9 Hz), 139.93 (s), 136.91 (s), 132.28 (d, *J* = 10.5 Hz), 130.34 (s), 128.98 (s), 128.22 (s), 127.78 – 126.69 (m), 122.92 (s), 121.71 (s), 67.00 (d, *J* = 5.4 Hz).**^{31}P NMR** (162 MHz, $CDCl_3$) δ 33.07 (s).**HRMS (ESI):** [M+H] +calcd for $C_{30}H_{24}NO_2P$ 462.1617, found 462.1622.

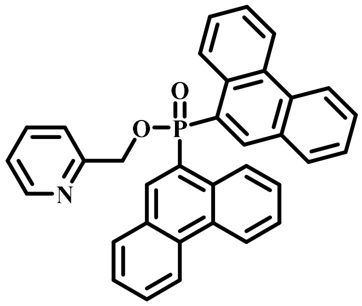

pyridin-2-ylmethyl di(phenanthren-9-yl)phosphinate (3aq)

Compound 3aq was isolated in 47% yield;**^{1}H NMR** (400 MHz, $CDCl_3$) δ 8.75 – 8.68 (m, 7H), 8.63 (s, 1H), 8.50 (d, *J* = 4.8 Hz, 1H), 7.92 (d, *J* = 7.8 Hz, 1H), 7.81 – 7.71 (m, 2H), 7.62 (dd, *J* = 14.6, 7.3 Hz, 2H), 7.56 – 7.47 (m, 5H), 7.20 – 7.11 (m, 3H),δ 7.22 – 7.08 (m, 1H), 5.36 (d, *J* = 7.9 Hz, 2H).**^{13}C NMR** (101 MHz, $CDCl_3$) δ 156.30 (d, *J* = 7.9 Hz), 149.20 (s), 137.57 (d, *J* = 10.1 Hz), 136.75 (s), 132.42 (d, *J* = 2.5 Hz), 130.65 (d, *J* = 10.2 Hz), 130.28 (t, *J* = 5.2 Hz), 130.05 (d, *J* = 15.6 Hz), 129.34 (s), 129.34 (s), 127.53 (d, *J* = 4.6 Hz), 127.39 (s), 127.15 (d, *J* = 2.6 Hz), 126.96 (s), 125.63 (s), 123.16 (s), 122.85 (s), 122.66 (s), 121.93 (s), 67.37 (d, *J* = 5.4 Hz).**^{31}P NMR** (162 MHz, $CDCl_3$) δ 35.86 (s).**HRMS (ESI):** [M+H] $^{+}$calcd for $C_{34}H_{24}NO_2P$ 510.1617, found 510.1612.

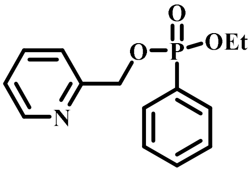

ethyl (pyridin-2-ylmethyl) phenylphosphonate (3ar)

Compound 3ar was isolated in 70% yield;^{1}**H NMR** (400 MHz, $CDCl_3$) δ 8.54 (d, *J* = 4.7 Hz, 1H), 7.91 – 7.81 (m, 2H), 7.70 (td, *J* = 7.7, 1.5 Hz, 1H), 7.60 – 7.53 (m, 1H), 7.48 (dt, *J* = 11.7, 5.8 Hz, 3H), 7.21 (dd, *J* = 7.2, 5.1 Hz, 1H), 5.18 (qd, *J* = 13.2, 7.6 Hz, 2H), 4.42 – 3.99 (m, 2H), 1.34 (t, *J* = 7.1 Hz, 3H).^{13}**C NMR** (101 MHz, $CDCl_3$) δ 156.30 (d, *J* = 8.3 Hz), 149.17 (s), 136.84 (s), 132.63 (d, *J* = 3.1 Hz), 131.85 (d, *J* = 10.0 Hz), 128.56 (d, *J* = 15.2 Hz), 126.78 (s), 122.83 (s), 121.30 (s), 67.76 (d, *J* = 5.0 Hz), 62.51 (d, *J* = 5.6 Hz), 16.35 (d, *J* = 6.4 Hz). ^{31}**P NMR** (162 MHz, $CDCl_3$) δ 19.38 (s).**HRMS (ESI):** $[M+H]^+$calcd for $C_{14}H_{16}NO_2P$ 278.0941, found 278.0947.

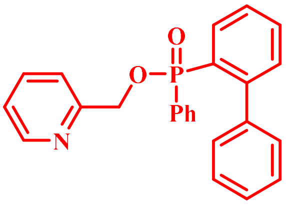

pyridin-2-ylmethyl [1,1'-biphenyl]-2-yl(phenyl)phosphinate (3as)

Compound 3as was isolated in 68% yield; ^{1}**H NMR** (400 MHz, $CDCl_3$) δ 8.63 (d, *J* = 4.3 Hz, 1H), 8.28 (ddd, *J* = 13.0, 7.7, 1.1 Hz, 1H), 7.78 (td, *J* = 7.7, 1.7 Hz, 1H), 7.69 (tt, *J* = 7.5, 1.4 Hz, 1H), 7.60 (tdd, *J* = 7.6, 2.7, 1.3 Hz, 1H), 7.48 (ddd, *J* = 11.0, 10.1, 7.5 Hz, 4H), 7.39 – 7.30 (m, 5H), 7.25 (q, *J* = 7.8 Hz, 4H), 5.17 (dd, *J* = 13.2, 7.4 Hz, 1H), 5.07 (dd, *J* = 13.2, 7.2 Hz, 1H).^{13}**C NMR** (101 MHz, $CDCl_3$) δ 156.59 (d, *J* = 8.3 Hz), 149.01 (s), 146.53 (d, *J* = 11.6 Hz), 140.45 (d, *J* = 4.3 Hz), 136.75 (s), 133.01 (d, *J* = 9.0 Hz), 131.99 (d, *J* = 2.8 Hz), 131.77 – 131.64 (m), 131.57 (d, *J* = 6.7 Hz), 131.42 (s), 130.63 (d, *J* = 7.7 Hz), 129.76 (s), 128.02 (d, *J* = 13.4 Hz), 127.33 (d, *J* = 10.0 Hz), 126.85 (d, *J* = 12.4 Hz), 122.69 (s), 121.57 (s), 66.47 (d, *J* = 5.0 Hz). ^{31}**P NMR** (162 MHz, $CDCl_3$) δ 33.10 (s).**HRMS (ESI):** $[M+H]^+$calcd for $C_{24}H_{20}NO_2P$ 386.1304, found 386.1305.

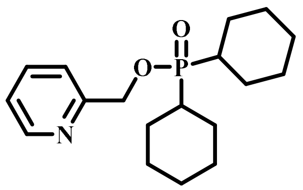

pyridin-2-ylmethyl dicyclohexylphosphinate (3at)

Compound 3at was isolated in 77% yield;[1]**H NMR** (400 MHz, $CDCl_3$) δ 8.57 (t, *J* = 6.7 Hz, 1H), 7.73 (td, *J* = 7.7, 1.6 Hz, 1H), 7.60 – 7.42 (m, 1H), 7.23 (dd, *J* = 7.2, 5.1 Hz, 1H), 5.16 (d, *J* = 6.9 Hz, 2H), δ 1.99 (d, *J* = 12.0 Hz, 2H), 1.85 (ddd, *J* = 14.1, 8.8, 3.0 Hz, 8H), 1.71 (s, 2H), 1.54 – 1.34 (m, 4H), 1.23 (t, *J* = 8.1 Hz, 6H).**^{13}C NMR** (101 MHz, $CDCl_3$) δ 157.30 (d, *J* = 6.5 Hz), 149.25 (s), 136.80 (s), 122.74 (s), 121.56 (s), 66.77 (d, *J* = 6.5 Hz), 36.59 (s), 35.72 (s), 26.56 – 26.10 (m), 25.90 (d, *J* = 1.4 Hz), 25.43 (dd, *J* = 5.2, 3.4 Hz).**^{31}P NMR** (162 MHz, $CDCl_3$) δ 60.33 (s).**HRMS (ESI):** $[M+H]^+$calcd for $C_{18}H_{28}NO_2P$ 322.1930, found 322.1924.

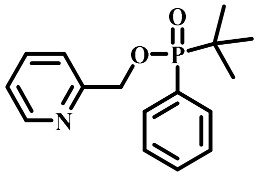

pyridin-2-ylmethyl tert-butyl(phenyl)phosphinate (3au)

Compound 3au was isolated in 65% yield;[1]**H NMR** (400 MHz, $CDCl_3$) δ 8.56 (d, *J* = 4.5 Hz, 1H), 7.80 (dd, *J* = 9.4, 8.0 Hz, 2H), 7.74 (td, *J* = 7.7, 1.5 Hz, 1H), 7.56 (t, *J* = 6.0 Hz, 2H), 7.47 (td, *J* = 7.4, 3.1 Hz, 2H), 7.23 (dd, *J* = 7.0, 5.2 Hz, 2H), 5.23 (dd, *J* = 13.2, 7.3 Hz, 1H), 4.94 (dd, *J* = 13.2, 6.6 Hz, 1H), 1.31 – 1.07 (m, 9H).**^{13}C NMR** (101 MHz, $CDCl_3$) δ 156.95 (d, *J* = 7.4 Hz), 149.17 (s), 136.84 (s), 133.22 (d, *J* = 8.9 Hz), 132.24 (d, *J* = 2.6 Hz), 128.38 (d, *J* = 11.7 Hz), 127.28 (s), 122.77 (s), 121.45 (s), 66.65 (d, *J* = 6.7 Hz), 33.34 (s), 32.35 (s), 24.31 (s).**^{31}P NMR** (162 MHz, $CDCl_3$) δ 52.83 (s).**HRMS (ESI):** $[M+H]^+$calcd for $C_{30}H_{24}NO_2P$ 290.1304, found 290.1309.

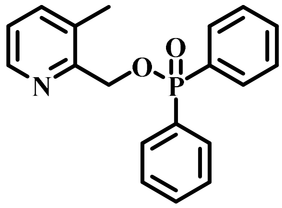

(3-methylpyridin-2-yl)methyl diphenylphosphinate (3ba)

Compound 3ba was isolated in 32% yield;^{1}**H NMR** (400 MHz, $CDCl_3$) δ 8.42 (d, *J* = 4.1 Hz, 1H), 7.91 – 7.73 (m, 4H), 7.51 (dt, *J* = 15.3, 4.4 Hz, 3H), 7.43 (td, *J* = 7.4, 3.5 Hz, 4H), 7.18 (dd, *J* = 7.6, 4.8 Hz, 1H), 5.20 (d, *J* = 6.5 Hz, 2H), 2.42 (s, 3H).^{13}**C NMR** (101 MHz, $CDCl_3$) δ 153.56 (d, *J* = 8.5 Hz), 146.80 (s), 138.43 (s), 133.24 (s), 132.23 (d, *J* = 2.8 Hz), 131.80 (t, *J* = 8.2 Hz), 130.52 (s), 128.54 (d, *J* = 13.2 Hz), 123.73 (s), 66.21 (d, *J* = 5.6 Hz), 18.21 (s). ^{31}**P NMR** (162 MHz, $CDCl_3$) δ 32.58 (s).**HRMS (ESI):** $[M+H]^+$calcd for $C_{19}H_{18}NO_2P$ 324.1148, found 324.1159.

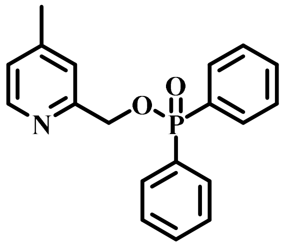

(4-methylpyridin-2-yl)methyl diphenylphosphinate (3bb)

Compound 3bb was isolated in 92% yield;^{1}**H NMR** (400 MHz, $CDCl_3$) δ 8.39 (d, *J* = 5.0 Hz, 1H), 7.93 – 7.79 (m, 4H), 7.53 (dd, *J* = 10.5, 4.3 Hz, 2H), 7.46 (td, *J* = 7.3, 3.5 Hz, 4H), 7.33 (s, 1H), 7.02 (d, *J* = 4.9 Hz, 1H), 5.13 (d, *J* = 7.5 Hz, 2H), 2.34 (s, 3H).^{13}**C NMR** (101 MHz, $CDCl_3$) δ 156.03 (d, *J* = 8.0 Hz), 148.98 (s), 148.13 (s), 132.35 (d, *J* = 2.8 Hz), 132.06 – 131.29 (m), 130.38 (s), 128.63 (d, *J* = 13.2 Hz), 123.88 (s), 122.61 (s), 66.98 (d, *J* = 5.5 Hz), 21.18 (s).^{31}**P NMR** (162 MHz, $CDCl_3$) δ 32.89 (s).**HRMS (ESI):** $[M+H]^+$calcd for $C_{19}H_{18}NO_2P$ 324.1148, found 324.1151.

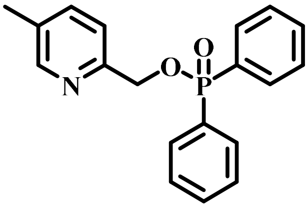

(5-methylpyridin-2-yl)methyl diphenylphosphinate (3bc)

Compound 3bc was isolated in 82% yield;**^{1}H NMR** (400 MHz, $CDCl_3$) δ 8.37 (s, 1H), 7.92 – 7.77 (m, 4H), 7.55 – 7.48 (m, 3H), 7.48 – 7.40 (m, 5H), 5.13 (d, *J* = 7.4 Hz, 2H), 2.26 (d, *J* = 41.0 Hz, 3H). **^{13}C NMR** (101 MHz, $CDCl_3$) δ 153.41 (d, *J* = 8.1 Hz), 149.59 (s), 137.31 (s), 132.45 (s), 132.28 (d, *J* = 2.8 Hz), 131.75 (t, *J* = 7.6 Hz), 130.46 (s), 128.59 (d, *J* = 13.2 Hz), 121.38 (s), 66.90 (d, *J* = 5.5 Hz), 18.19 (s).**^{31}P NMR** (162 MHz, $CDCl_3$) δ 32.81 (s). **HRMS (ESI):** $[M+H]^+$calcd for $C_{19}H_{18}NO_2P$ 324.1148, found 324.1146.

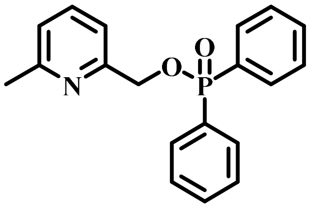

(6-methylpyridin-2-yl)methyl diphenylphosphinate (3bd)

Compound 3bd was isolated in45% yield;**^{1}H NMR** (400 MHz, $CDCl_3$) δ 7.91 – 7.81 (m, 4H), 7.58 (t, *J* = 7.7 Hz, 1H), 7.54 – 7.48 (m, 2H), 7.48 – 7.40 (m, 4H), 7.35 (d, *J* = 7.7 Hz, 1H), 7.05 (d, *J* = 7.7 Hz, 1H), 5.13 (d, *J* = 7.5 Hz, 2H), 2.50 (s, 3H). **^{13}C NMR** (101 MHz, $CDCl_3$) δ 157.86 (s), 155.75 (d, *J* = 8.1 Hz), 137.04 (s), 132.30 (d, *J* = 2.8 Hz), 131.74 (d, *J* = 10.3 Hz), 130.43 (s), 128.58 (d, *J* = 13.2 Hz), 122.39 (s), 118.35 (s), 66.97 (d, *J* = 5.4 Hz), 24.29 (s).**^{31}P NMR** (162 MHz, $CDCl_3$) δ 32.71 (s).**HRMS (ESI):** $[M+H]^+$calcd for $C_{19}H_{18}NO_2P$ 324.1148, found 324.1139.

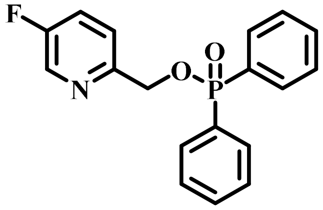

(5-fluoropyridin-2-yl)methyl diphenylphosphinate (3be)

Compound 3be was isolated in 67% yield;**^{1}H NMR** (400 MHz, $CDCl_3$) δ 8.39 (d, *J* = 2.8 Hz, 1H), 7.97 – 7.77 (m, 4H), 7.58 – 7.51 (m, 3H), 7.50 – 7.40 (m, 5H), 5.15 (d, *J* = 7.8 Hz, 2H).**^{13}C NMR** (101 MHz, $CDCl_3$) δ 160.18 (s), 157.64 (s), 152.32 (dd, *J* = 7.9, 3.9 Hz), 137.48 (d, *J* = 24.0 Hz), 132.45 (d, *J* = 2.8 Hz), 131.67 (t, *J* = 7.4 Hz),

130.25 (s), 128.68 (d, J = 13.2 Hz), 123.55 (d, J = 18.5 Hz), 122.96 (d, J = 4.5 Hz), 68.00 – 64.42 (m).^{19}**F NMR** (376 MHz, $CDCl_3$) δ -127.92 (s).^{31}**P NMR** (162 MHz, $CDCl_3$) δ 33.15 (s).**HRMS (ESI):** [M+H] + calcd for $C_{18}H_{15}FNO_2P$ 328.0897, found 328.0903.

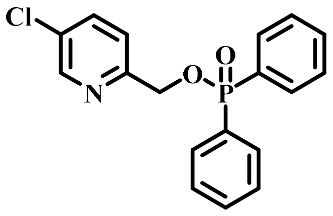

(5-chloropyridin-2-yl)methyl diphenylphosphinate (3bf)

Compound 3bf was isolated in 86% yield;^{1}**H NMR** (400 MHz, $CDCl_3$) δ 8.50 (d, J = 1.3 Hz, 1H), 7.88 (dd, J = 11.1, 8.3 Hz, 4H), 7.75 – 7.65 (m, 1H), 7.62 – 7.39 (m, 8H), 5.15 (dd, J = 7.7, 1.8 Hz, 2H).^{13}**C NMR** (101 MHz, $CDCl_3$) δ 154.61 (d, J = 8.0 Hz), 148.08 (s), 136.58 (s), 132.48 (d, J = 2.8 Hz), 131.70 (d, J = 10.3 Hz), 131.50 (s), 131.24 (s), 130.14 (s), 128.70 (d, J = 13.2 Hz), 122.53 (s), 66.28 (d, J = 5.3 Hz).^{31}**P NMR** (162 MHz, $CDCl_3$) δ 33.30 (s).**HRMS (ESI):** [M+H] + calcd for $C_{18}H_{15}ClNO_2P$ 344.0602, found 344.0607.

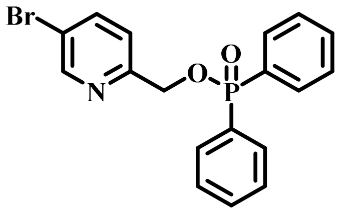

(5-bromopyridin-2-yl)methyl diphenylphosphinate (3bg)

Compound 3bg was isolated in 88% yield;^{1}**H NMR** (400 MHz, $CDCl_3$) δ 8.59 (d, J = 1.8 Hz, 1H), 7.93 – 7.77 (m, 5H), 7.54 (t, J = 7.1 Hz, 2H), 7.51 – 7.42 (m, 5H), 5.12 (d, J = 7.7 Hz, 2H).^{13}**C NMR** (101 MHz, $CDCl_3$) δ 155.02 (d, J = 7.9 Hz), 150.25 (s), 139.44 (s), 132.49 (d, J = 2.8 Hz), 131.70 (d, J = 10.3 Hz), 131.49 (s), 130.13 (s), 128.70 (d, J = 13.2 Hz), 123.02 (s), 119.89 (s), 66.30 (d, J = 5.3 Hz).^{31}**P NMR** (162 MHz, $CDCl_3$) δ 33.33 (s).**HRMS (ESI):** [M+H] + calcd for $C_{18}H_{15}BrNO_2P$ 388.0097,

found 388.0097.

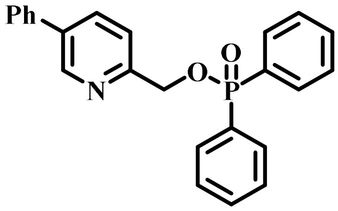

(5-phenylpyridin-2-yl)methyl diphenylphosphinate (3bh)

Compound 3bh was isolated in 88% yield;^{1}**H NMR** (400 MHz, $CDCl_3$) δ 8.80 (d, *J* = 1.8 Hz, 1H), 8.01 – 7.85 (m, 5H), 7.65 (d, *J* = 8.1 Hz, 1H), 7.61 – 7.53 (m, 4H), 7.53 – 7.47 (m, 6H), 7.43 (t, *J* = 7.3 Hz, 1H), 5.25 (d, *J* = 7.6 Hz, 2H).^{13}**C NMR** (101 MHz, $CDCl_3$) δ 155.12 (d, *J* = 8.0 Hz), 147.69 (s), 137.48 (s), 135.93 (s), 135.25 (s), 132.41 (d, *J* = 2.8 Hz), 131.76 (d, *J* = 10.2 Hz), 130.35 (s), 129.14 (s), 128.68 (d, *J* = 13.2 Hz), 128.21 (s), 127.14 (s), 121.71 (s), 66.83 (d, *J* = 5.4 Hz).^{31}**P NMR** (162 MHz, $CDCl_3$) δ 33.08 (s).**HRMS (ESI):** [M+H] + calcd for $C_{24}H_{20}NO_2P$ 386.1304, found 386.1301.

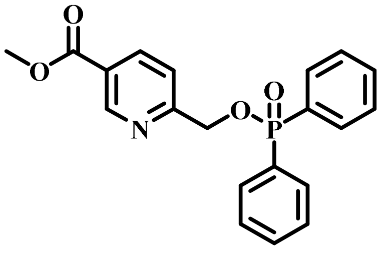

methyl 6-(((diphenylphosphoryl)oxy)methyl)nicotinate (3bi)

Compound 3bi was isolated in 94% yield;^{1}**H NMR** (400 MHz, $CDCl_3$) δ 9.13 (d, *J* = 1.6 Hz, 1H), 8.32 (dd, *J* = 8.2, 2.1 Hz, 1H), 7.96 – 7.83 (m, 4H), 7.66 (d, *J* = 8.2 Hz, 1H), 7.55 (td, *J* = 7.5, 1.3 Hz, 2H), 7.51 – 7.43 (m, 4H), 5.22 (d, *J* = 7.6 Hz, 2H), 4.19 – 3.45 (m, 4H).^{13}**C NMR** (101 MHz, $CDCl_3$) δ 165.50 (s), 160.86 (d, *J* = 8.0 Hz), 150.35 (s), 138.01 (s), 132.52 (d, *J* = 2.8 Hz), 131.69 (d, *J* = 10.3 Hz), 131.43 (s), 130.07 (s), 128.72 (d, *J* = 13.2 Hz), 125.14 (s), 120.75 (s), 66.44 (d, *J* = 5.3 Hz), 52.42 (s). ^{31}**P NMR** (162 MHz, $CDCl_3$) δ 33.45 (s).**HRMS (ESI):** [M+H] + calcd for $C_{20}H_{18}NO_4P$ 368.1046, found 368.1040.

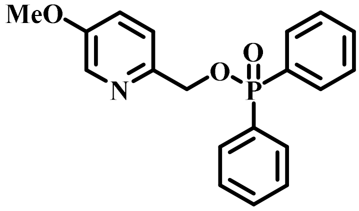

(5-methoxypyridin-2-yl)methyl diphenylphosphinate (3bj)

Compound 3bj was isolated in 52% yield;[1]**H NMR** (400 MHz, $CDCl_3$) δ 8.24 (d, *J* = 2.8 Hz, 1H), 7.89 – 7.80 (m, 4H), 7.55 – 7.49 (m, 2H), 7.48 – 7.41 (m, 5H), 7.19 (dd, *J* = 8.6, 2.9 Hz, 1H), 5.12 (d, *J* = 7.6 Hz, 2H), 3.85 (s, 3H).[13]**C NMR** (101 MHz, $CDCl_3$) δ 155.22 (s), 148.16 (d, *J* = 8.0 Hz), 136.99 (s), 132.29 (d, *J* = 2.8 Hz), 131.78 (t, *J* = 9.3 Hz), 130.51 (s), 128.60 (d, *J* = 13.2 Hz), 122.84 (s), 120.97 (s), 66.84 (d, *J* = 5.5 Hz), 55.65 (s).[31]**P NMR** (162 MHz, $CDCl_3$) δ 32.71 (s).**HRMS (ESI):** [M+H] + calcd for $C_{19}H_{18}NO_3P$ 340.1097, found 340.1090.

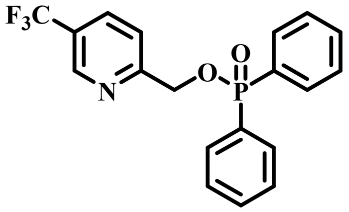

(5-(trifluoromethyl)pyridin-2-yl)methyl diphenylphosphinate (3bk)

Compound 3bk was isolated in 73% yield;[1]**H NMR** (400 MHz, $CDCl_3$) δ 8.80 (s, 1H), 7.95 (dd, *J* = 8.3, 2.0 Hz, 1H), 7.93 – 7.84 (m, 4H), 7.72 (d, *J* = 8.2 Hz, 1H), 7.60 – 7.53 (m, 2H), 7.48 (tdd, *J* = 8.2, 3.5, 1.2 Hz, 4H), 5.23 (d, *J* = 7.8 Hz, 2H). [13]**C NMR** (101 MHz, $CDCl_3$) δ 160.52 (d, *J* = 6.5 Hz), 146.09 (d, *J* = 4.0 Hz), 134.09 (d, *J* = 3.5 Hz), 132.59 (d, *J* = 2.8 Hz), 131.68 (d, *J* = 10.3 Hz), 131.34 (s), 129.97 (s), 128.75 (d, *J* = 13.3 Hz), 125.78 (d, *J* = 33.1 Hz), 124.76 (s), 122.05 (s), 121.03 (s), 66.29 (d, *J* = 5.2 Hz). [19]**F NMR** (376 MHz, $CDCl_3$) δ -54.50 – -70.75 (m).[31]**P NMR** (162 MHz, $CDCl_3$) δ 33.64 (s).**HRMS (ESI):** [M+H] + calcd for $C_{19}H_{15}F_3NO_2P$ 378.0865, found 378.0855.

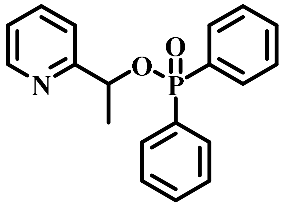

1-(pyridin-2-yl)ethyl diphenylphosphinate (3bl)

Compound 3bl was isolated in 63% yield;^{1}**H NMR** (400 MHz, $CDCl_3$) δ 8.54 – 8.48 (m, 1H), 7.93 – 7.83 (m, 2H), 7.78 – 7.70 (m, 2H), 7.65 (dd, *J* = 10.8, 4.5 Hz, 1H), 7.56 – 7.50 (m, 1H), 7.50 – 7.42 (m, 4H), 7.35 (ddd, *J* = 5.5, 4.4, 2.1 Hz, 2H), 7.20 – 7.13 (m, 1H), 5.57 (dq, *J* = 8.9, 6.5 Hz, 1H), 1.70 (d, *J* = 6.5 Hz, 3H).^{13}**C NMR** (101 MHz, $CDCl_3$) δ 160.75 (d, *J* = 5.3 Hz), 148.98 (s), 136.77 (s), 132.66 (s), 132.17 (d, *J* = 2.8 Hz), 132.06 (d, *J* = 2.8 Hz), 131.69 (dd, *J* = 10.2, 6.0 Hz), 131.29 (s), 130.79 (s), 122.66 (s), 120.48 (s), 74.93 (d, *J* = 5.5 Hz), 23.25 (d, *J* = 3.6 Hz).^{31}**P NMR** (162 MHz, $CDCl_3$) δ 31.47 (s).**HRMS (ESI):** [M+H] + calcd for $C_{19}H_{18}NO_2P$ 324.1148, found 324.1143.

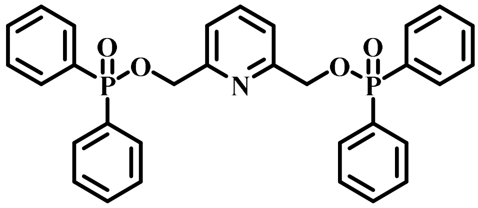

pyridine-2,6-diylbis(methylene) bis(diphenylphosphinate) (3bm)

Compound 3bm was isolated in 86% yield;^{1}**H NMR** (400 MHz, $CDCl_3$) δ 7.90 – 7.82 (m, 8H), 7.74 (t, *J* = 7.8 Hz, 1H), 7.56 – 7.50 (m, 4H), 7.49 – 7.41 (m, 10H), 5.10 (d, *J* = 7.5 Hz, 4H).^{13}**C NMR** (101 MHz, $CDCl_3$) δ 155.96 (d, *J* = 8.1 Hz), 137.75 (s), 132.42 (d, *J* = 2.8 Hz), 131.70 (t, *J* = 8.0 Hz), 130.27 (s), 128.66 (d, *J* = 13.2 Hz), 120.59 (s), 66.65 (d, *J* = 5.3 Hz).^{31}**P NMR** (162 MHz, $CDCl_3$) δ 33.05 (s).**HRMS (ESI):** [M+H] + calcd for $C_{31}H_{27}NO_4P_2$540.1488, found 540.1489.

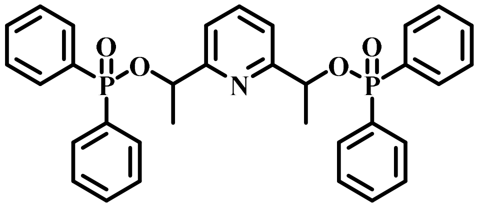

pyridine-2,6-diylbis(ethane-1,1-diyl) bis(diphenylphosphinate) (3bn)

Compound 3bn was isolated in 43% yield;^{1}**H NMR** (400 MHz, $CDCl_3$) δ 7.91 – 7.82 (m, 4H), 7.78 – 7.70 (m, 4H), 7.64 (t, J = 7.7 Hz, 1H), 7.56 – 7.49 (m, 2H), 7.49 – 7.41 (m, 6H), 7.35 (ddd, J = 9.4, 8.3, 4.8 Hz,6H), 5.60 – 5.38 (m, 2H), 1.71 – 1.51 (m, 6H).^{13}**C NMR** (101 MHz, $CDCl_3$) δ 160.06 (t, J = 5.1 Hz), 137.59 (d, J = 10.4 Hz), 132.63 (d, J = 7.5 Hz), 132.14 (d, J = 10.4 Hz), 131.70 (ddd, J = 10.1, 4.7, 3.2 Hz), 131.26 (d, J = 7.1 Hz), 130.77 (d, J = 7.9 Hz), 128.68 – 128.08 (m), 119.09 (d, J = 5.3 Hz), 74.89 (t, J = 5.7 Hz), 23.24 (dd, J = 6.6, 3.5 Hz).^{31}**P NMR** (162 MHz, $CDCl_3$) δ 31.30 (d, J = 8.7 Hz).**HRMS (ESI):** [M+H] + calcd for $C_{33}H_{31}NO_4P_2$568.1801, found 568.1785.

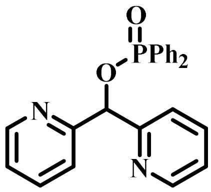

di(pyridin-2-yl)methyl diphenylphosphinate (3bo)

Compound 3bo was isolated in 73% yield;^{1}**H NMR** (400 MHz, $CDCl_3$) δ 8.47 (d, J = 4.5 Hz, 2H), 7.86 – 7.74 (m, 4H), 7.62 (q, J = 7.6 Hz, 4H), 7.46 (t, J = 7.4 Hz, 2H), 7.36 (td, J = 7.5, 3.5 Hz, 4H), 7.11 (dd, J = 8.4, 3.3 Hz, 2H), 6.58 (d, J = 10.1 Hz, 1H).^{13}**C NMR** (101 MHz, $CDCl_3$) δ 158.62 (d, J = 4.7 Hz), 149.35 (s), 136.72 (s), 132.20 (d, J = 2.8 Hz), 131.83 (t, J = 8.8 Hz), 130.55 (s), 128.39 (d, J = 13.3 Hz), 122.82 (s), 122.10 (s), 79.38 (d, J = 5.2 Hz).^{31}**P NMR** (162 MHz, $CDCl_3$) δ 32.85 (s).**HRMS (ESI):** [M+H] + calcd for $C_{23}H_{19}N_2O_2P$ 387.1257, found 387.1262.

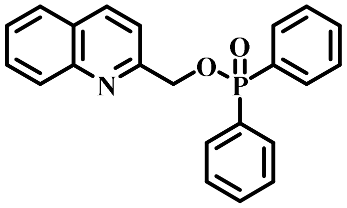

quinolin-2-ylmethyl diphenylphosphinate (3bp)

Compound 3bp was isolated in88% yield;^{1}**H NMR** (400 MHz, $CDCl_3$) δ 8.17 (d, *J* = 8.5 Hz, 1H), 8.01 (d, *J* = 8.5 Hz, 1H), 7.95 – 7.85 (m, 4H), 7.82 – 7.77 (m, 1H), 7.74 – 7.65 (m, 2H), 7.51 (ddd, *J* = 7.2, 4.7, 2.4 Hz, 3H), 7.48 – 7.41 (m, 4H), 5.33 (d, *J* = 7.5 Hz,2H). ^{13}**C NMR** (101 MHz, $CDCl_3$) δ 156.73 (d, *J* = 8.1 Hz), 147.41 (s), 137.09 (s), 132.42 (d, *J* = 2.8 Hz), 131.74 (t, *J* = 7.6 Hz), 130.31 (s), 129.81 (s), 129.05 (s), 128.67 (d, *J* = 13.2 Hz), 127.63 (d, *J* = 9.5 Hz), 126.69 (s), 119.42 (s), 67.56 (d, *J* = 5.5 Hz).^{31}**P NMR** (162 MHz, $CDCl_3$) δ33.22 (s).**HRMS (ESI):** [M+H] + calcd for $C_{22}H_{18}NO_2P$ 360.1148, found 360.1149.

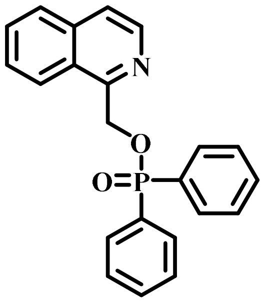

isoquinolin-1-ylmethyl diphenylphosphinate (3bq)

Compound 3bq was isolated in94% yield;^{1}**H NMR** (400 MHz, $CDCl_3$) δ 8.46 (dd, *J* = 8.5, 4.5 Hz, 2H), 7.91 – 7.76 (m, 5H), 7.72 – 7.58 (m, 3H), 7.48 (dd, *J* = 10.3, 4.5 Hz, 2H), 7.40 (dt, *J* = 10.4, 4.1 Hz, 4H), 5.63 (d, *J* = 6.4 Hz, 2H).^{13}**C NMR** (101 MHz, $CDCl_3$) δ 154.76 (d, *J* = 8.8 Hz), 141.74 (s), 136.48 (s), 132.31 (d, *J* = 2.8 Hz), 131.73 (t, *J* = 7.6 Hz), 130.33 (s), 128.56 (d, *J* = 13.2 Hz), 127.92 (s), 127.18 (s), 125.51 (s), 121.84 (s), 66.31 (d, *J* = 5.5 Hz).^{31}**P NMR** (162 MHz, $CDCl_3$) δ 33.09 (s).**HRMS (ESI):** [M+H] + calcd for $C_{22}H_{18}NO_2P$ 360.1148, found 360.1148.

7. X-ray crystallographic data for 3ak.

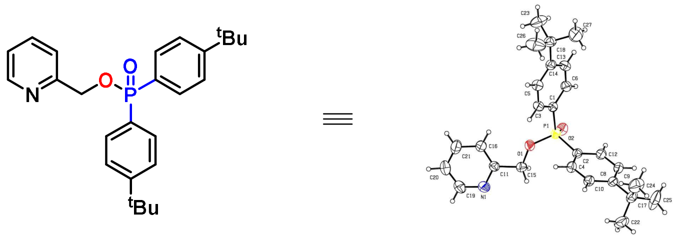

Figure S1. The structure of **3ak**

Table S1. Crystal data and structure refinement for **3ak**

Identification code	3ak
Empirical formula	$C_{26}H_{32}NO_2P$
Formula weight	421.49
Temperature/K	300.12(10)
Crystal system	monoclinic
Space group	$P2_1$
a/Å	12.3652(2)
b/Å	8.67120(10)
c/Å	23.3663(3)
α/°	90
β/°	103.9070(10)
γ/°	90
Volume/Å^3	2431.92(6)
Z	4
ρcalcg/cm^3	1.151
μ/mm^{-1}	1.155
F(000)	904.0
Crystal size/mm^3	0.14 × 0.11 × 0.08
Radiation	Cu Kα (λ = 1.54184)
2Θ range for data collection/°	9.354 to 154.844
Index ranges	-15 ≦ h ≦ 15, -10 ≦ k ≦ 10, -25 ≦ l ≦29
Reflections collected	34396
Independent reflections	4990 [Rint = 0.0616, Rsigma = 0.0387]
Data/restraints/parameters	4990/0/277
Goodness-of-fit on F^2	1.044
Final R indexes [I>=2σ (I)]	R1 = 0.0567, wR2 = 0.1617
Final R indexes [all data]	R1 = 0.0617, wR2 = 0.1669
Largest diff. peak/hole / e Å^{-3}	0.40/-0.34

Single crystals of $C_{26}H_{32}NO_2P$ [yangjin309_1102_auto] were collected. A suitable crystal was selectedand collected on a SuperNova, Dual, Cu at zero, Eos diffractometer. The crystal was kept at 300 Kduring data collection. Using Olex2, the structure was solved with the SHELXT structure solutionprogram using Intrinsic Phasing and refined with the SHELXL refinement package using Least Squaresminimisation. Refined structure and crystallographic parameters are summarized in Figure S1 andTableS2. CCDC 2177793 contains the supplementary crystallographic data for yangjin309_1102_auto.Thecrystallographic data of the compound can be obtained free of charge from The CambridgeCrystallographic Data Centre via http://www.ccdc.cam.ac.uk/data_request/cif.

8. Scanned ^{1}H NMR, ^{13}C NMR, ^{31}P NMR and ^{19}F NMR Spectra of All products

^{1}H NMR of **3aa** (400 MHz, $CDCl_3$)

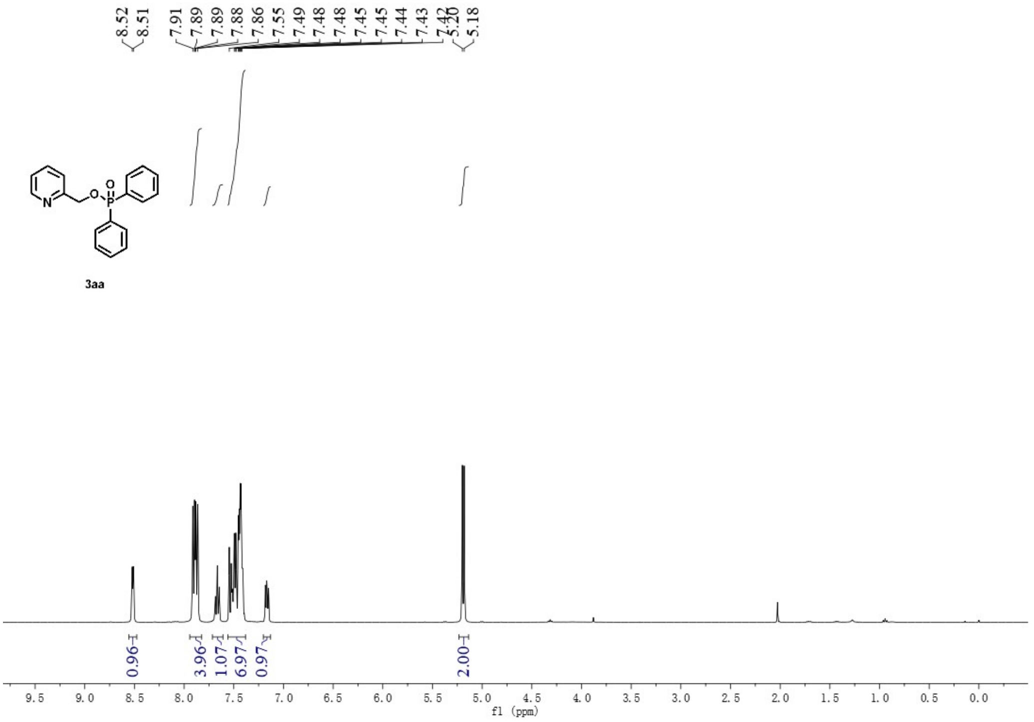

^{13}C NMR of **3aa** (101 MHz, $CDCl_3$)

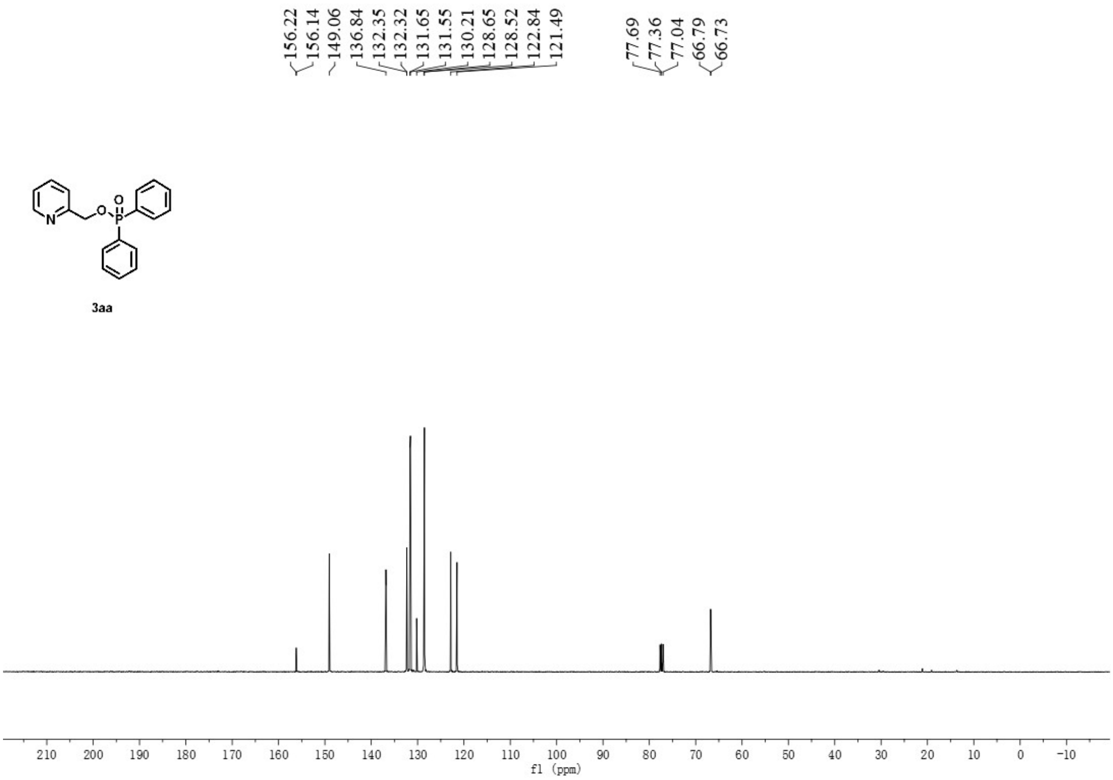

^{31}P NMR of **3aa** (121 MHz, $CDCl_3$)

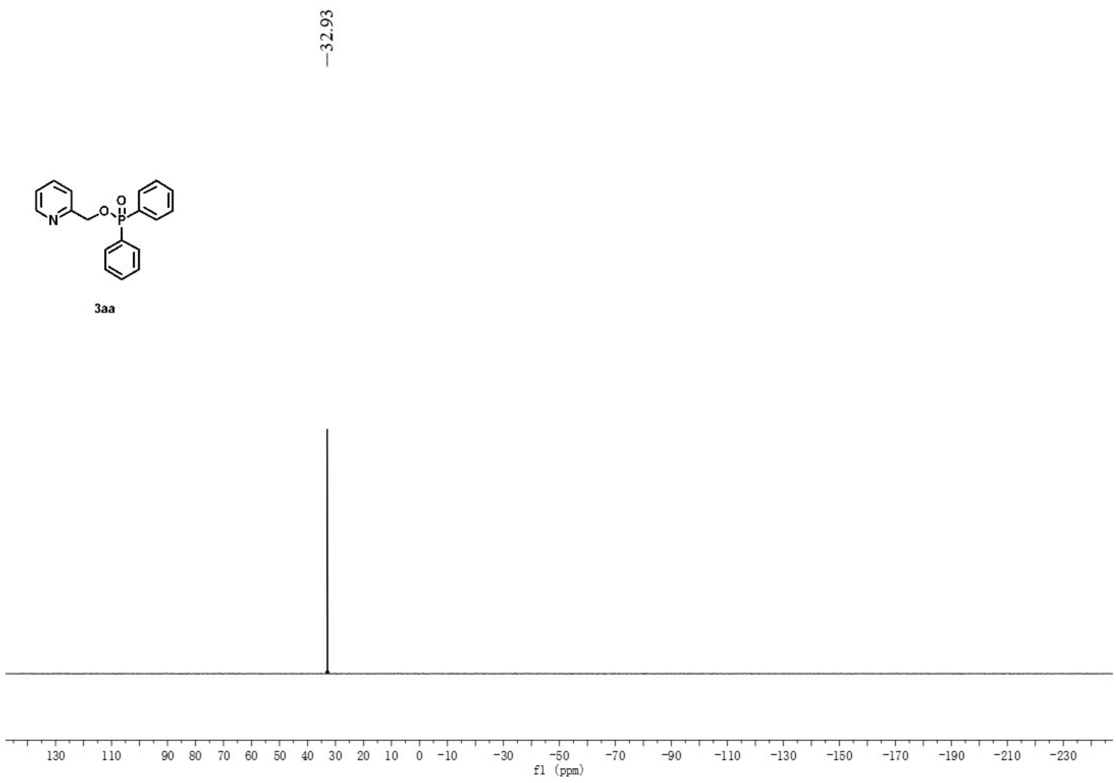

^{1}H NMR of **3ab** (400 MHz, $CDCl_3$)

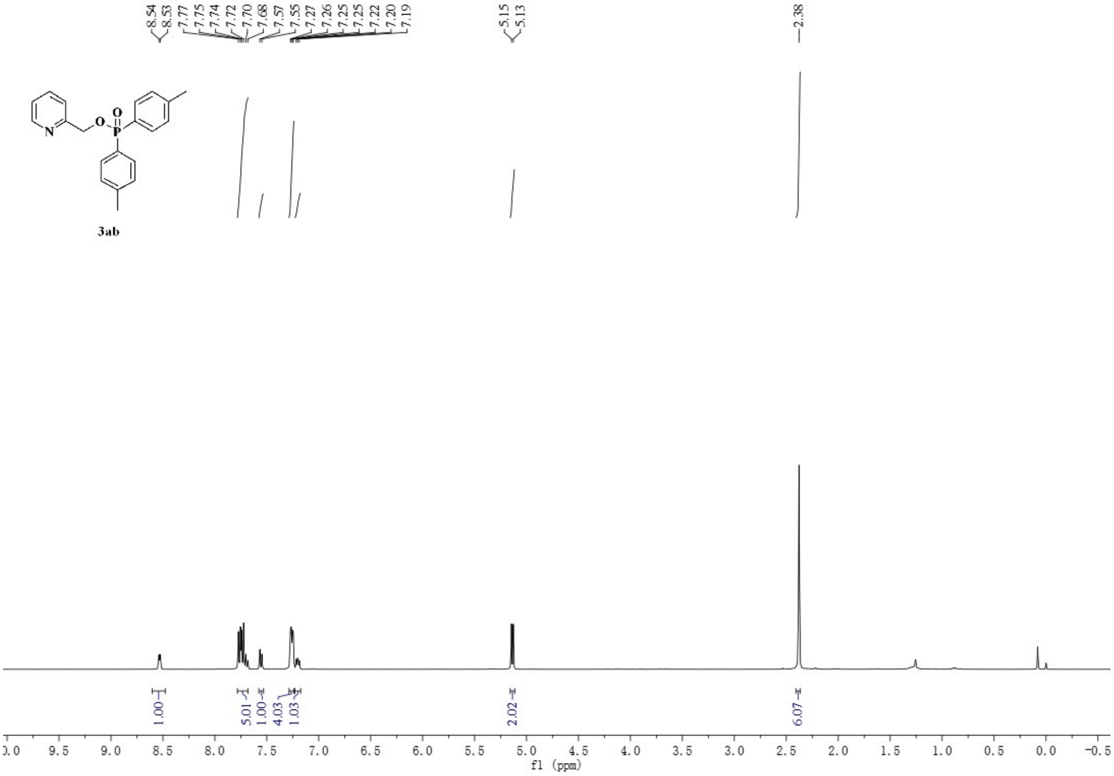

^{13}C NMR of **3ab** (101 MHz, $CDCl_3$)

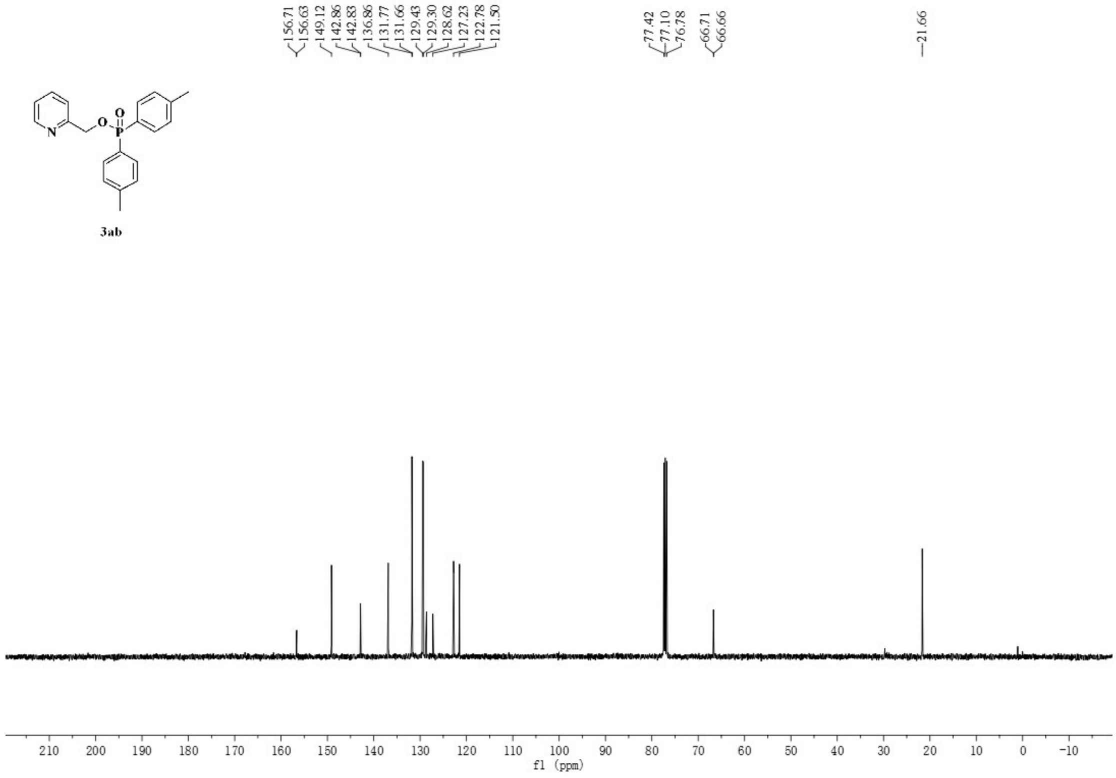

^{31}P NMR of **3ab** (121 MHz, $CDCl_3$)

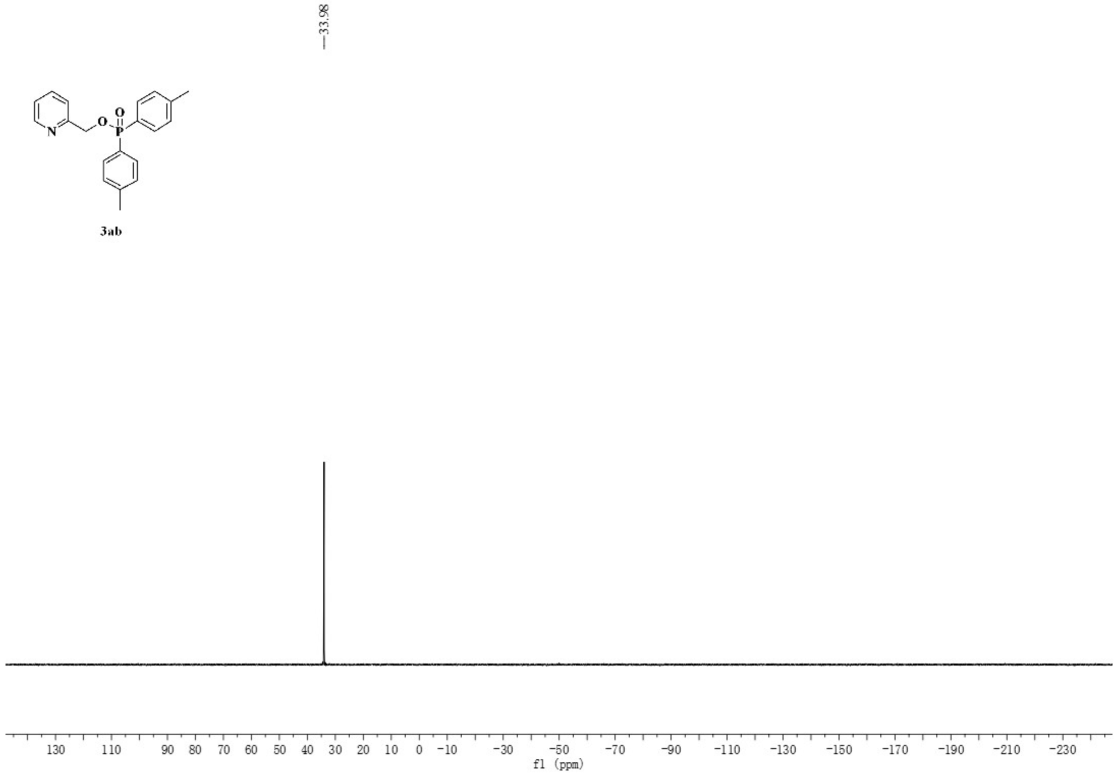

^{1}H NMR of **3ac** (400 MHz, $CDCl_3$)

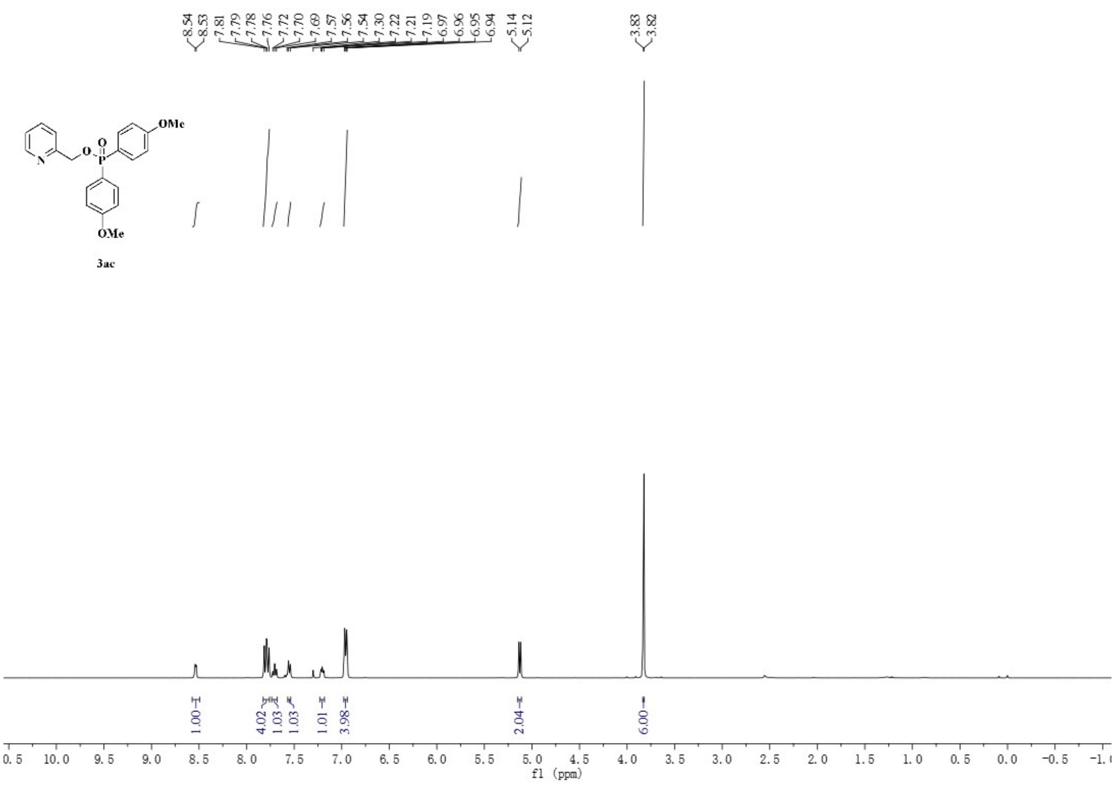

^{13}C NMR of **3ac** (101 MHz, $CDCl_3$)

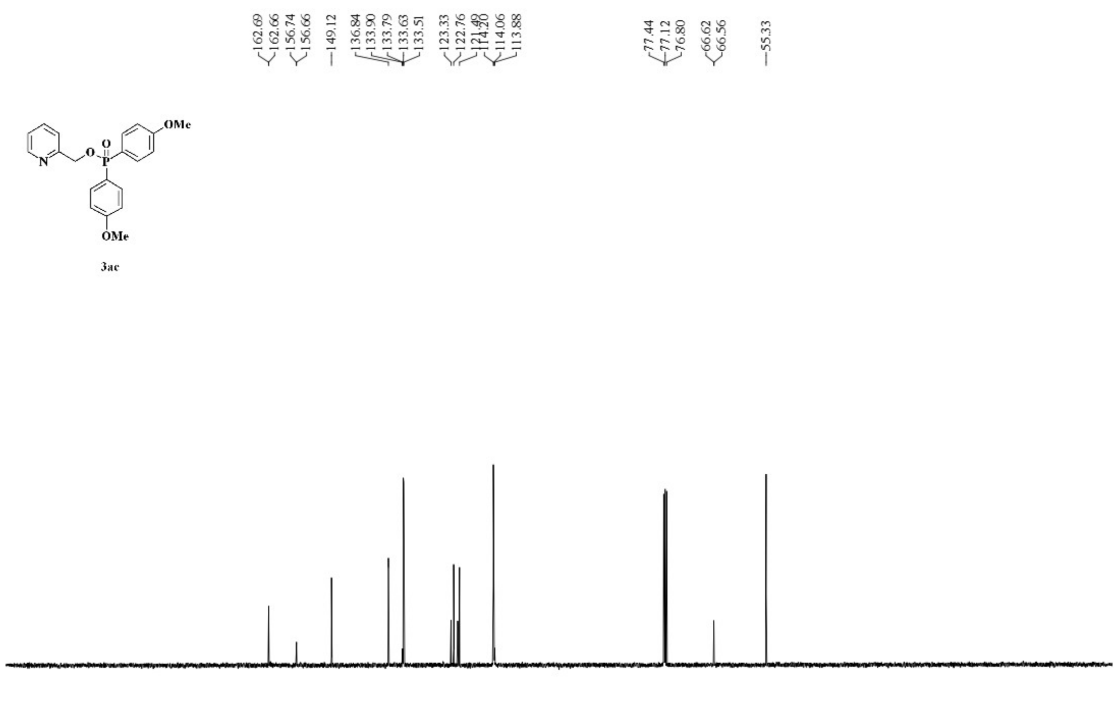

^{31}P NMR of **3ac** (121 MHz, $CDCl_3$)

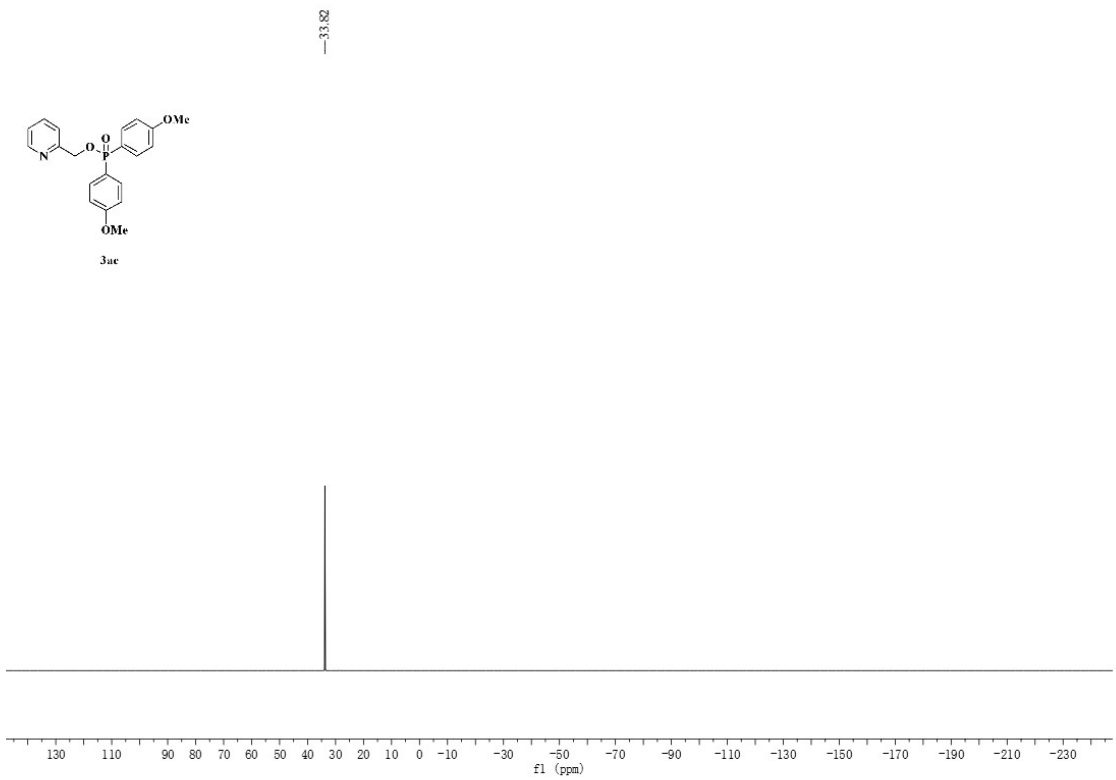

^{1}H NMR of **3ad** (400 MHz, $CDCl_3$)

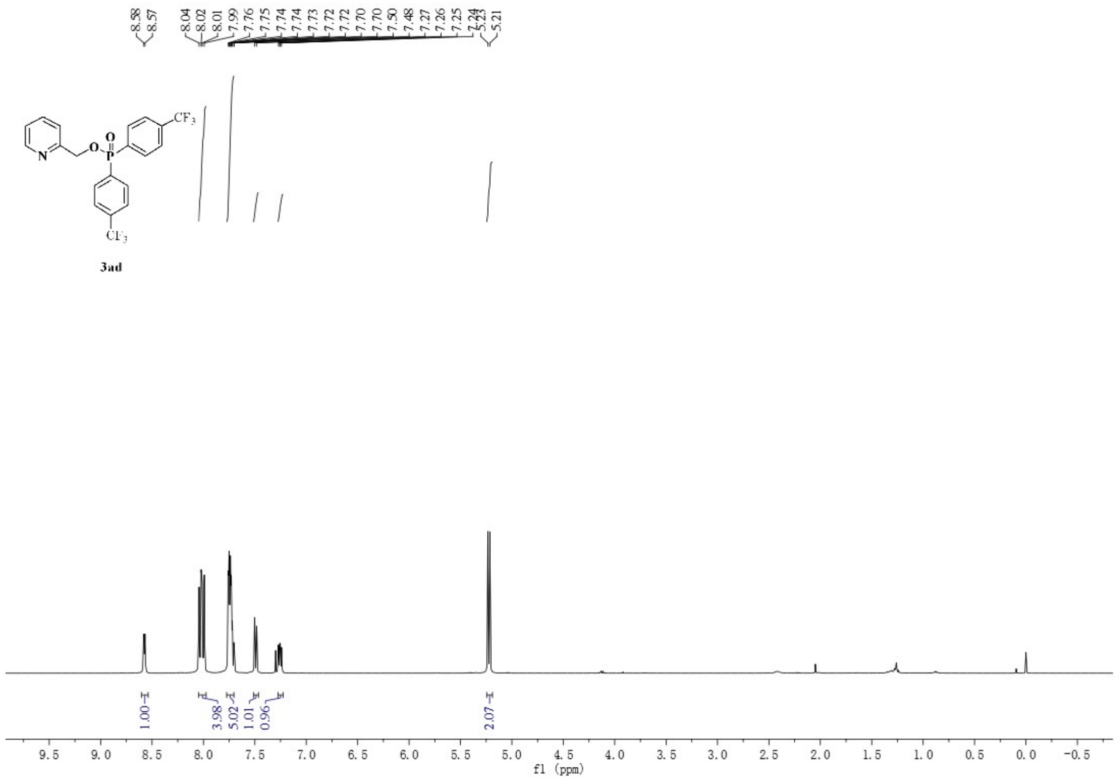

^{13}C NMR of **3ad** (101 MHz, $CDCl_3$)

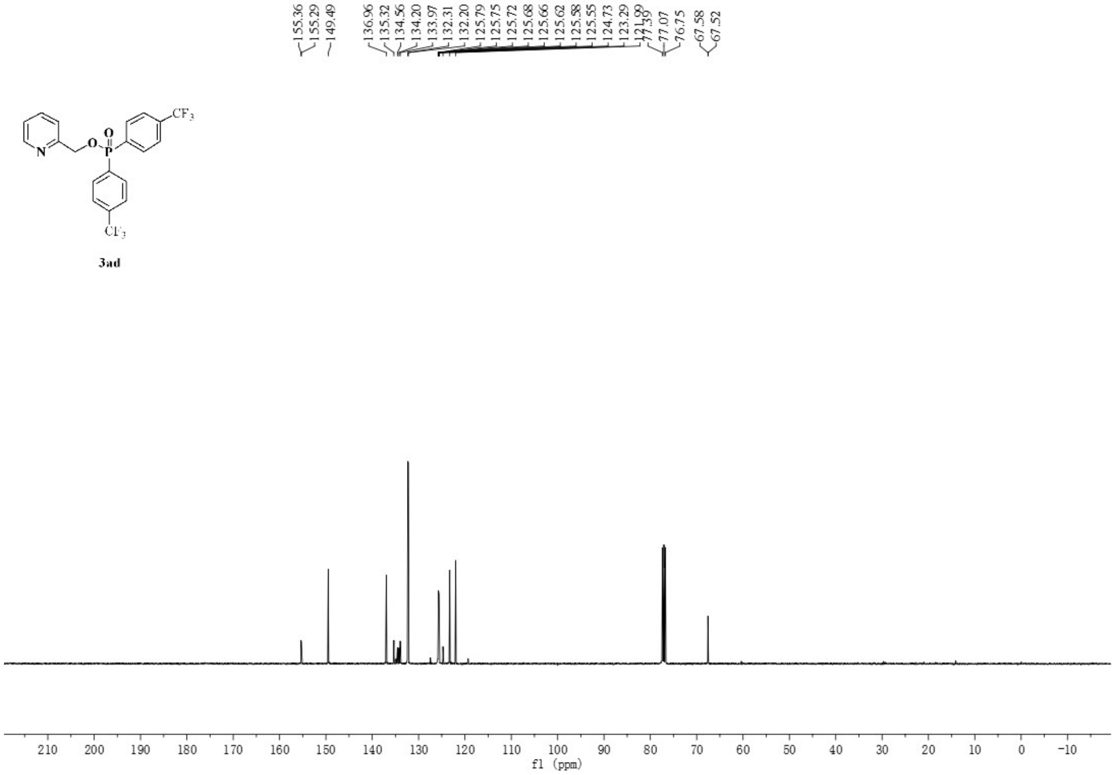

^{19}F NMR of **3ad** (282 MHz, $CDCl_3$)

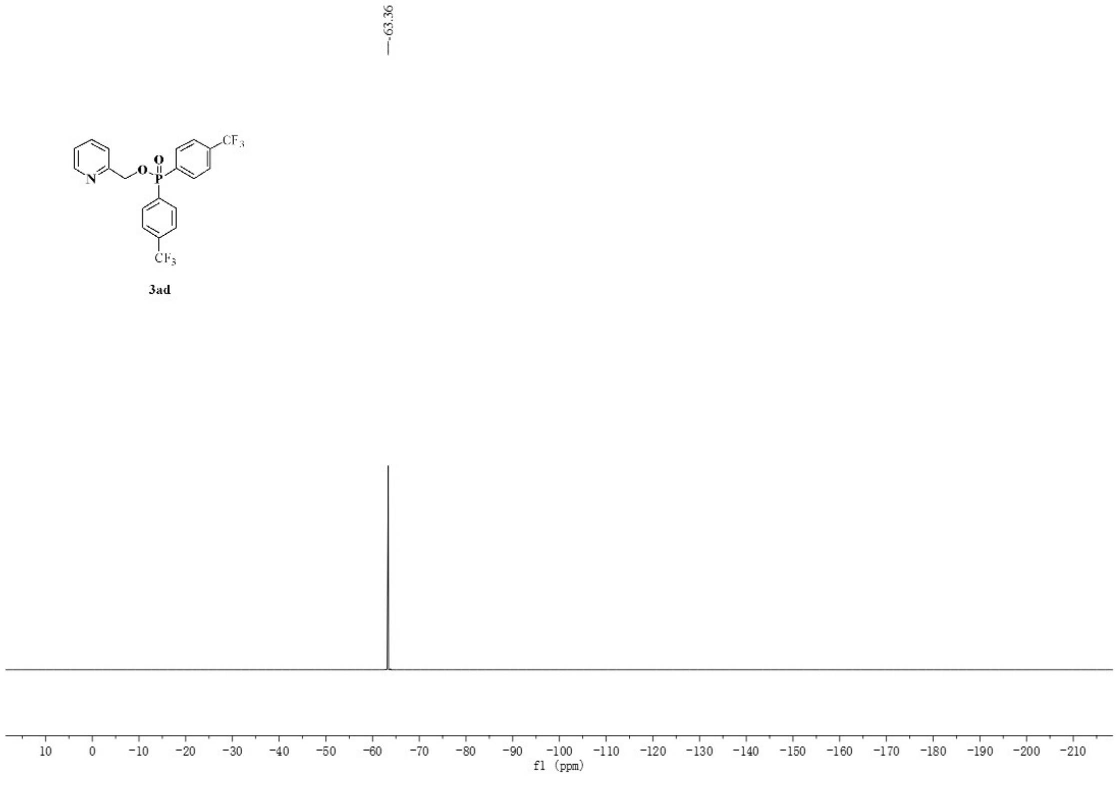

^{31}P NMR of **3ad** (121 MHz, $CDCl_3$)

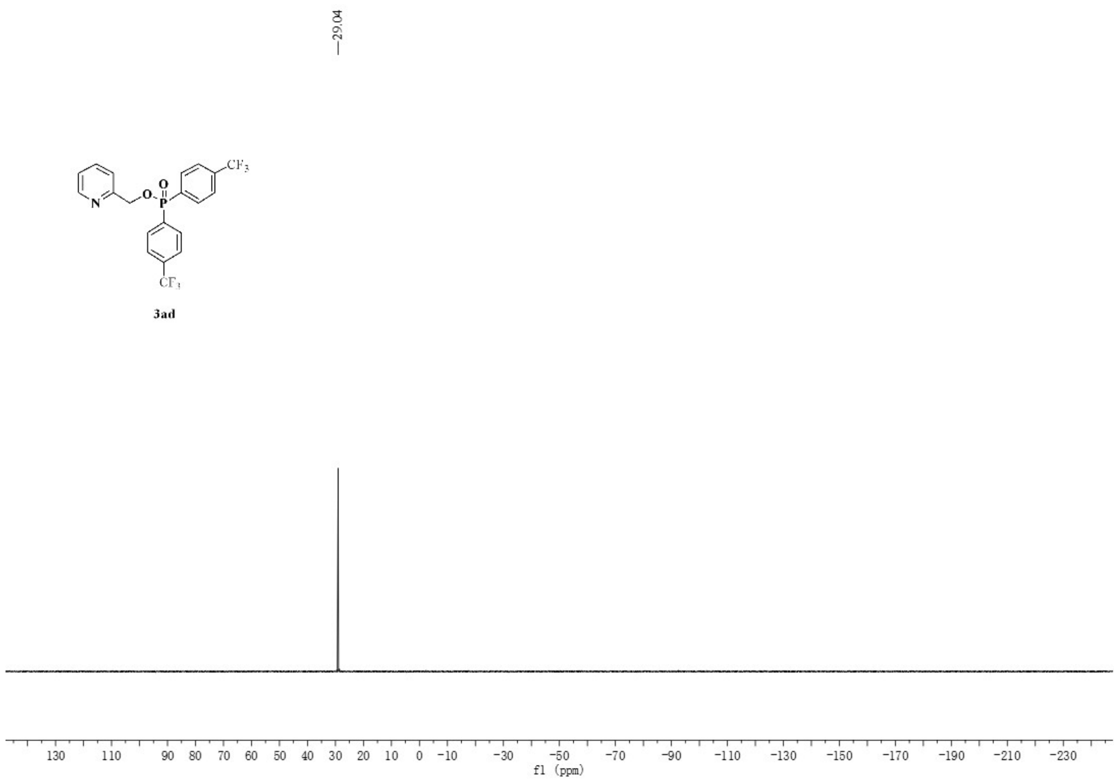

^{1}H NMR of **3ae** (400 MHz, $CDCl_3$)

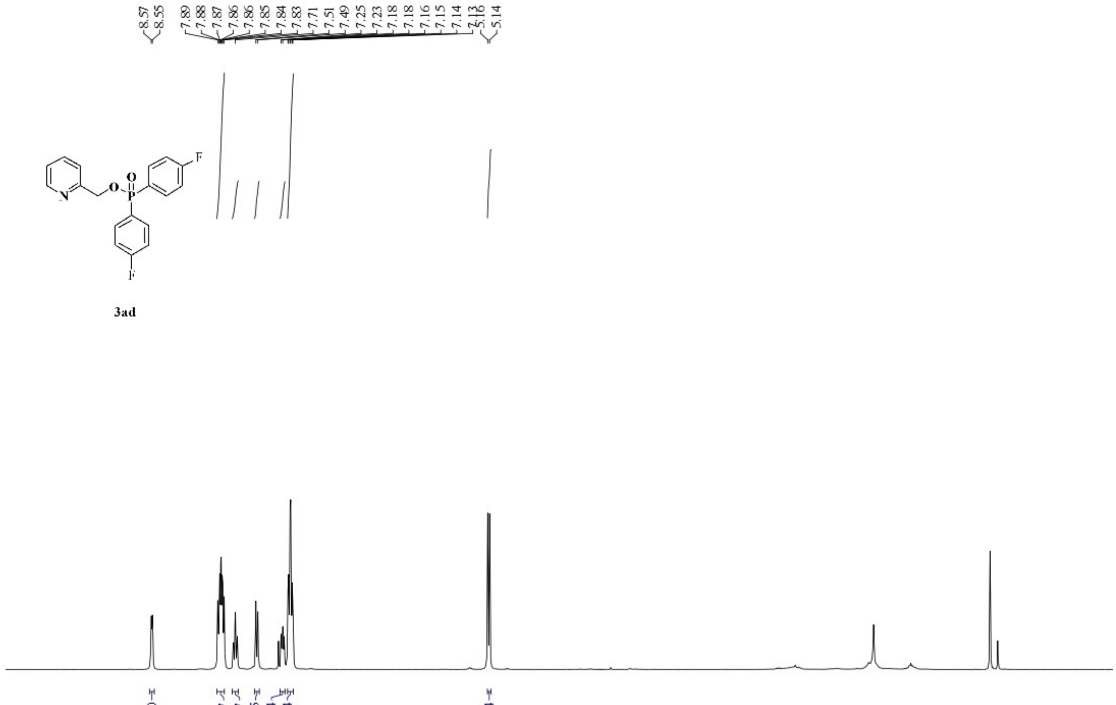

^{13}C NMR of **3ae** (101 MHz, $CDCl_3$)

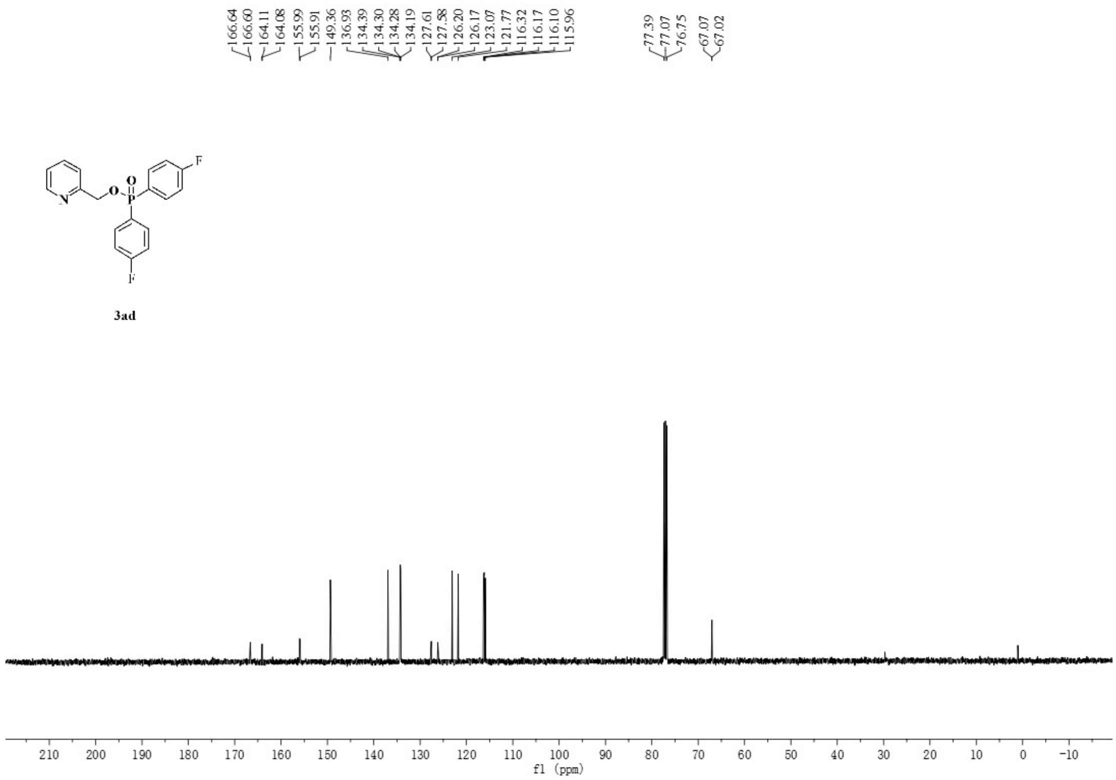

^{19}F NMR of **3ae** (282 MHz, $CDCl_3$)

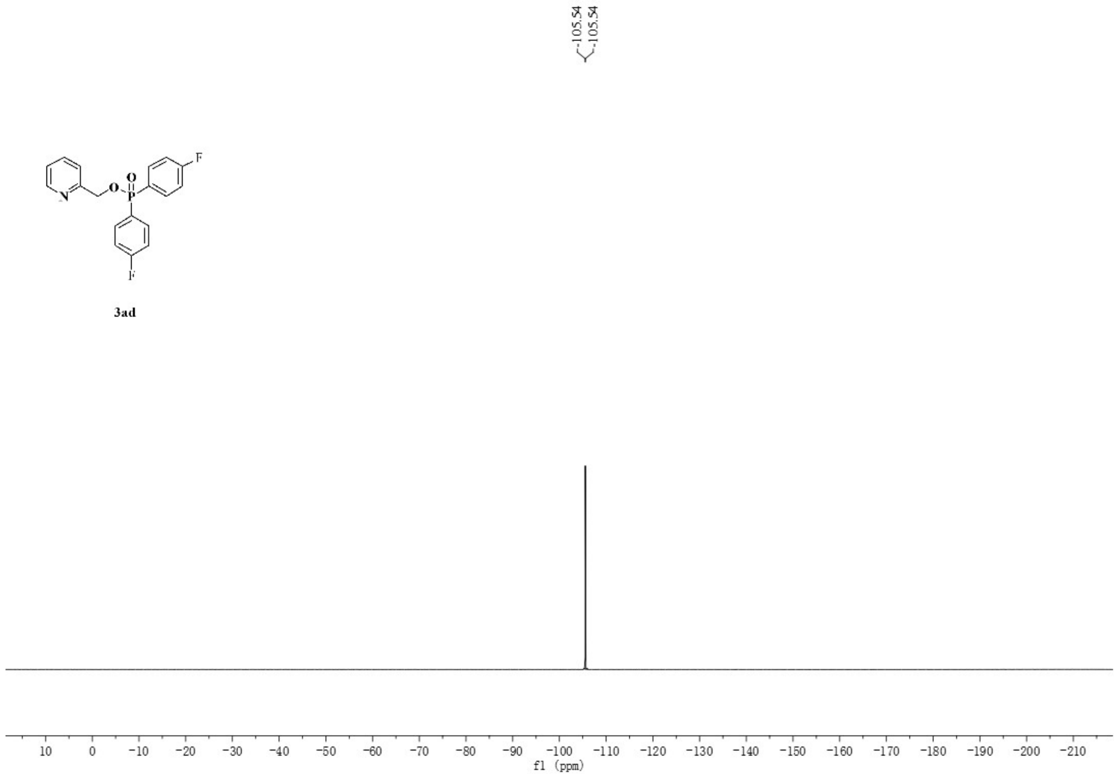

^{31}P NMR of **3ae** (121 MHz, $CDCl_3$)

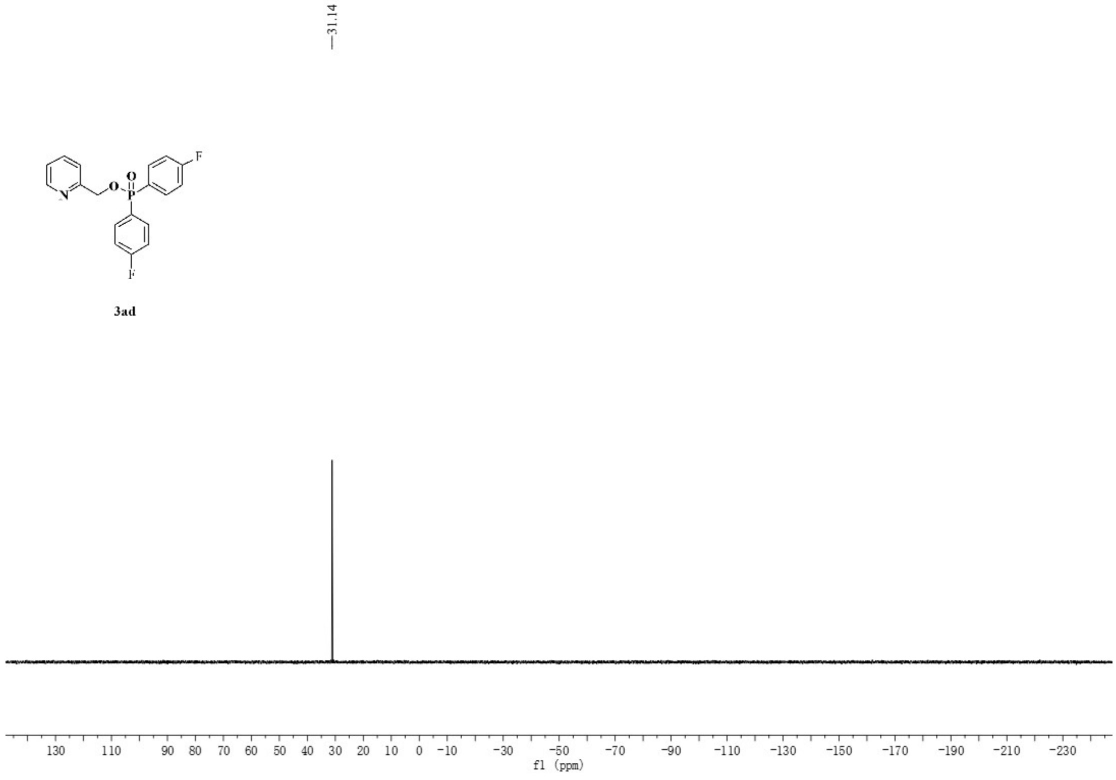

^{1}H NMR of **3af** (400 MHz, $CDCl_3$)

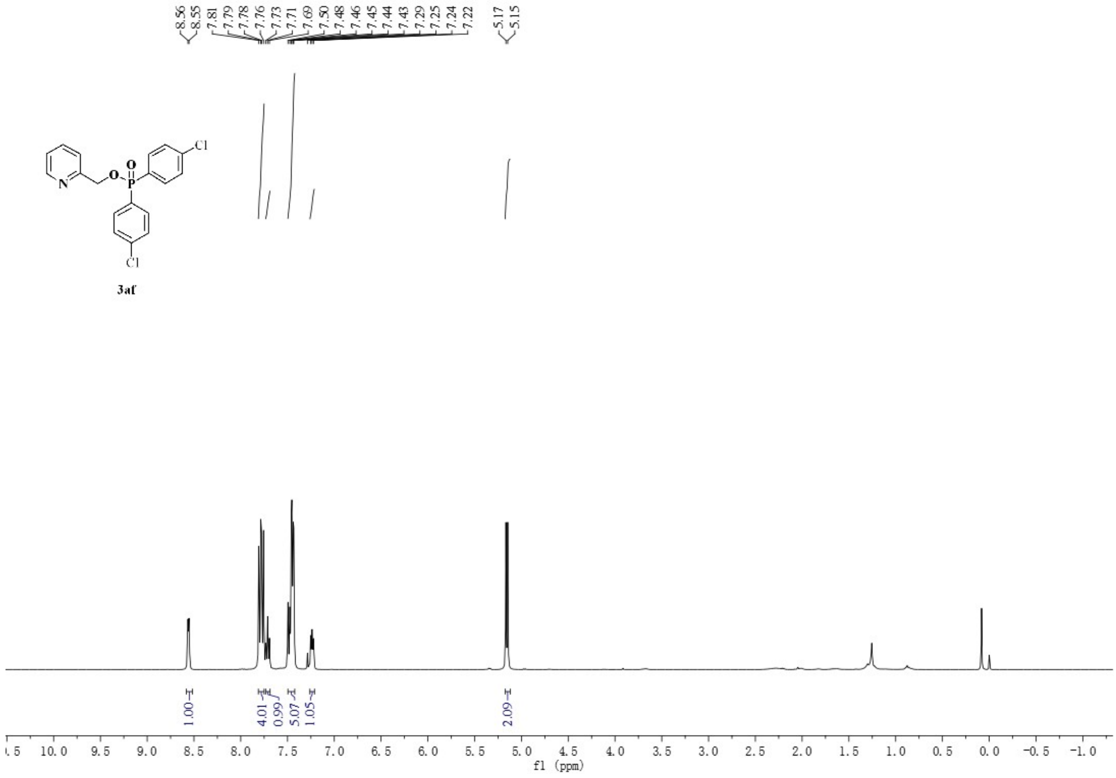

^{13}C NMR of **3af** (101 MHz, $CDCl_3$)

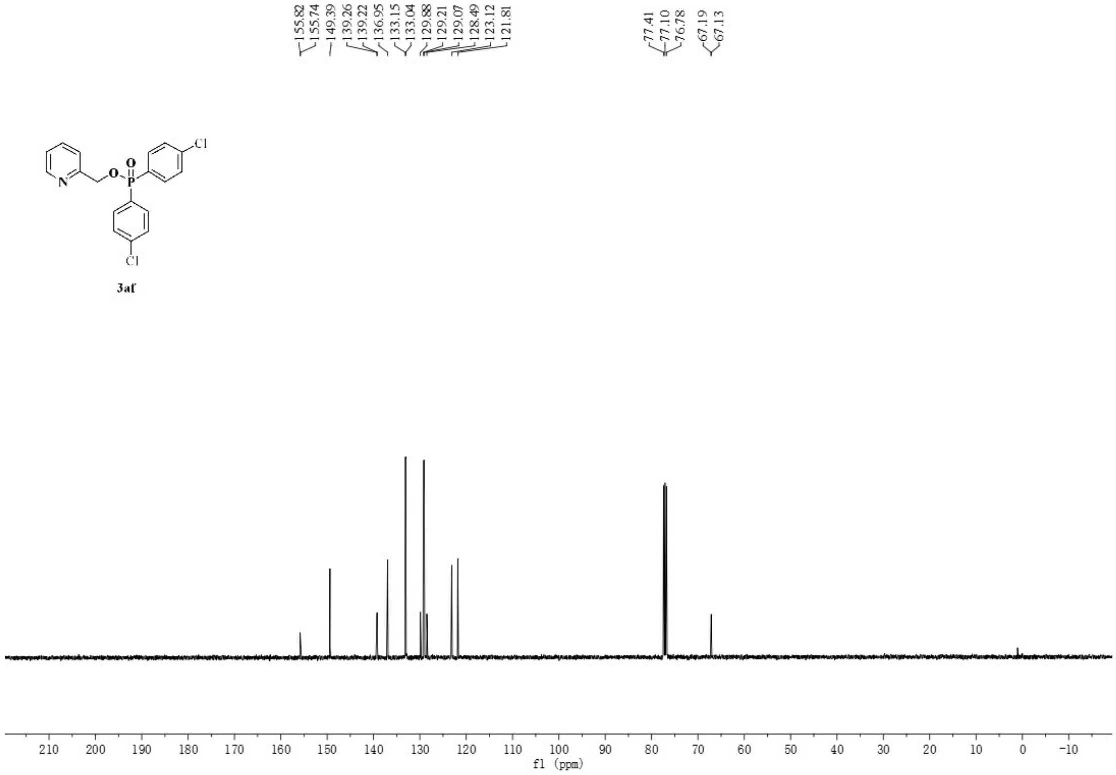

^{31}P NMR of **3af** (121 MHz, $CDCl_3$)

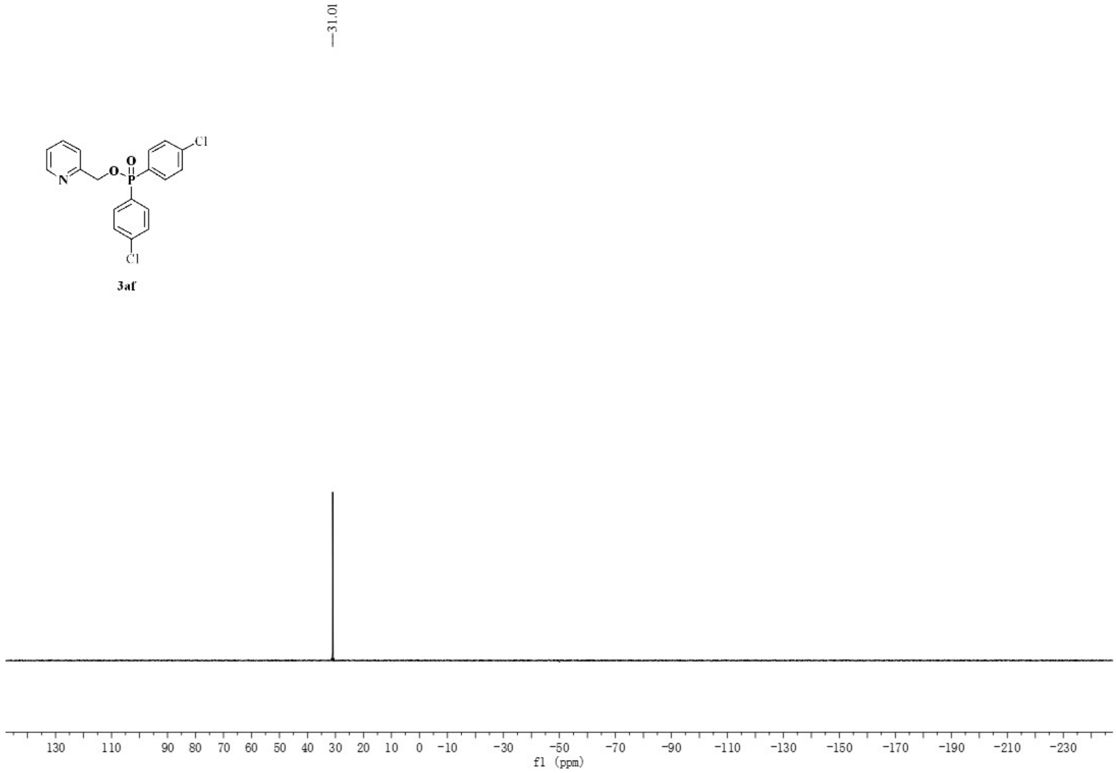

^{1}H NMR of **3ag** (400 MHz, $CDCl_3$)

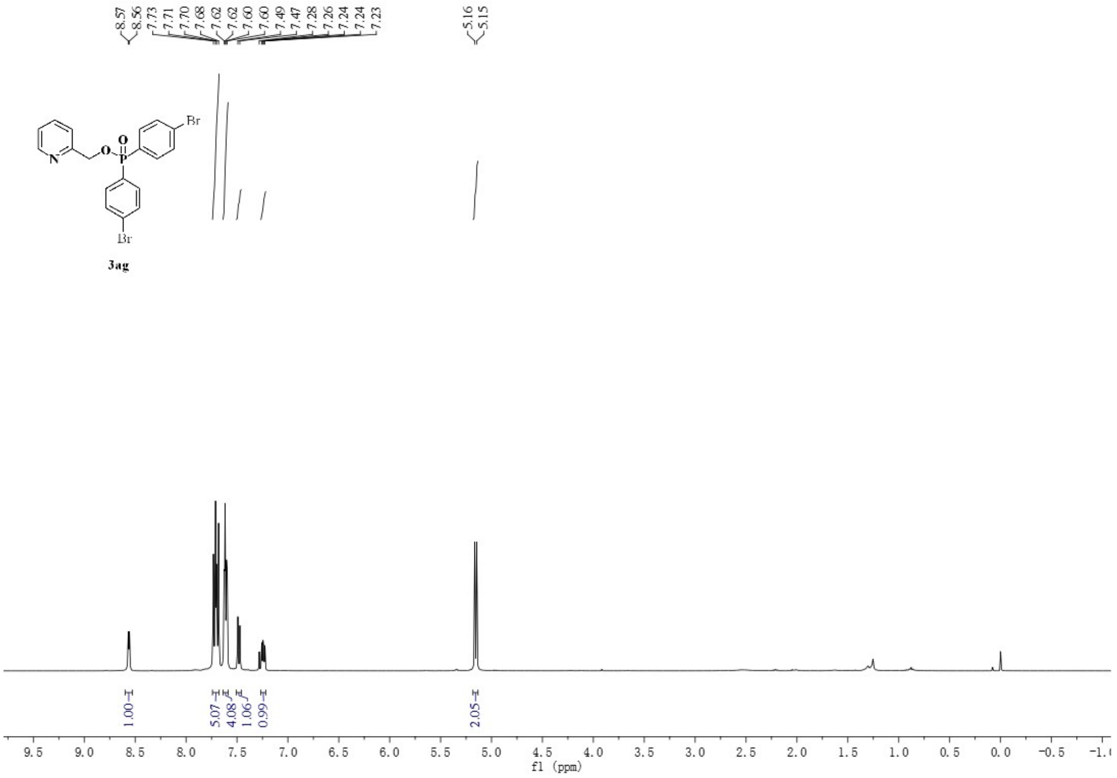

^{13}C NMR of **3ag** (101 MHz, $CDCl_3$)

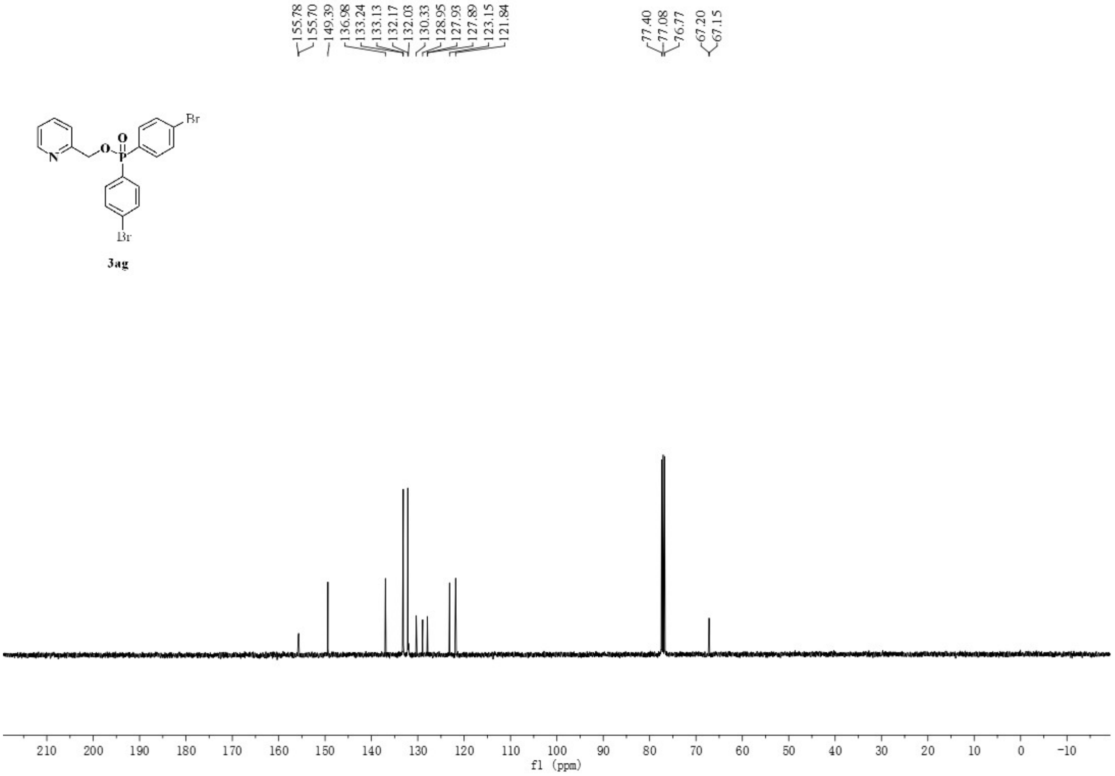

^{31}P NMR of **3ag** (121 MHz, $CDCl_3$)

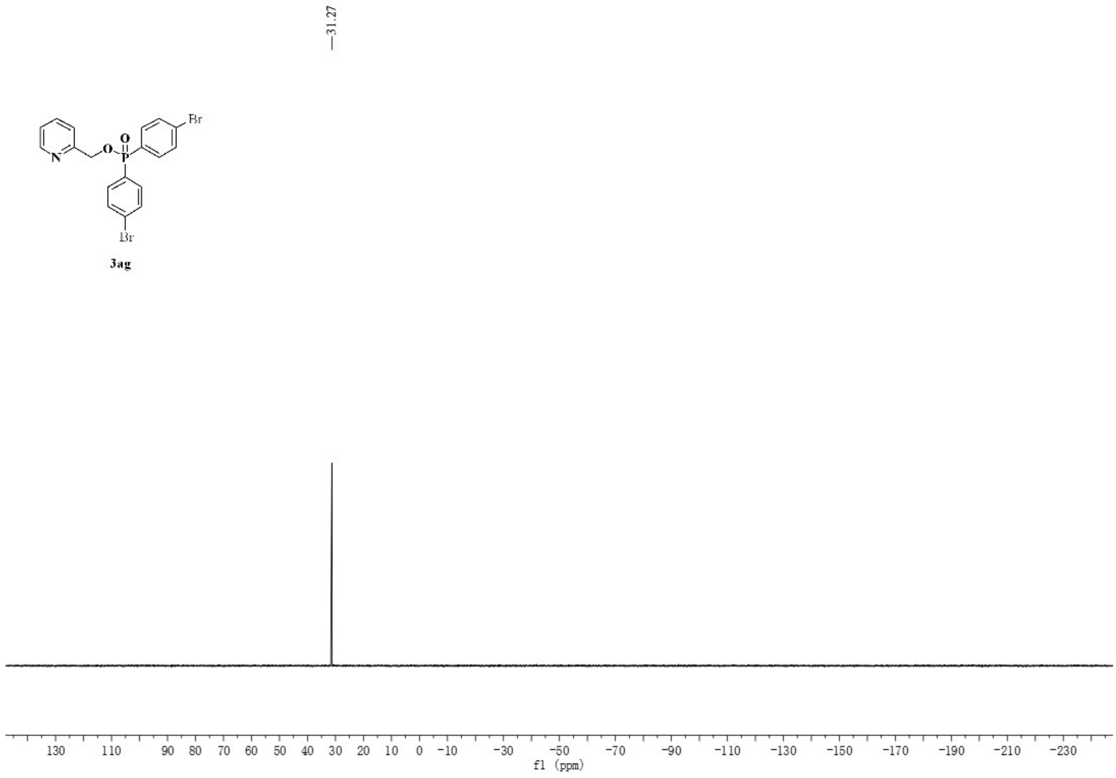

^{1}H NMR of **3ah** (400 MHz, $CDCl_3$)

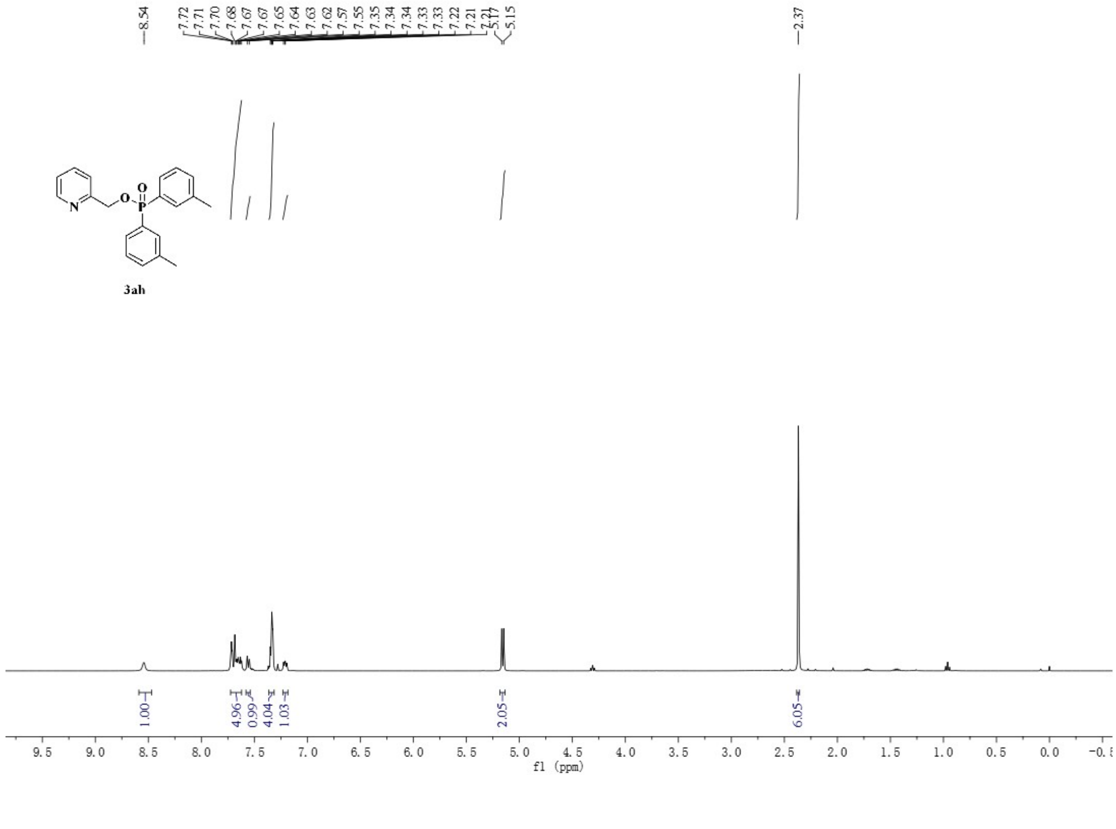

^{13}C NMR of **3ah** (101 MHz, $CDCl_3$)

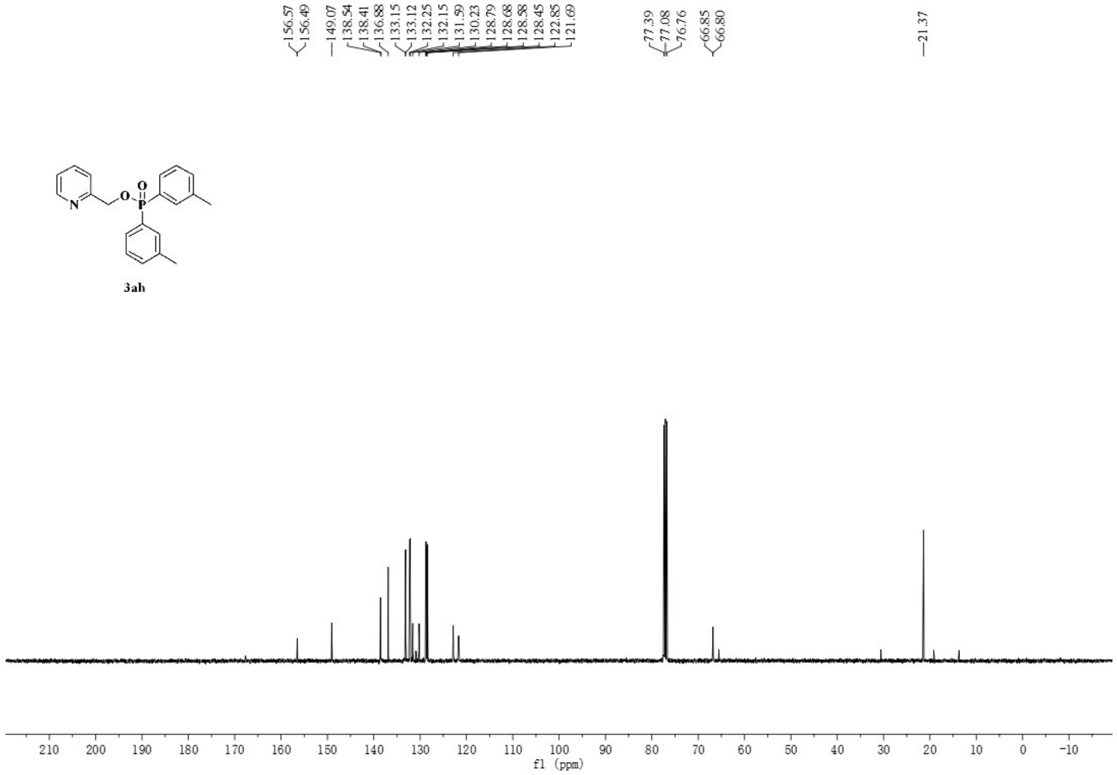

^{31}P NMR of **3ah** (121 MHz, $CDCl_3$)

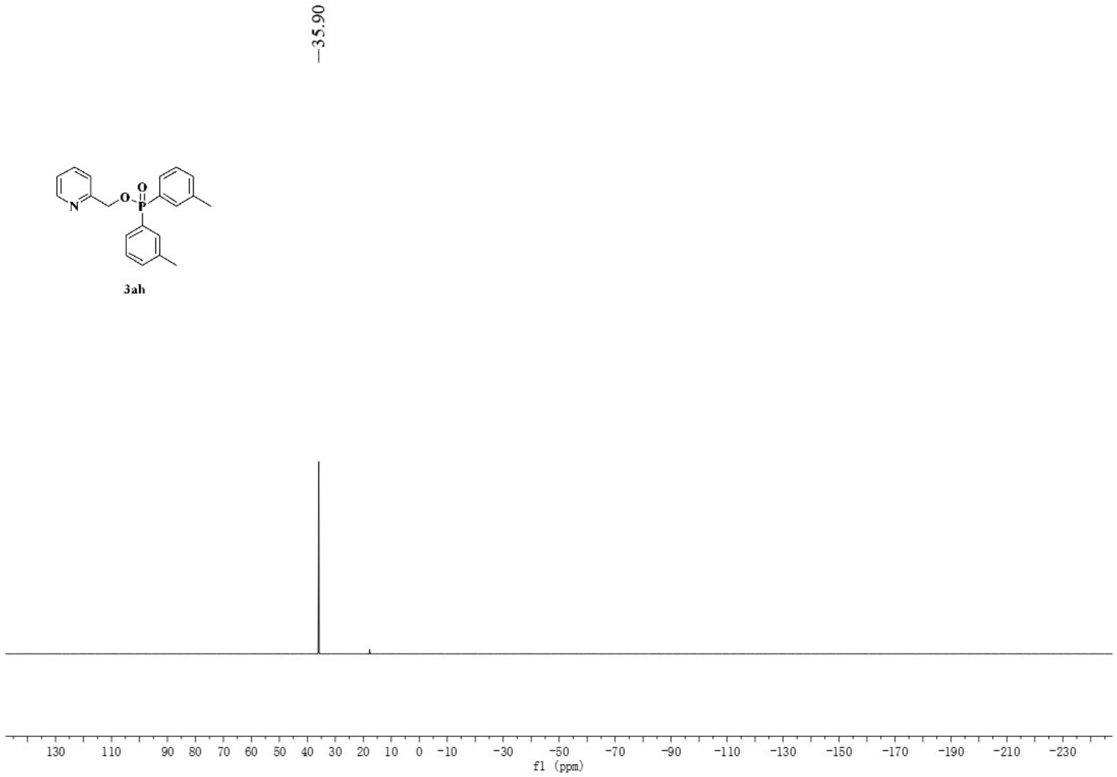

^{1}H NMR of **3ai** (400 MHz, $CDCl_3$)

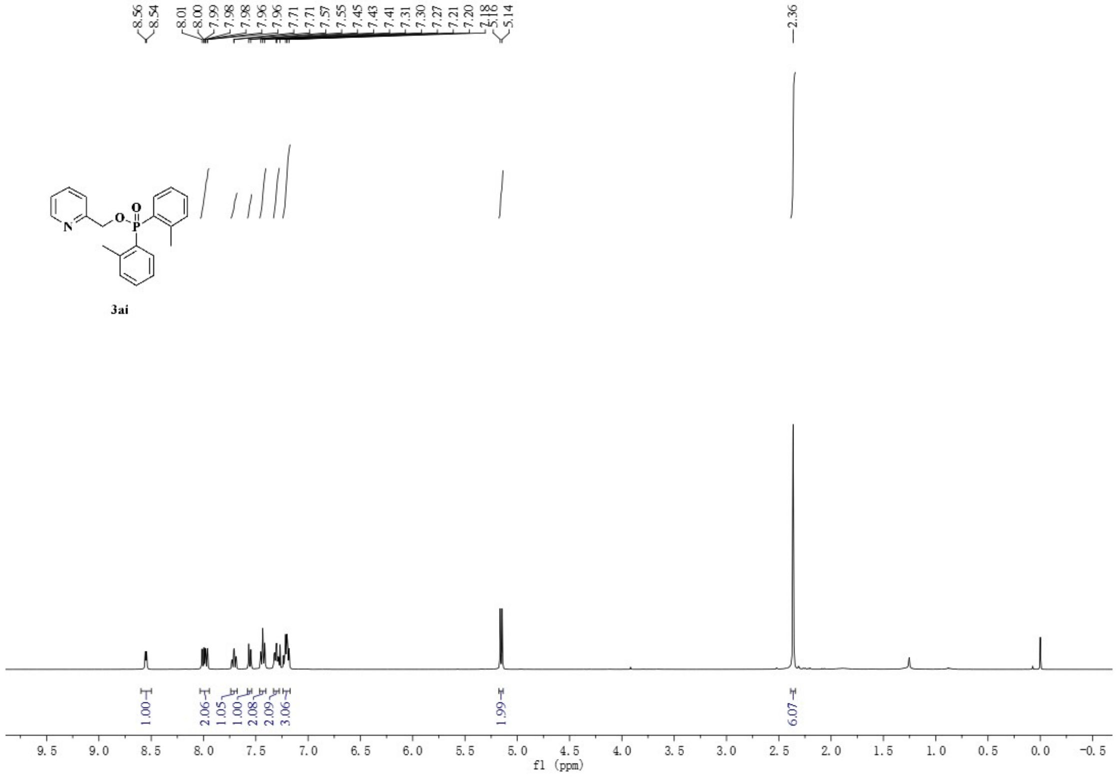

^{13}C NMR of **3ai** (101 MHz, $CDCl_3$)

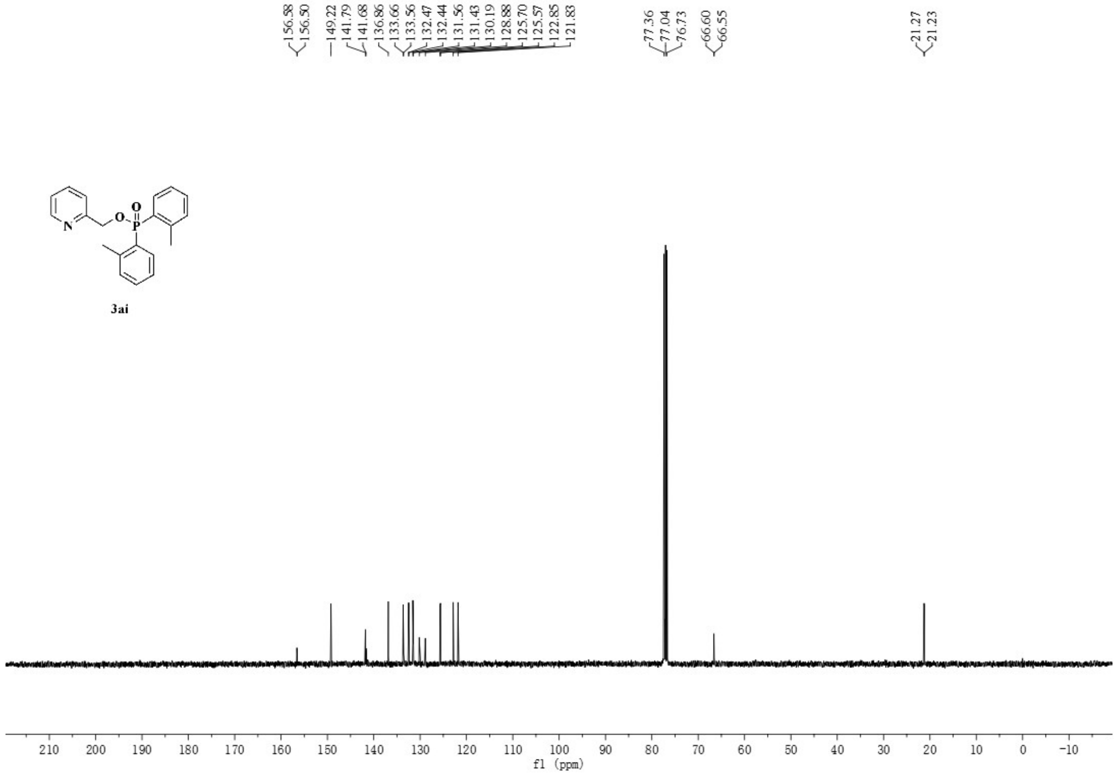

^{31}P NMR of **3ai** (121 MHz, $CDCl_3$)

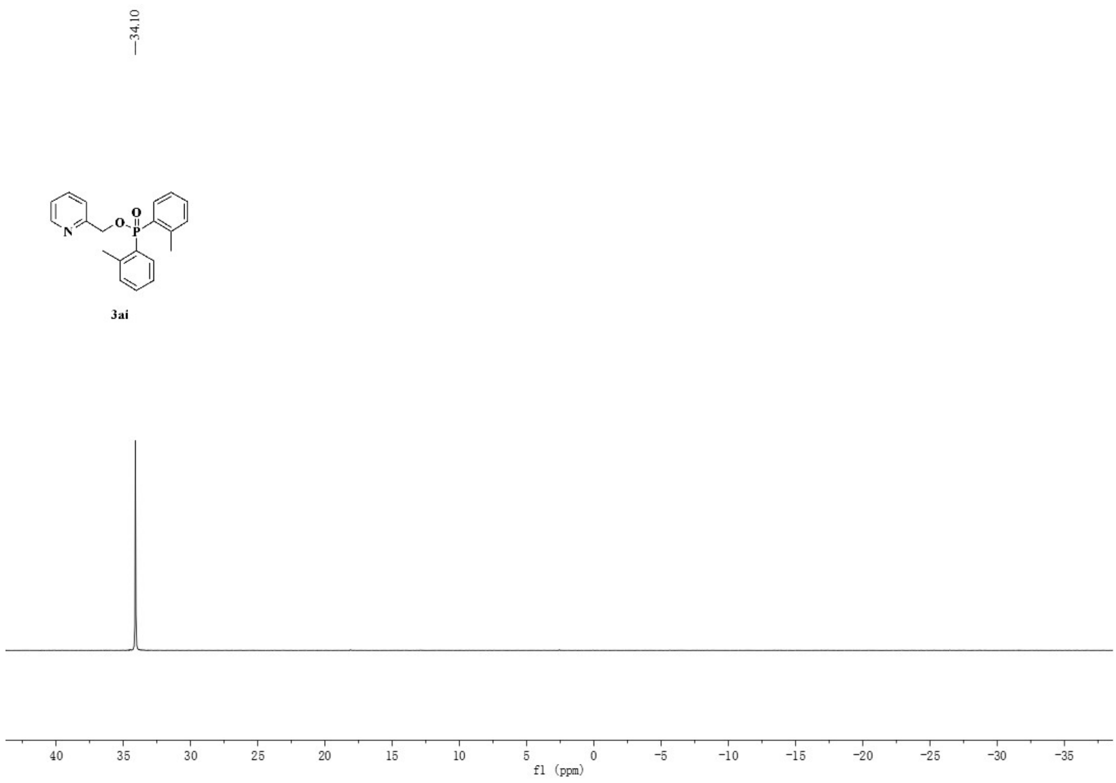

^{1}H NMR of **3aj** (400 MHz, $CDCl_3$)

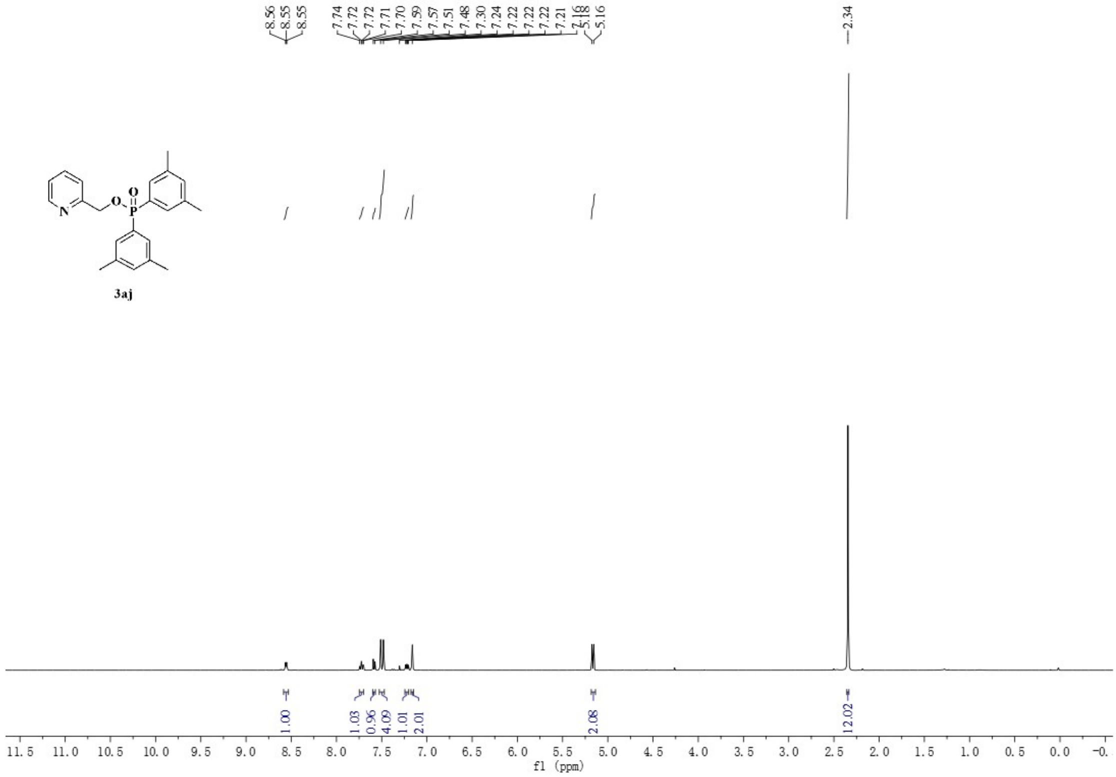

^{13}C NMR of **3aj** (101 MHz, $CDCl_3$)

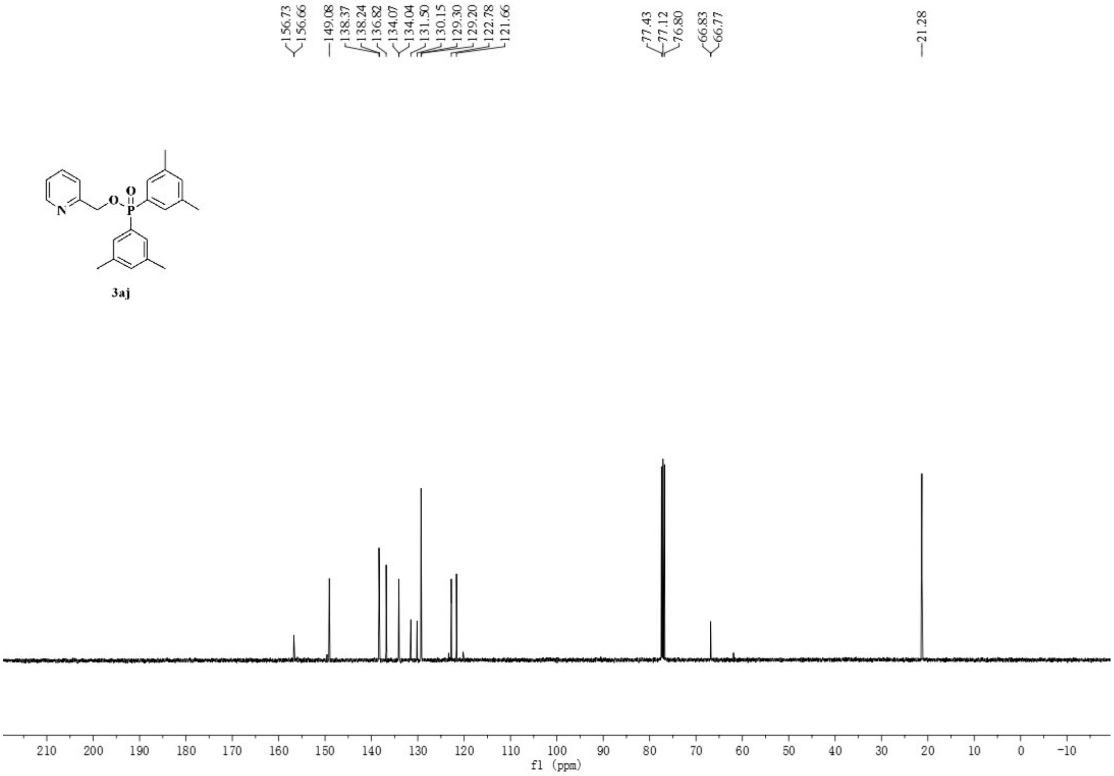

^{31}P NMR of **3aj** (121 MHz, $CDCl_3$)

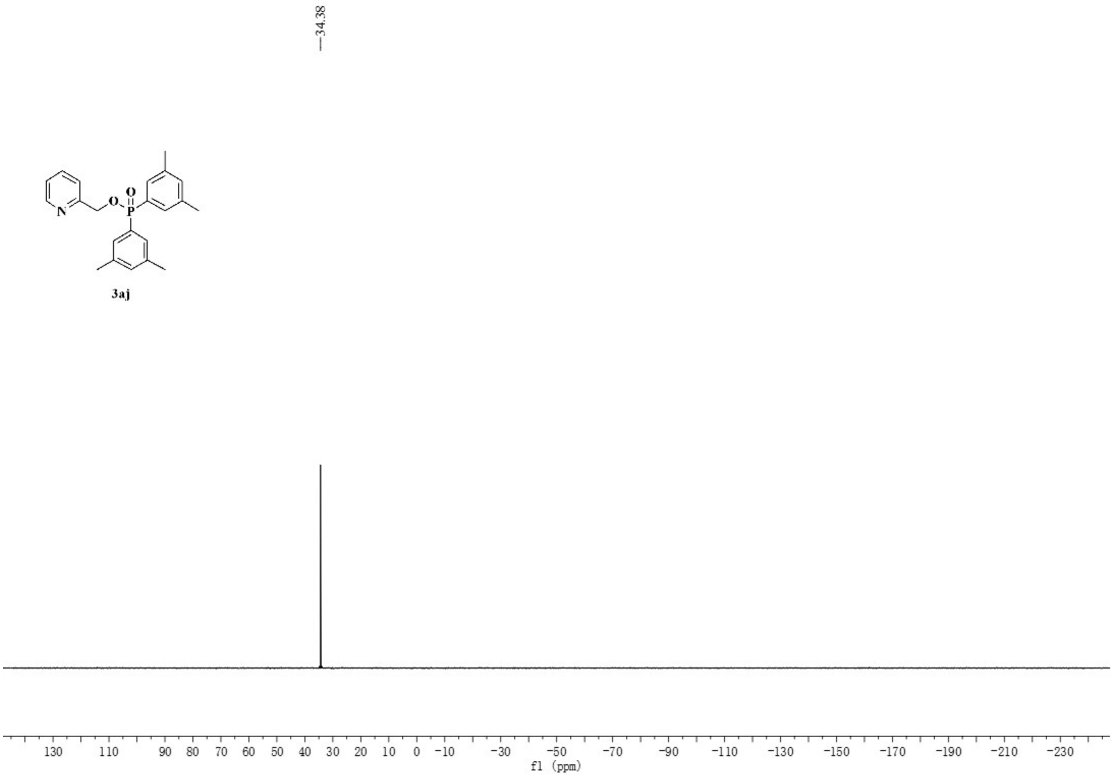

^{1}H NMR of **3ak** (400 MHz, $CDCl_3$)

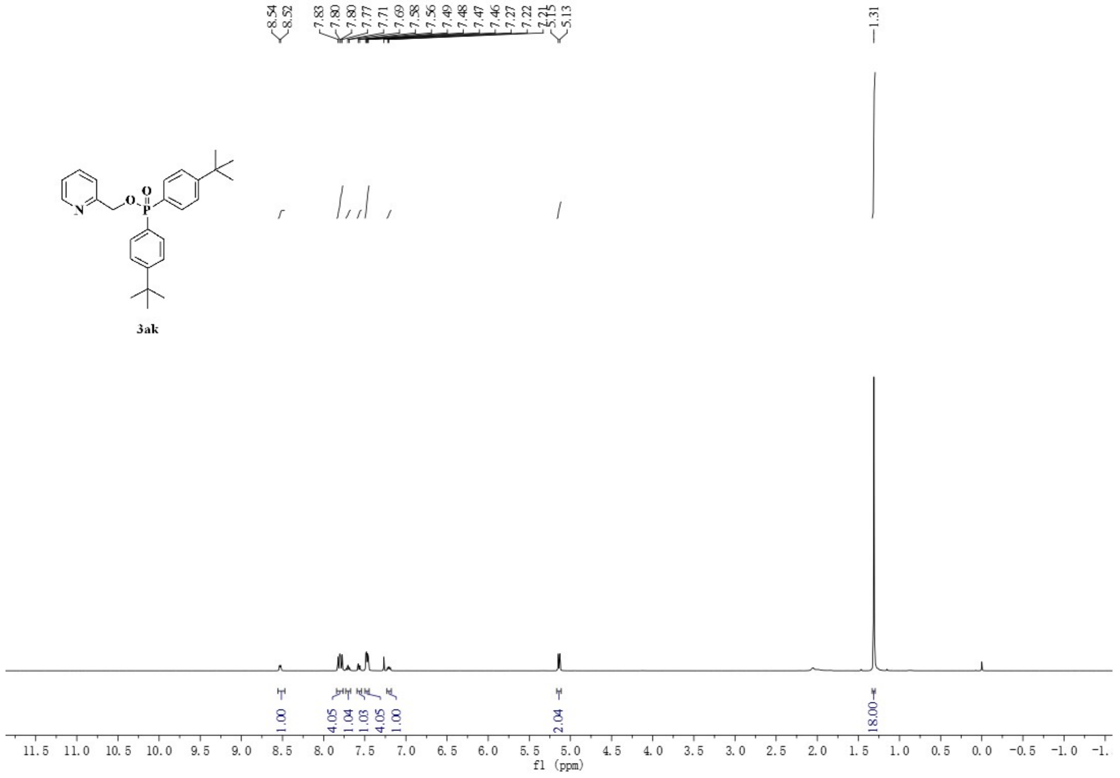

^{13}C NMR of **3ak** (101 MHz, $CDCl_3$)

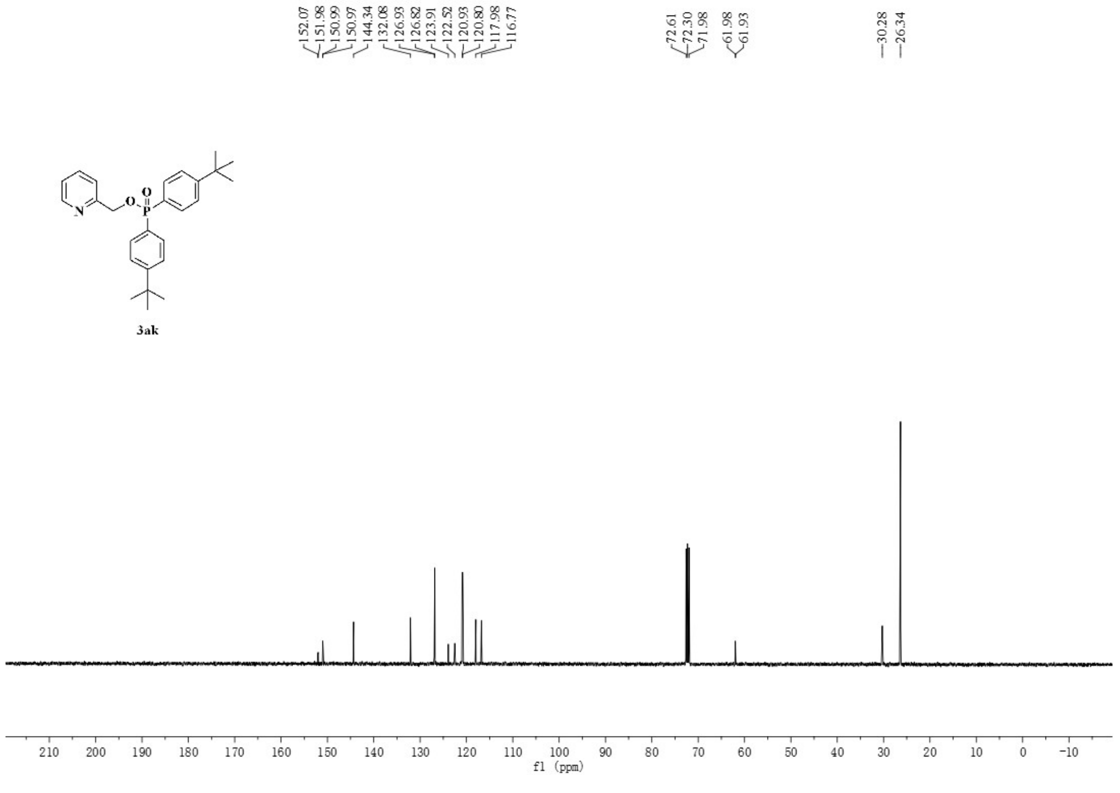

^{31}P NMR of **3ak** (121 MHz, $CDCl_3$)

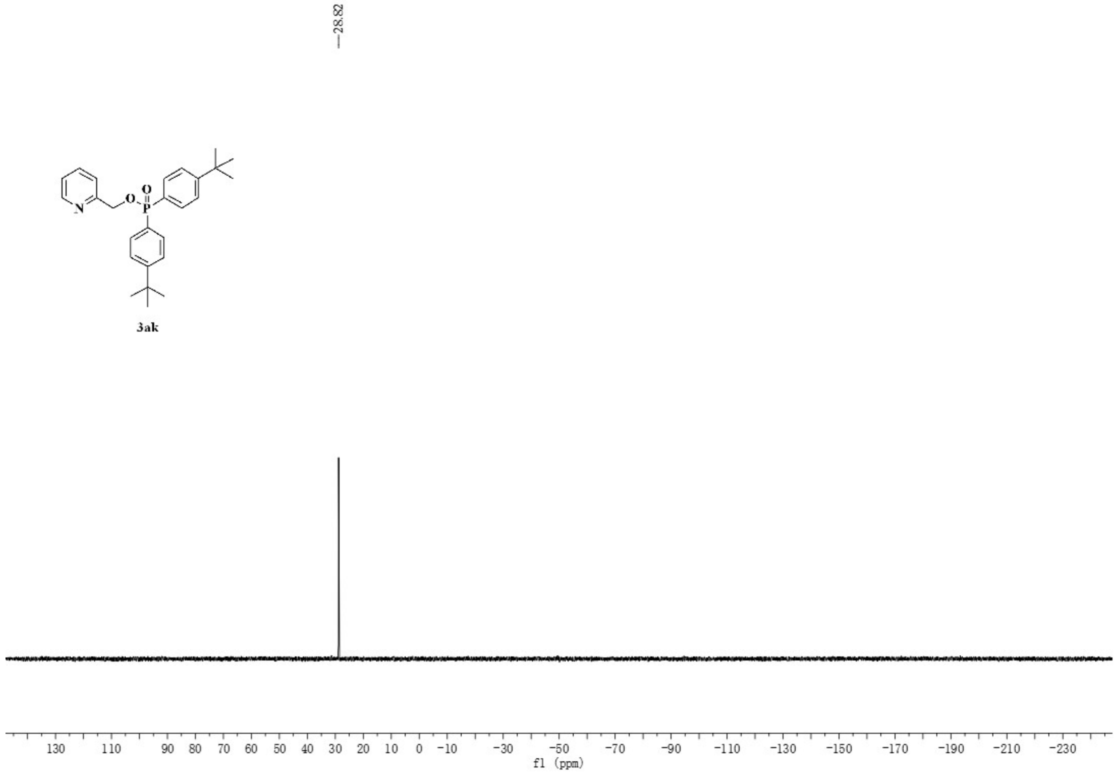

^{1}H NMR of **3al** (400 MHz, $CDCl_3$)

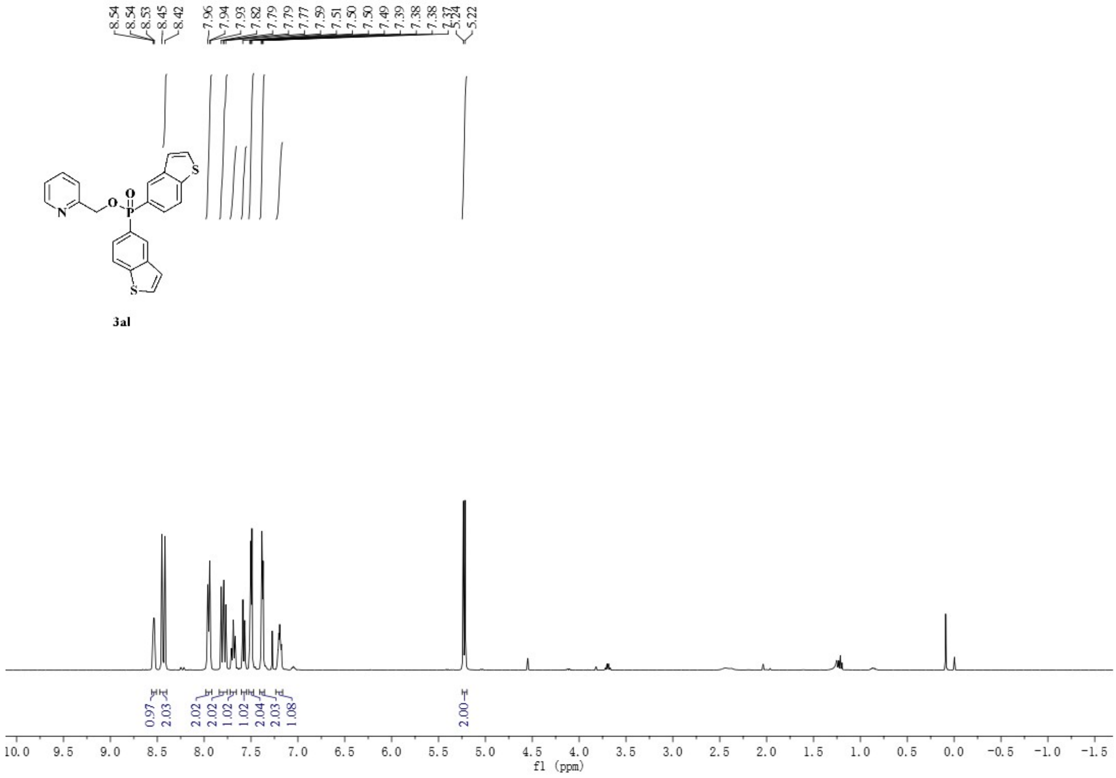

^{13}C NMR of **3al** (101 MHz, $CDCl_3$)

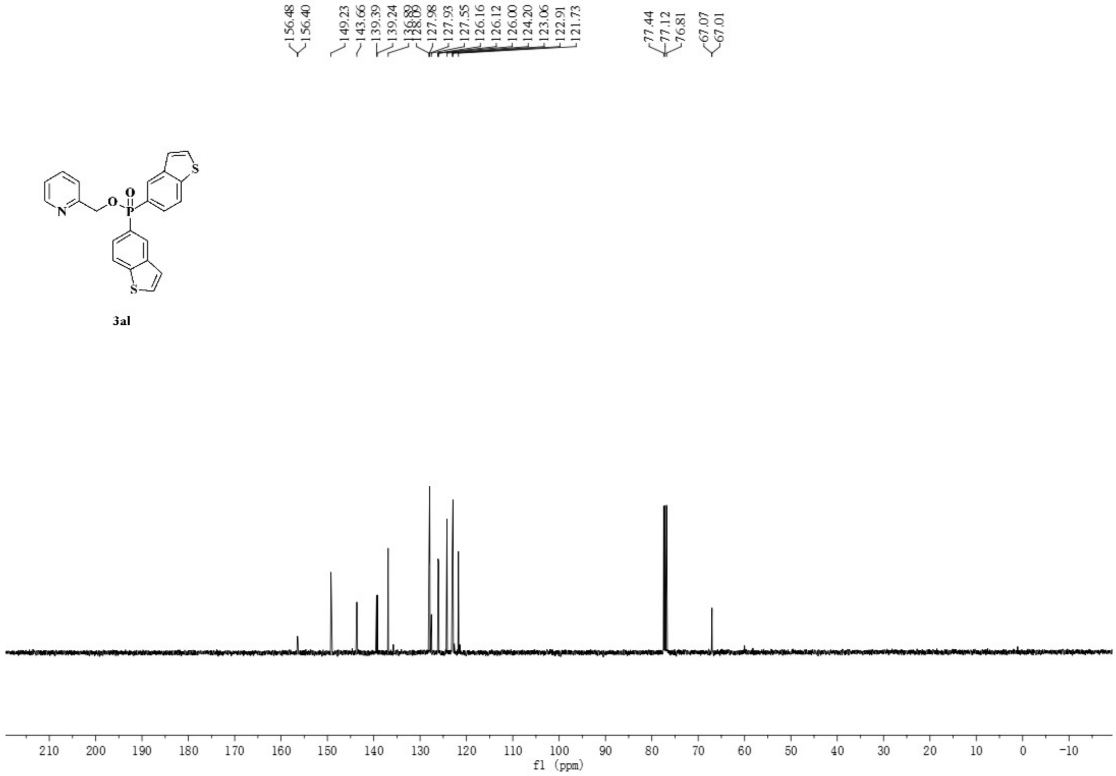

^{31}P NMR of **3al** (121 MHz, $CDCl_3$)

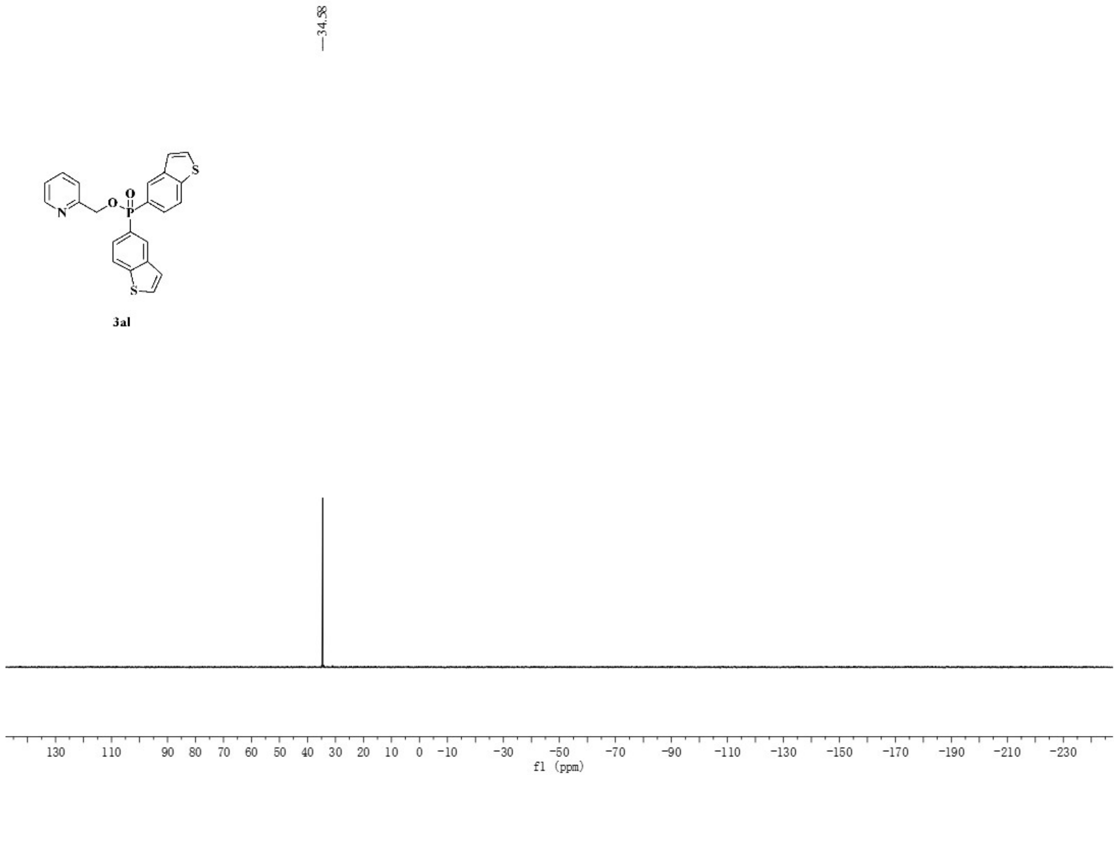

^{1}H NMR of **3am** (400 MHz, $CDCl_3$)

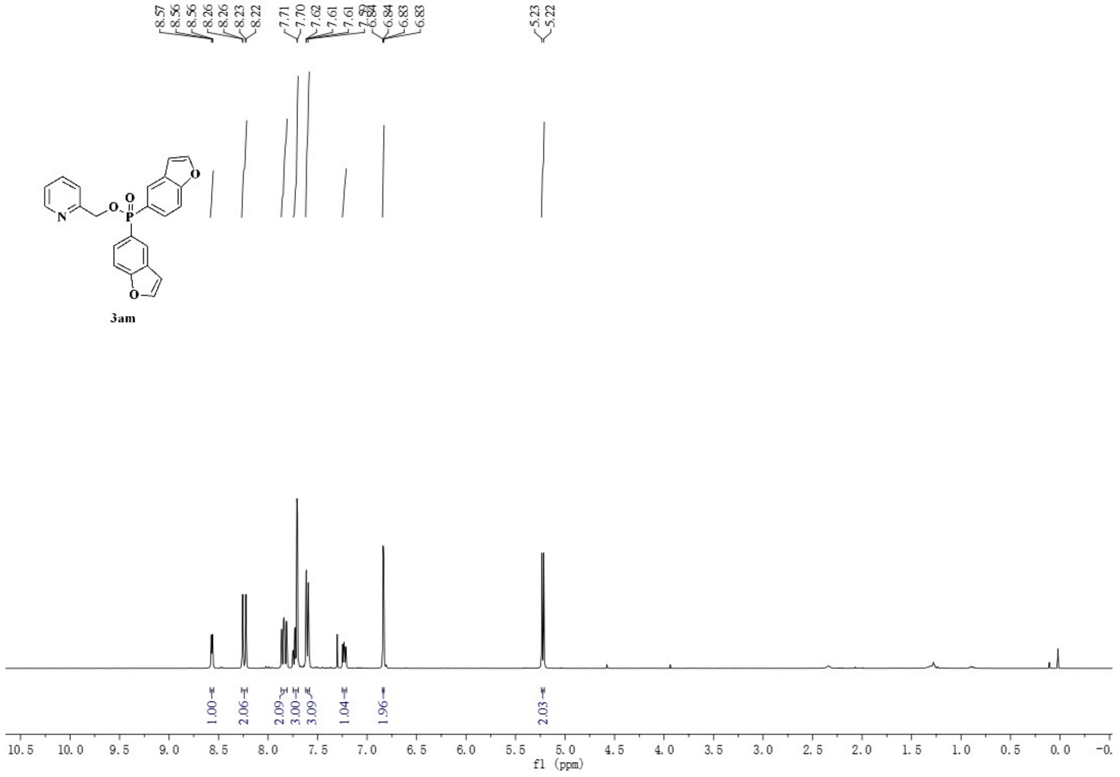

^{13}C NMR of **3am** (101 MHz, $CDCl_3$)

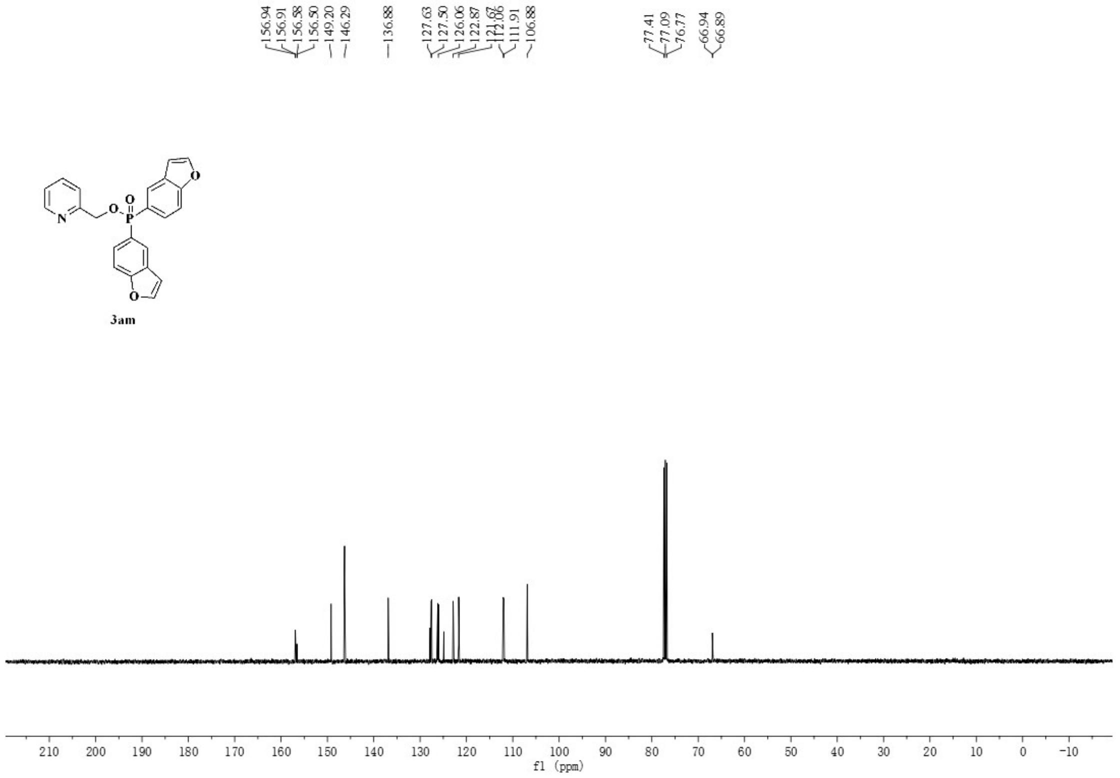

^{31}P NMR of **3am** (121 MHz, $CDCl_3$)

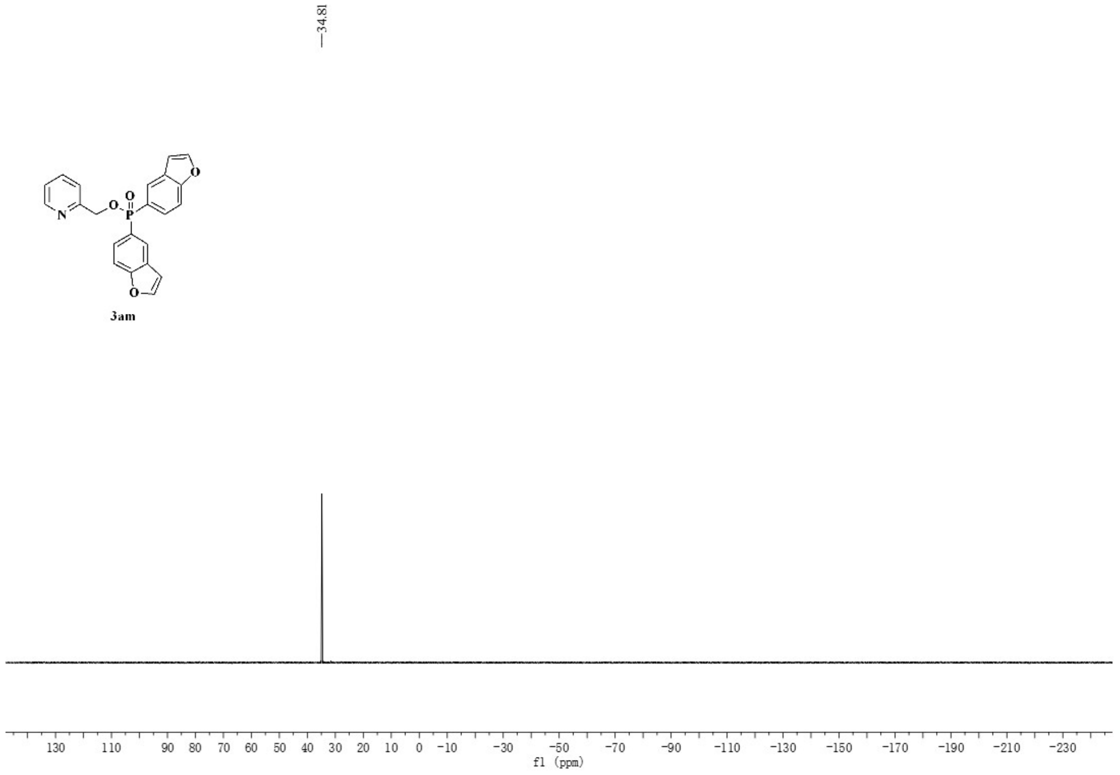

^{1}H NMR of **3an** (400 MHz, $CDCl_3$)

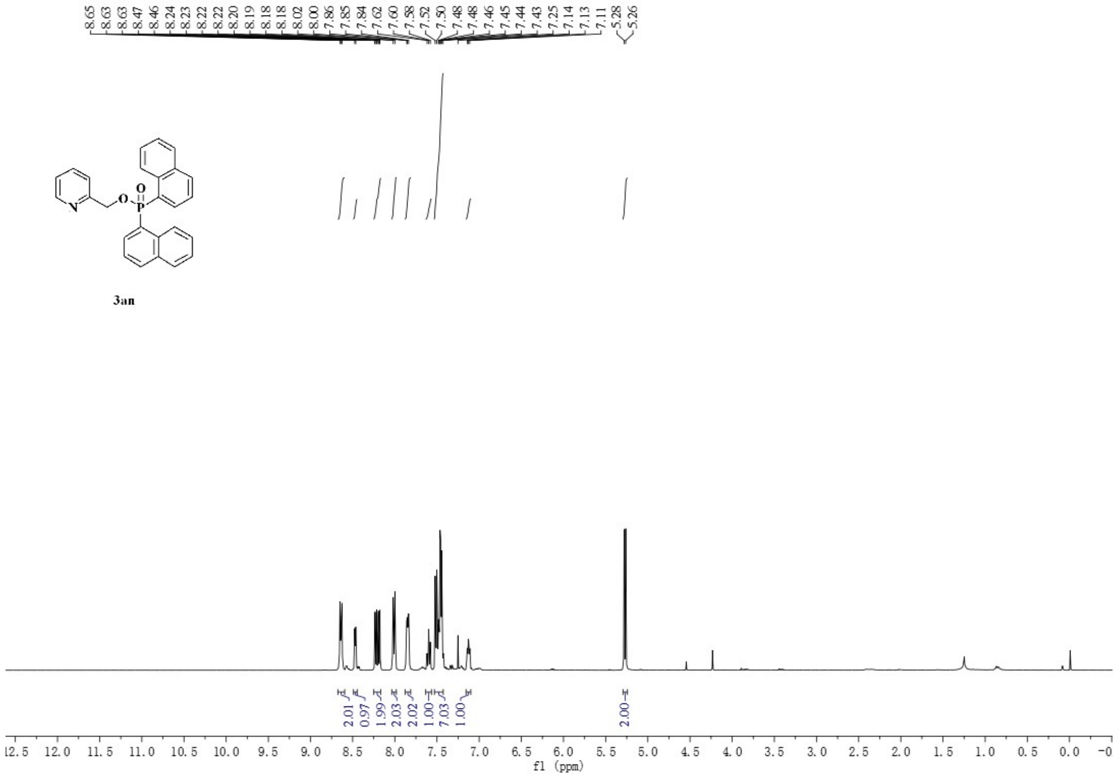

^{13}C NMR of **3an** (101 MHz, $CDCl_3$)

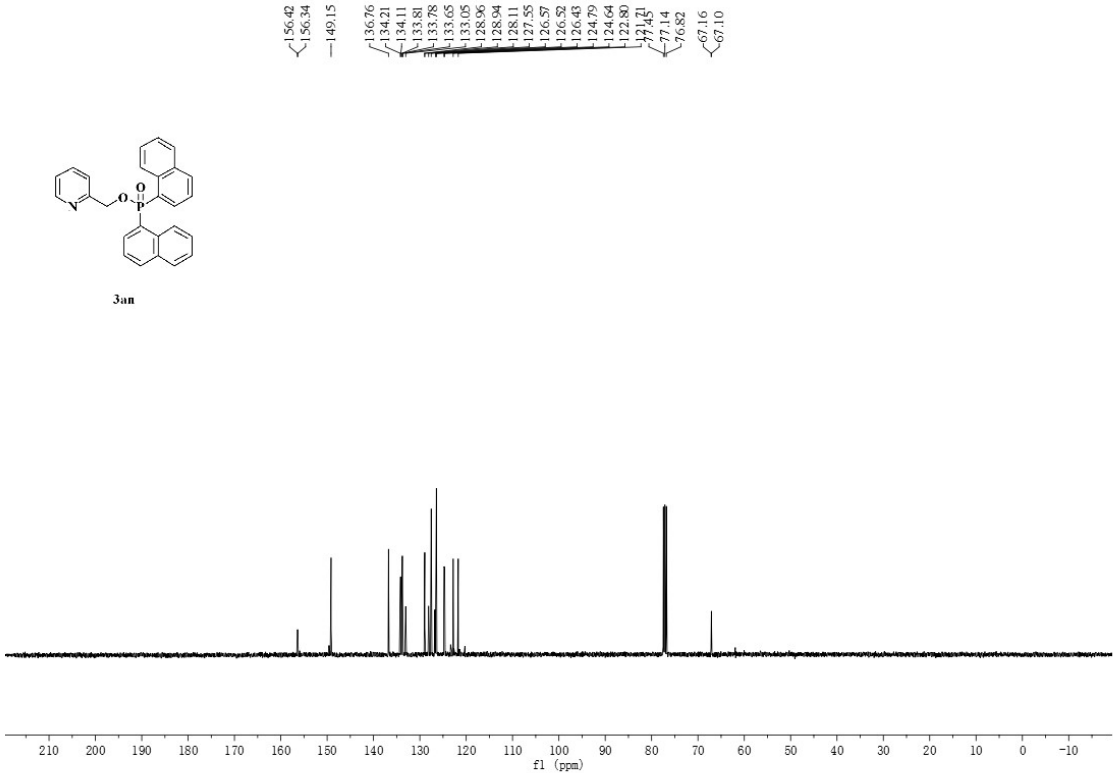

^{31}P NMR of **3an** (121 MHz, $CDCl_3$)

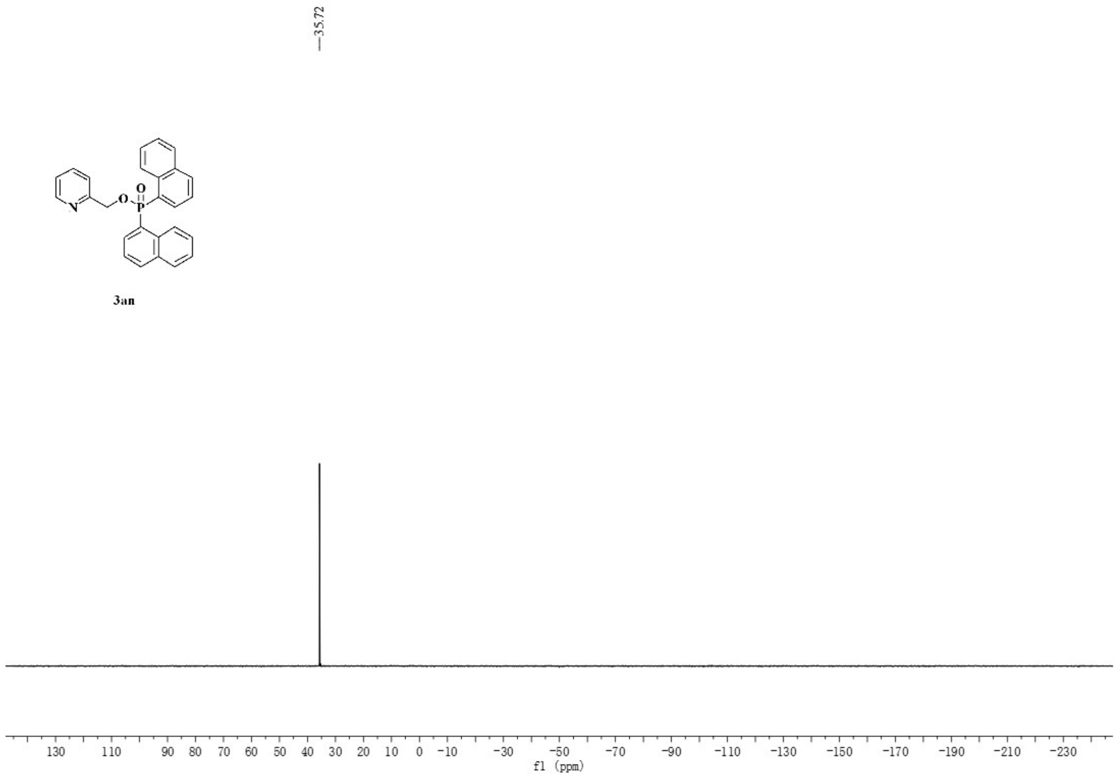

^{1}H NMR of **3ao** (400 MHz, $CDCl_3$)

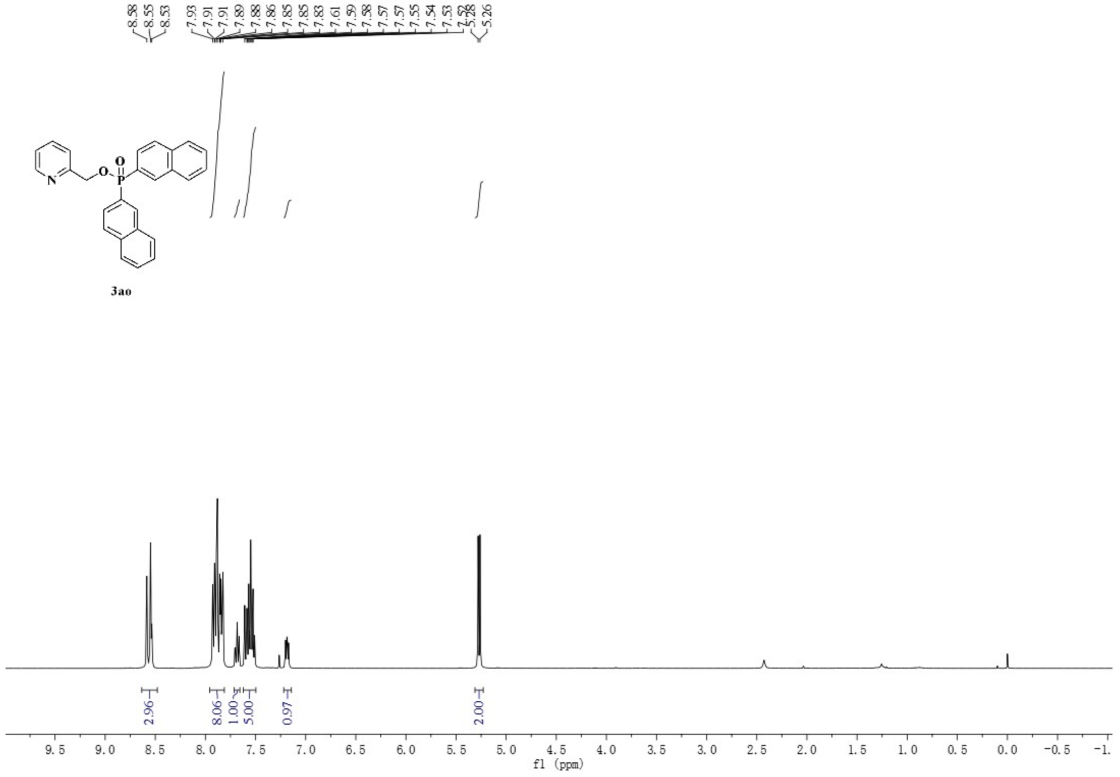

^{13}C NMR of **3ao** (101 MHz, $CDCl_3$)

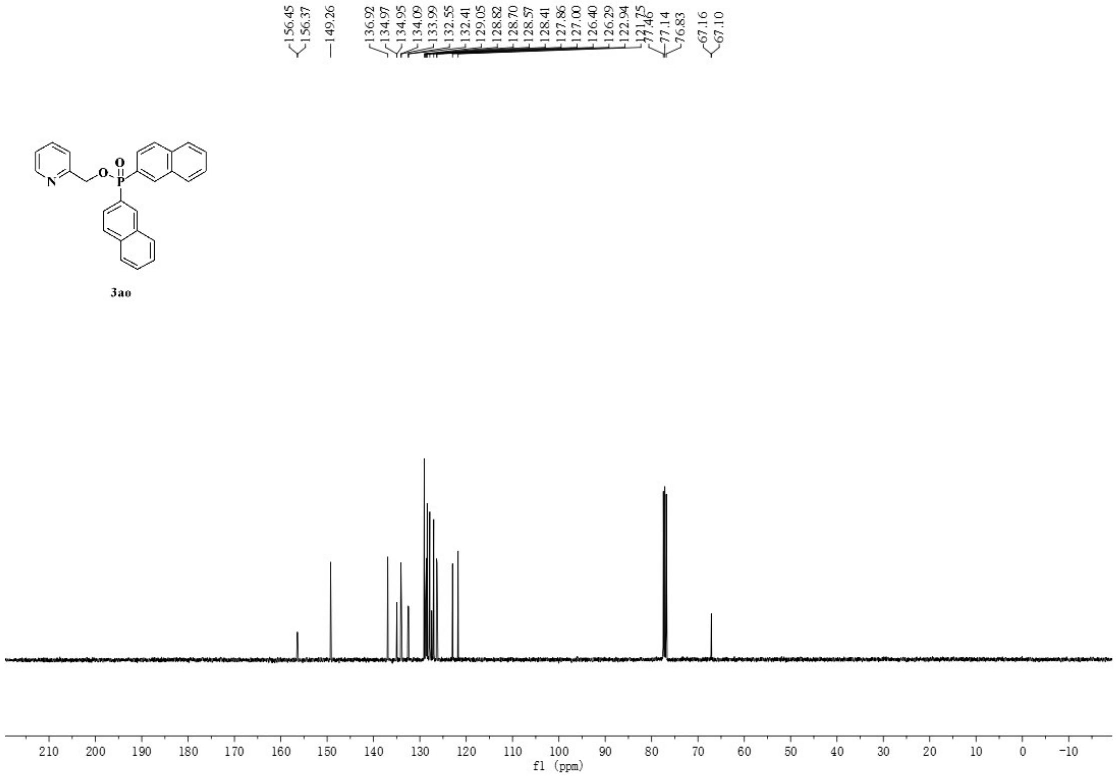

^{31}P NMR of **3ao** (121 MHz, $CDCl_3$)

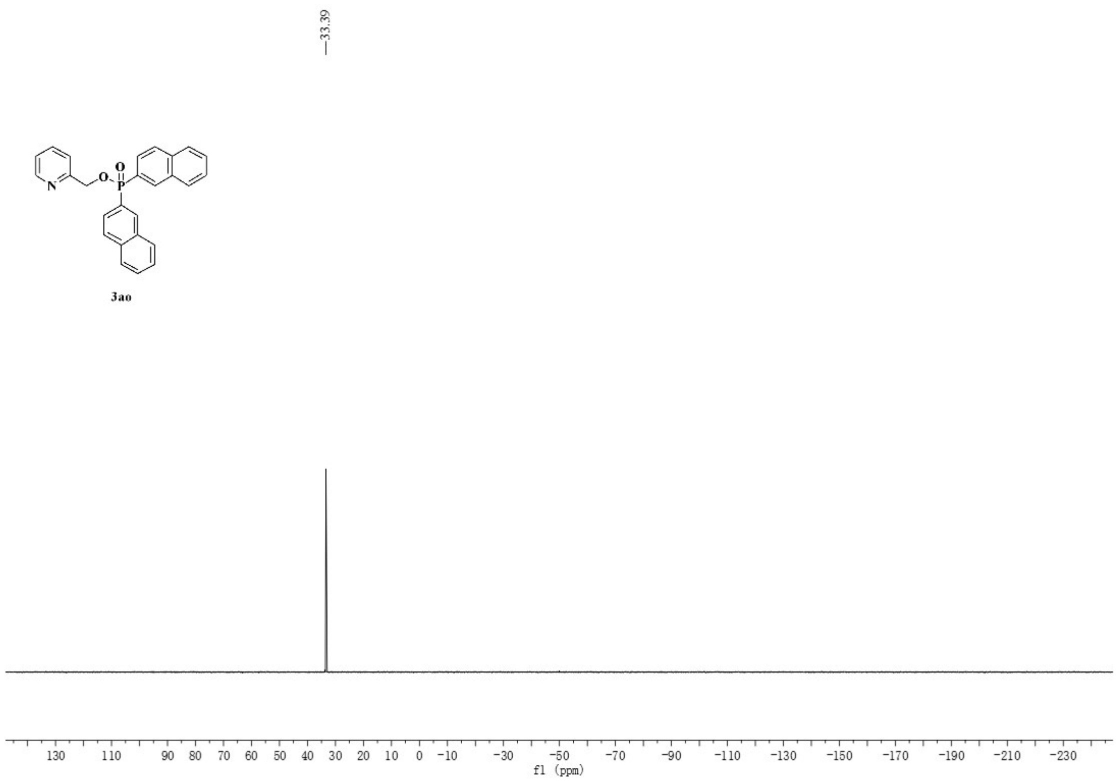

^{1}H NMR of **3ap** (400 MHz, $CDCl_3$)

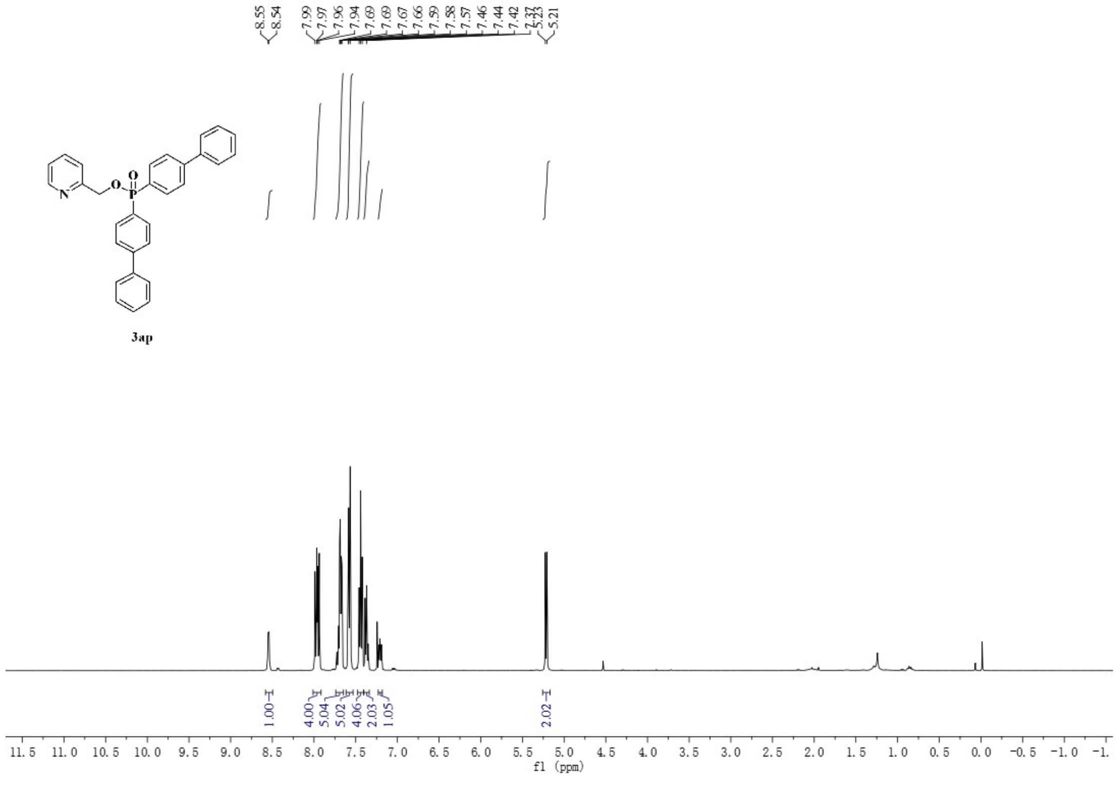

^{13}C NMR of **3ap** (101 MHz, $CDCl_3$)

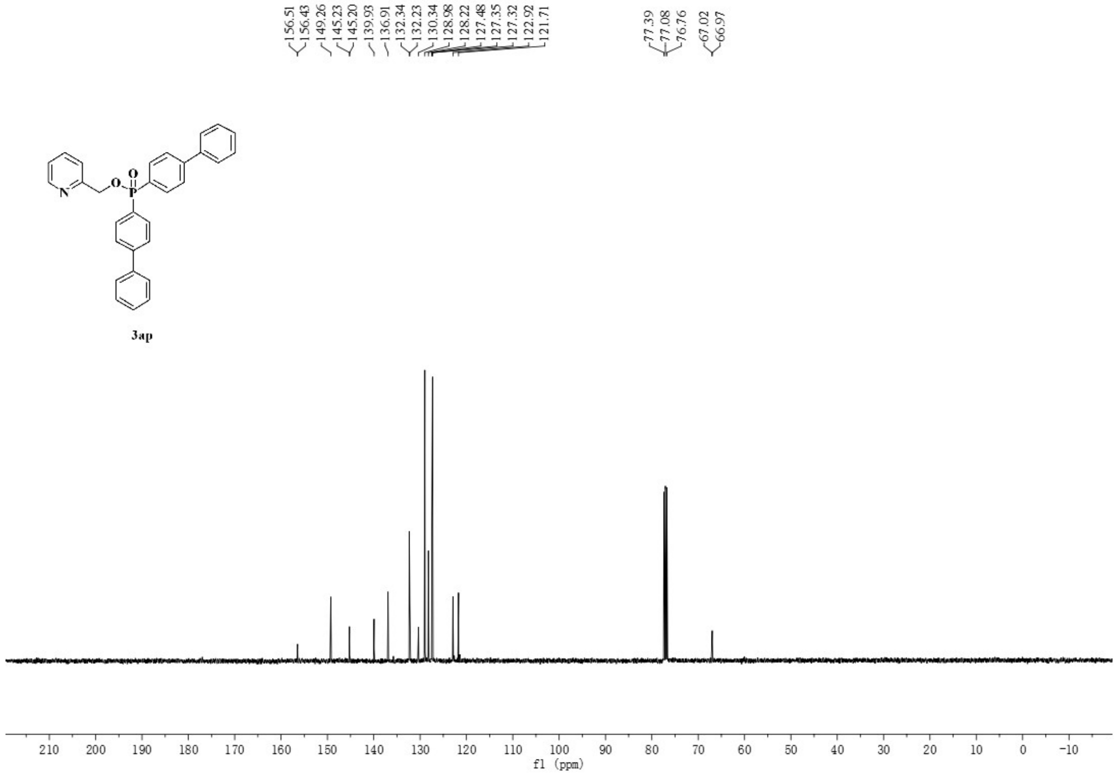

^{31}P NMR of **3ap** (121 MHz, $CDCl_3$)

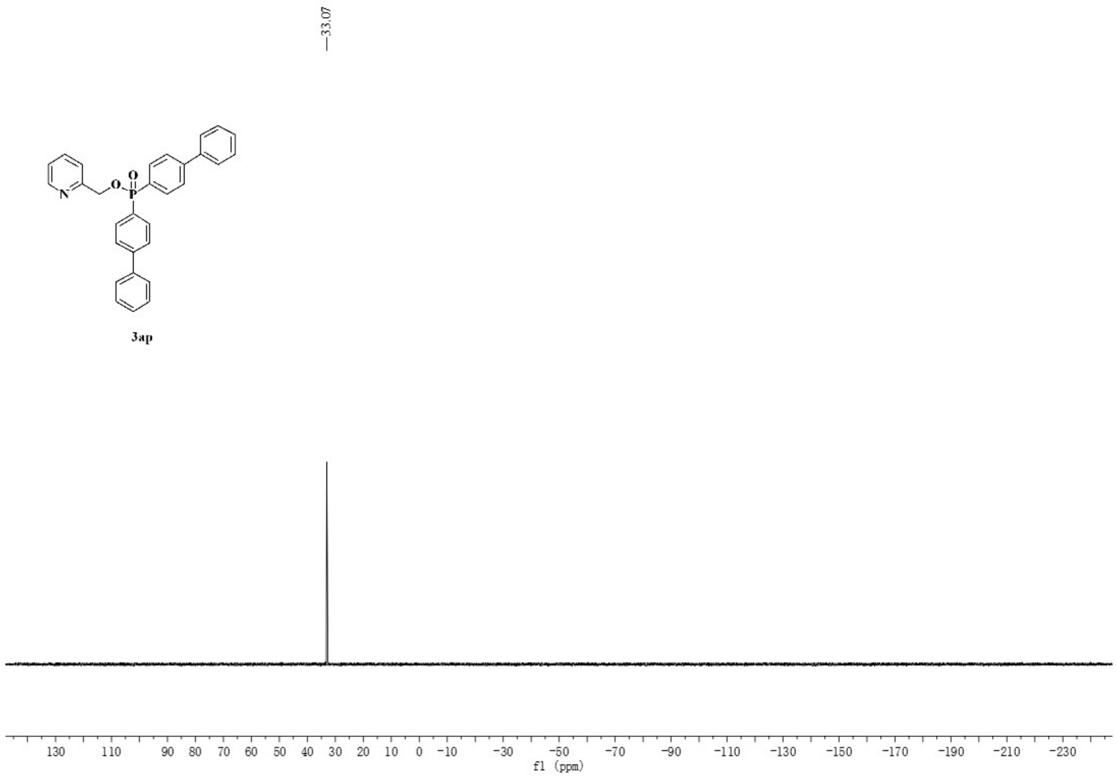

^{1}H NMR of **3aq** (400 MHz, $CDCl_3$)

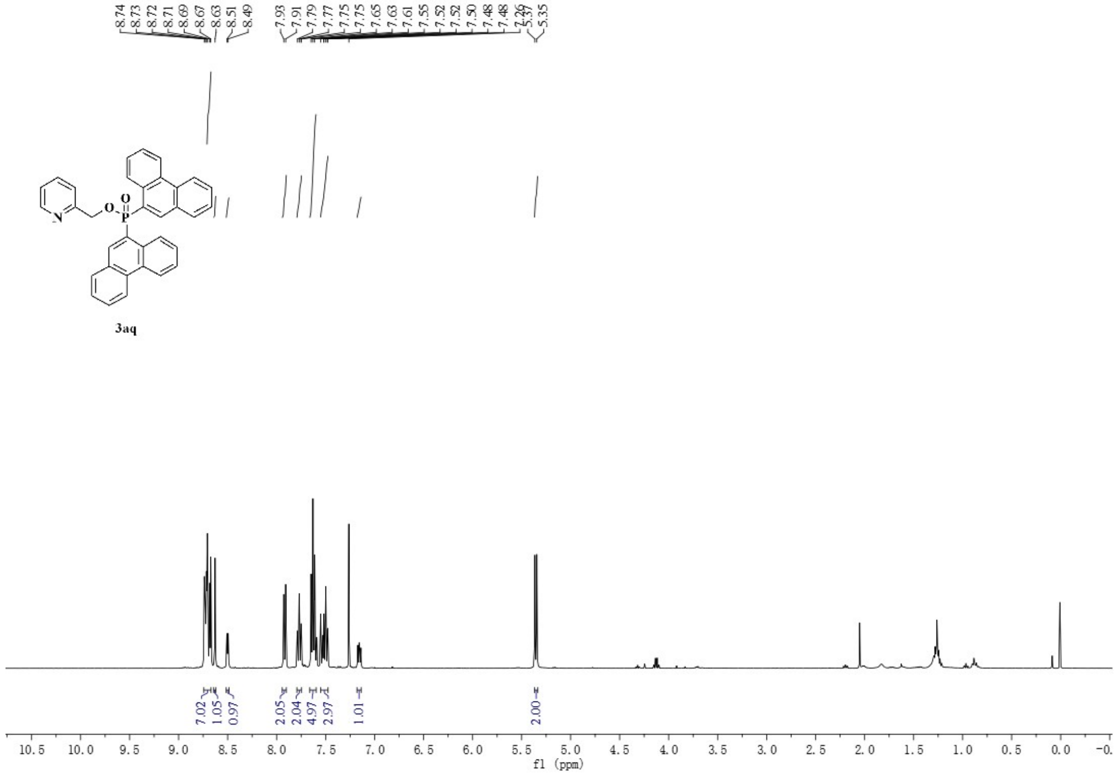

^{13}C NMR of **3aq** (101 MHz, $CDCl_3$)

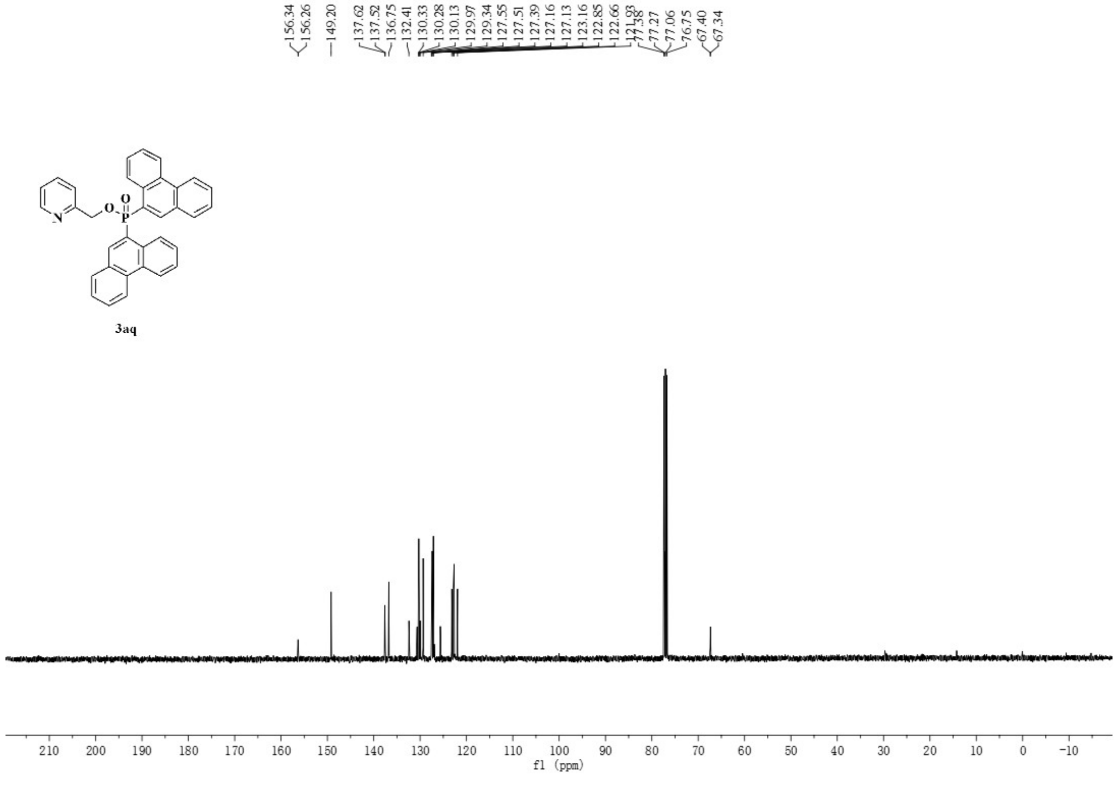

^{31}P NMR of **3aq** (121 MHz, $CDCl_3$)

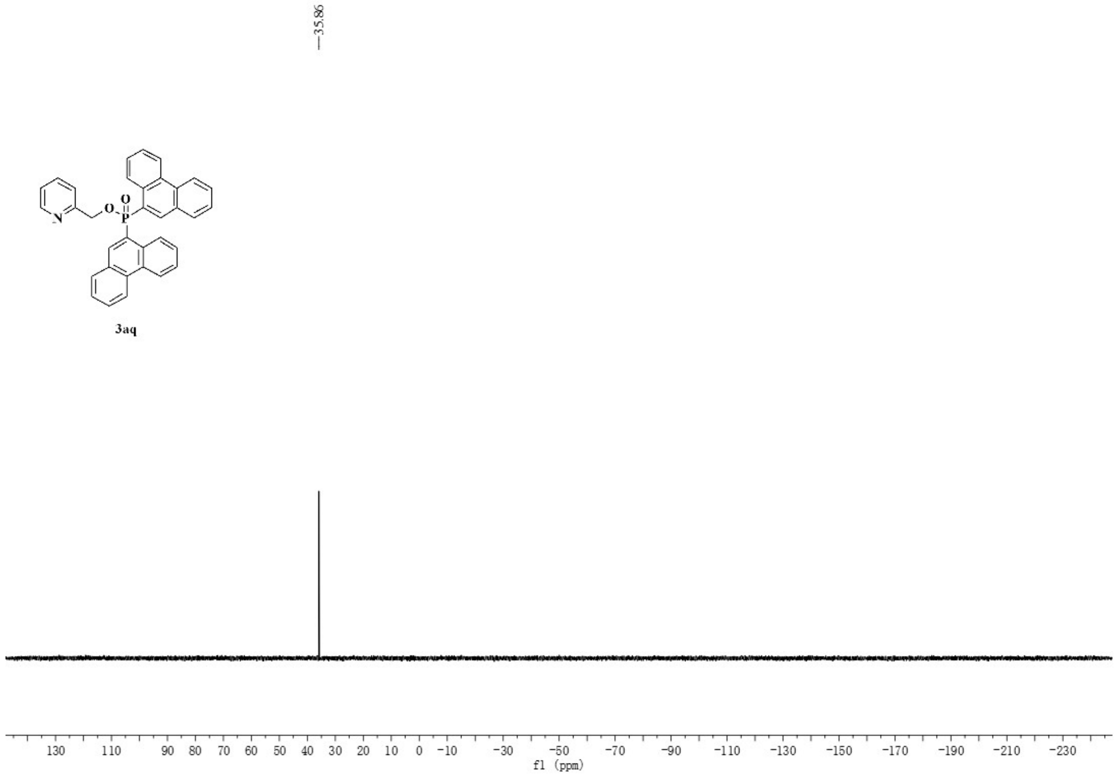

^{1}H NMR of **3ar** (400 MHz, $CDCl_3$)

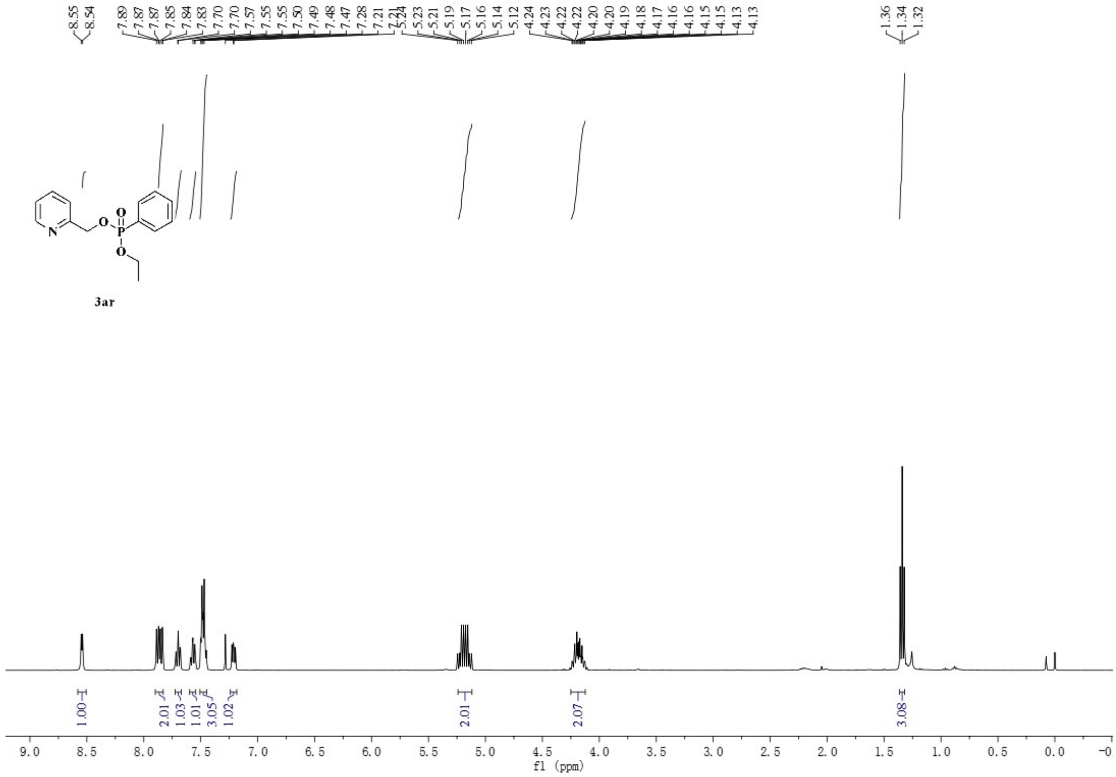

^{13}C NMR of **3ar** (101 MHz, $CDCl_3$)

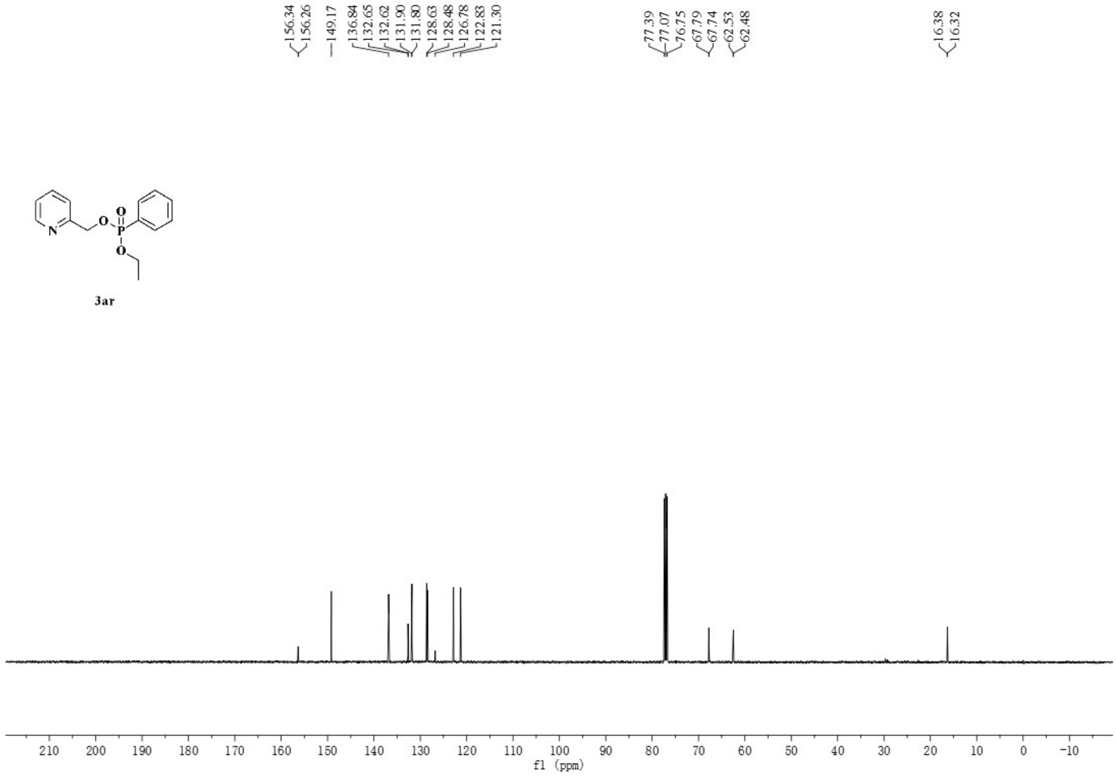

^{31}P NMR of **3ar** (121 MHz, $CDCl_3$)

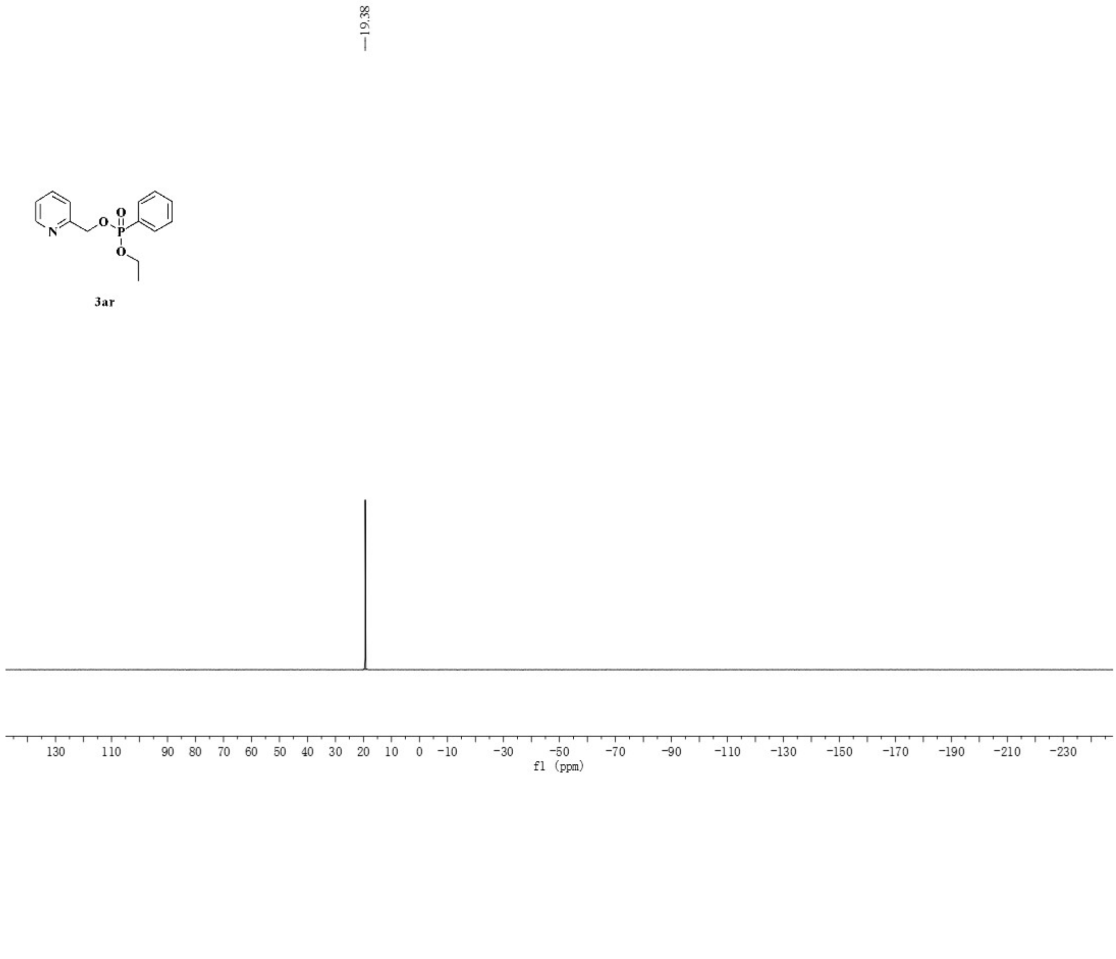

^{1}H NMR of **3as** (400 MHz, $CDCl_3$)

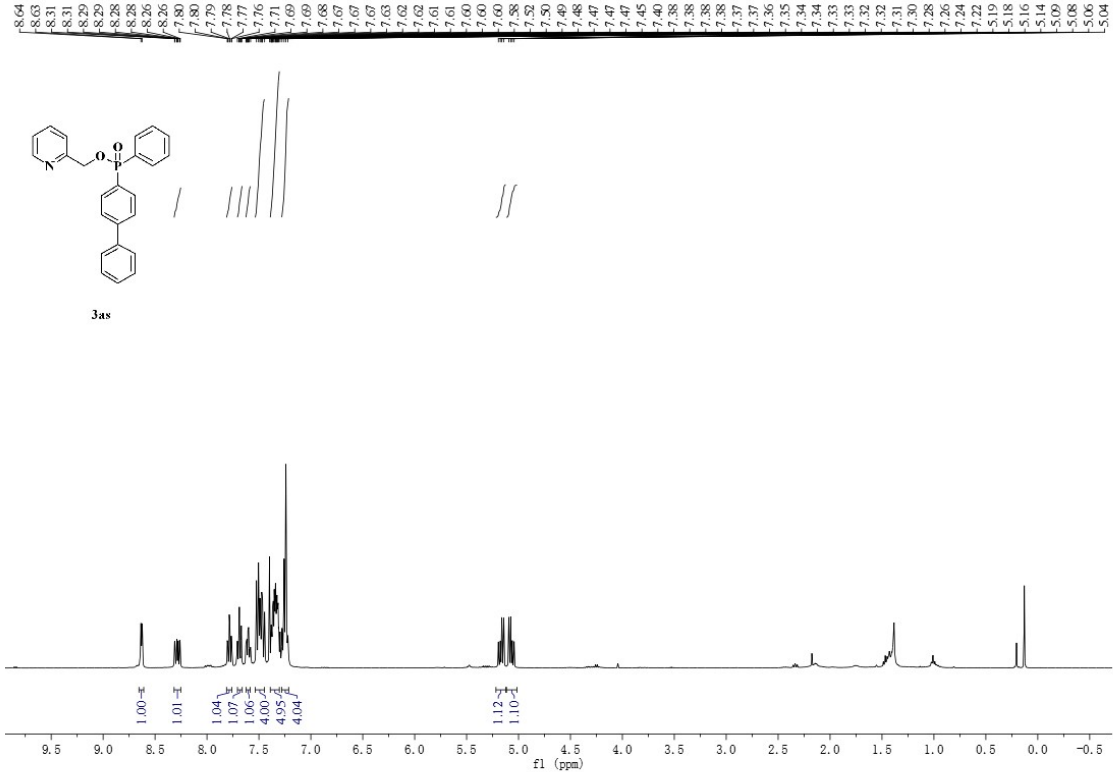

^{13}C NMR of **3as** (101 MHz, $CDCl_3$)

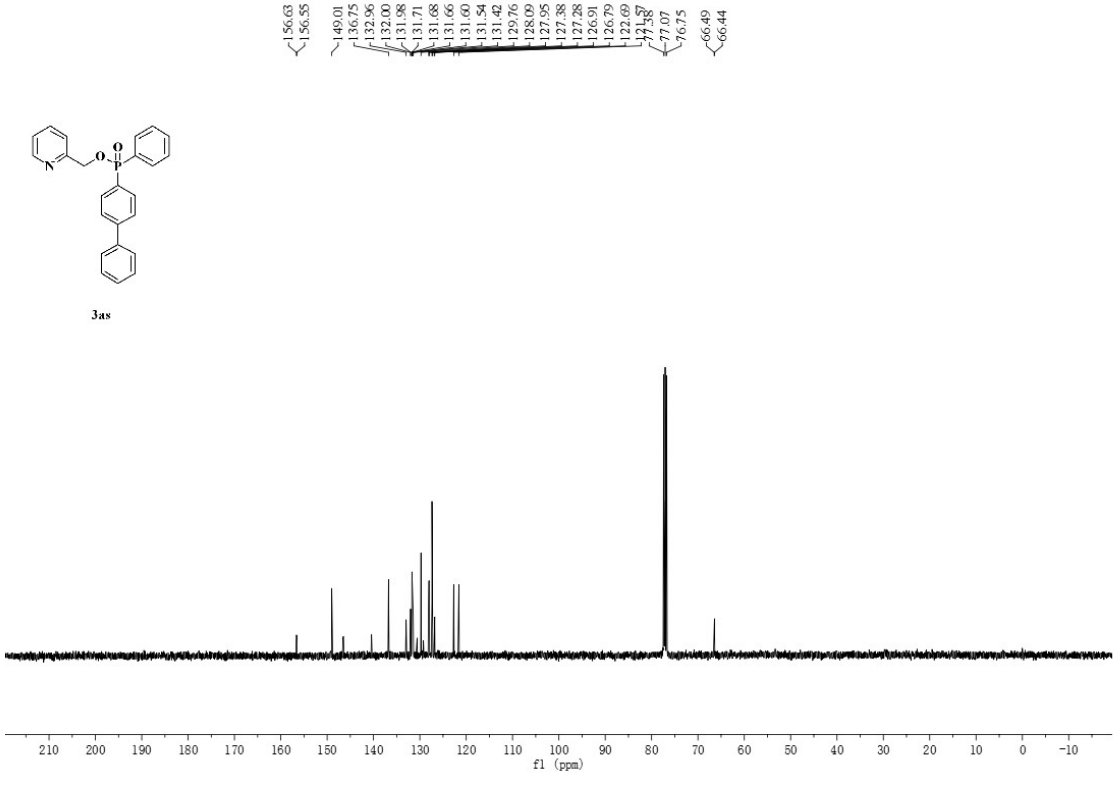

^{31}P NMR of **3as** (121 MHz, $CDCl_3$)

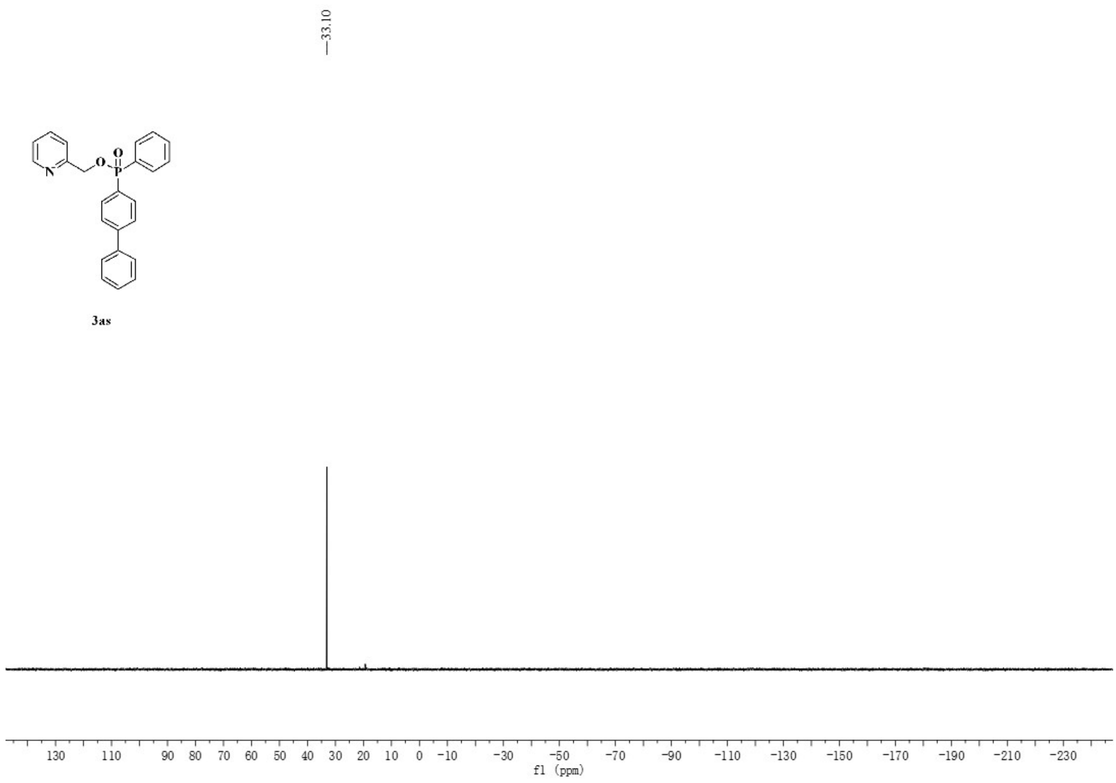

^{1}H NMR of **3at** (400 MHz, $CDCl_3$)

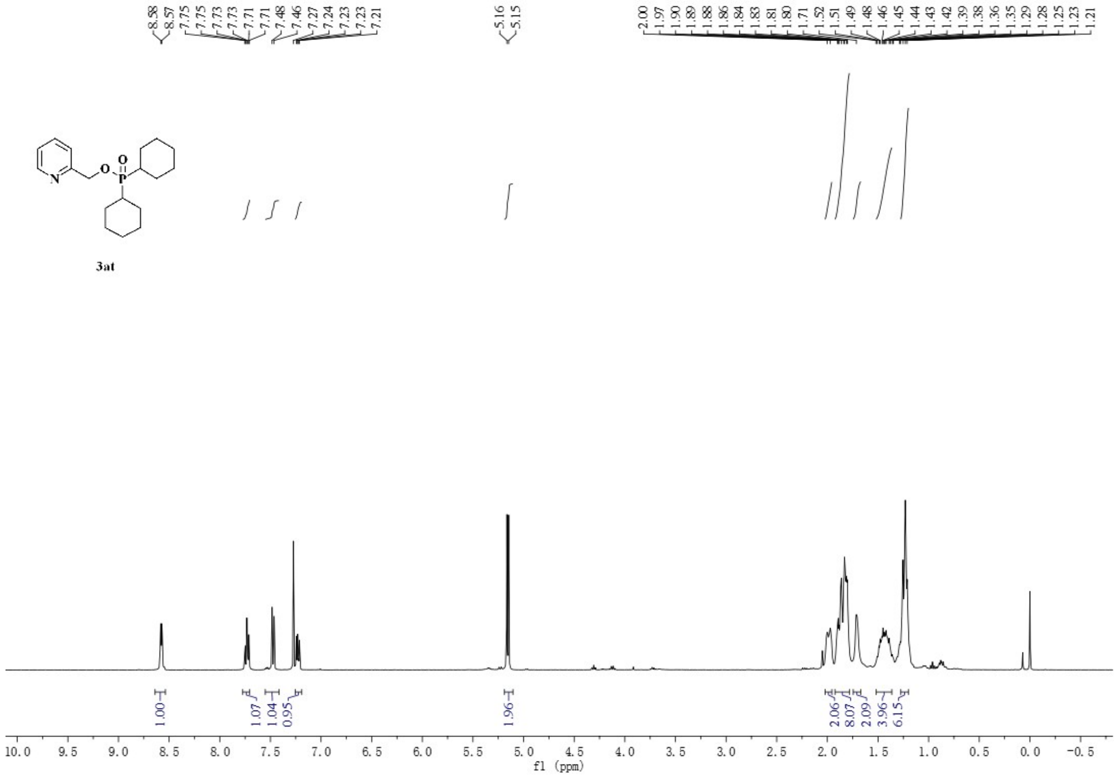

^{13}C NMR of **3at** (101 MHz, $CDCl_3$)

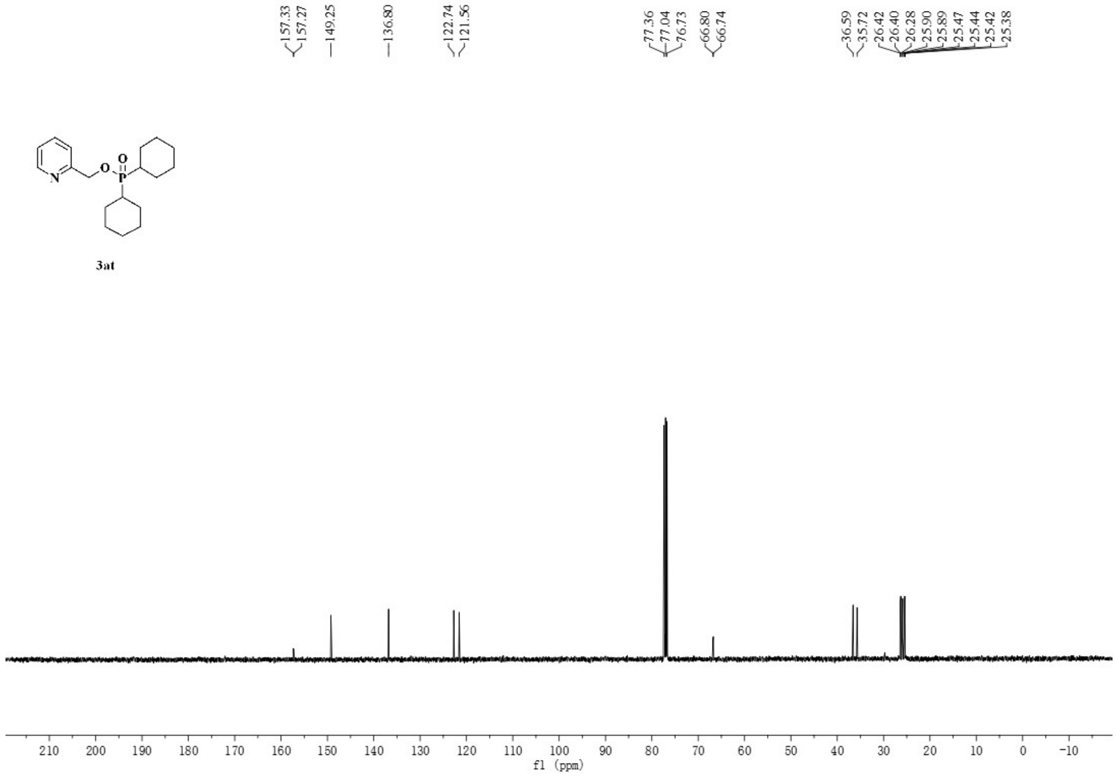

^{31}P NMR of **3at** (121 MHz, $CDCl_3$)

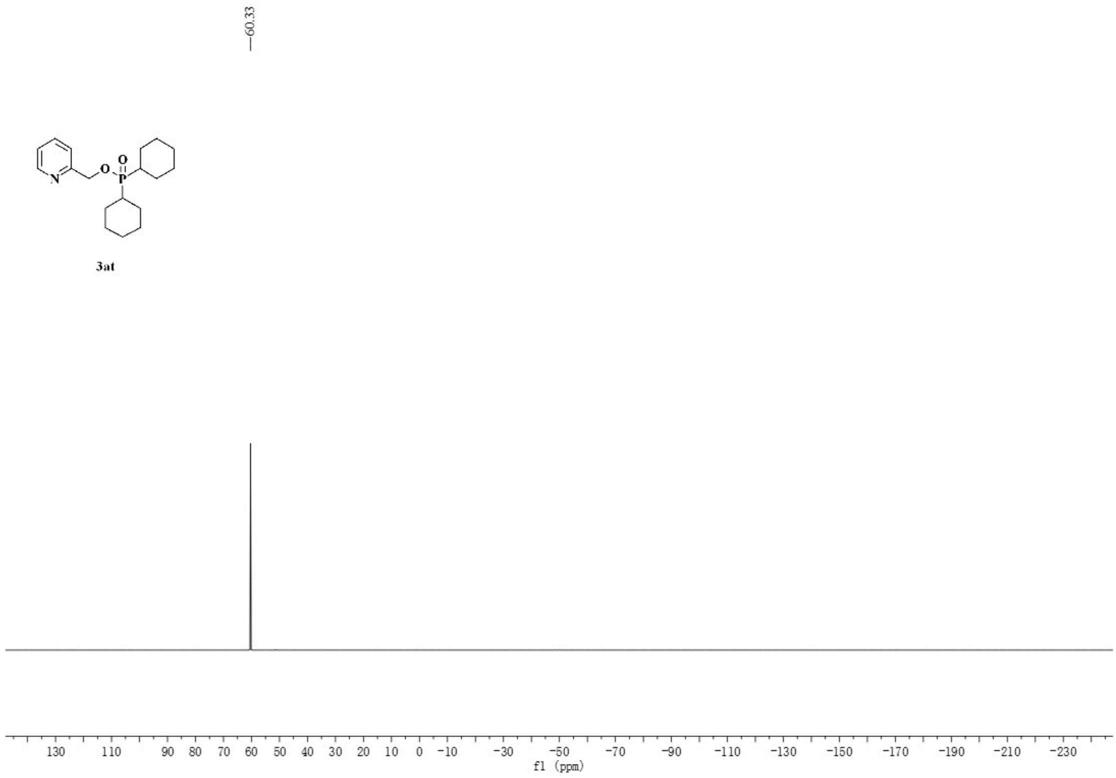

^{1}H NMR of **3au** (400 MHz, $CDCl_3$)

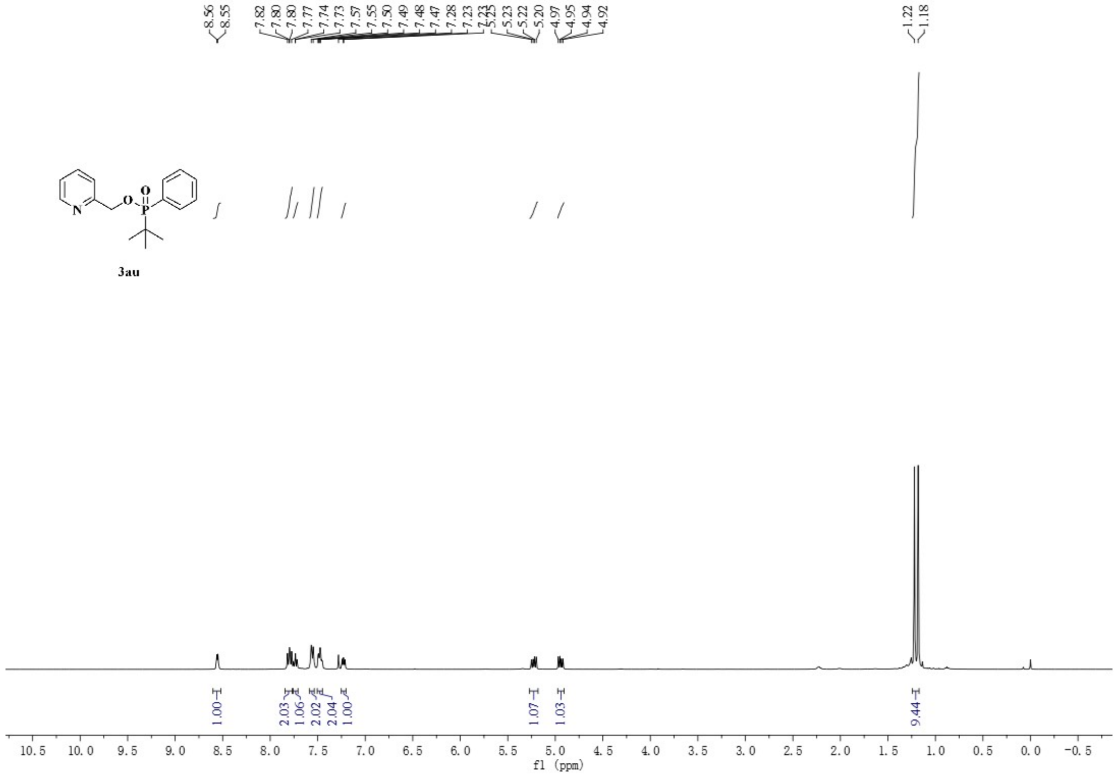

^{13}C NMR of **3at** (101 MHz, $CDCl_3$)

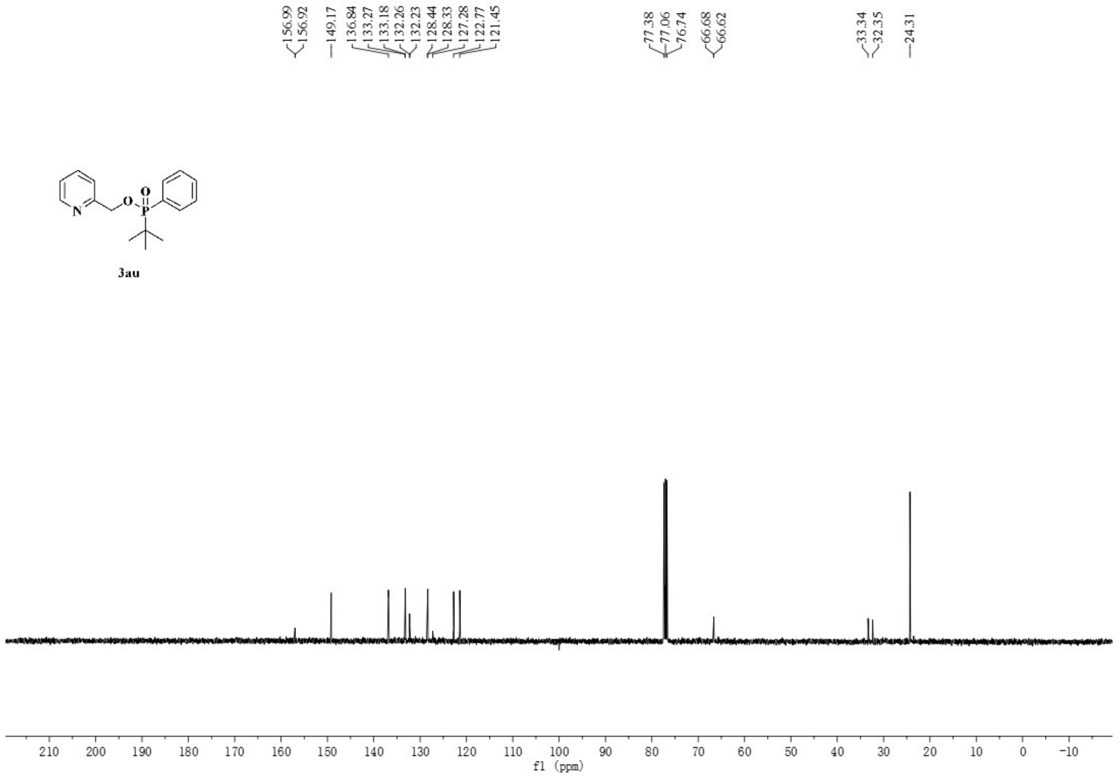

^{31}P NMR of **3au** (121 MHz, $CDCl_3$)

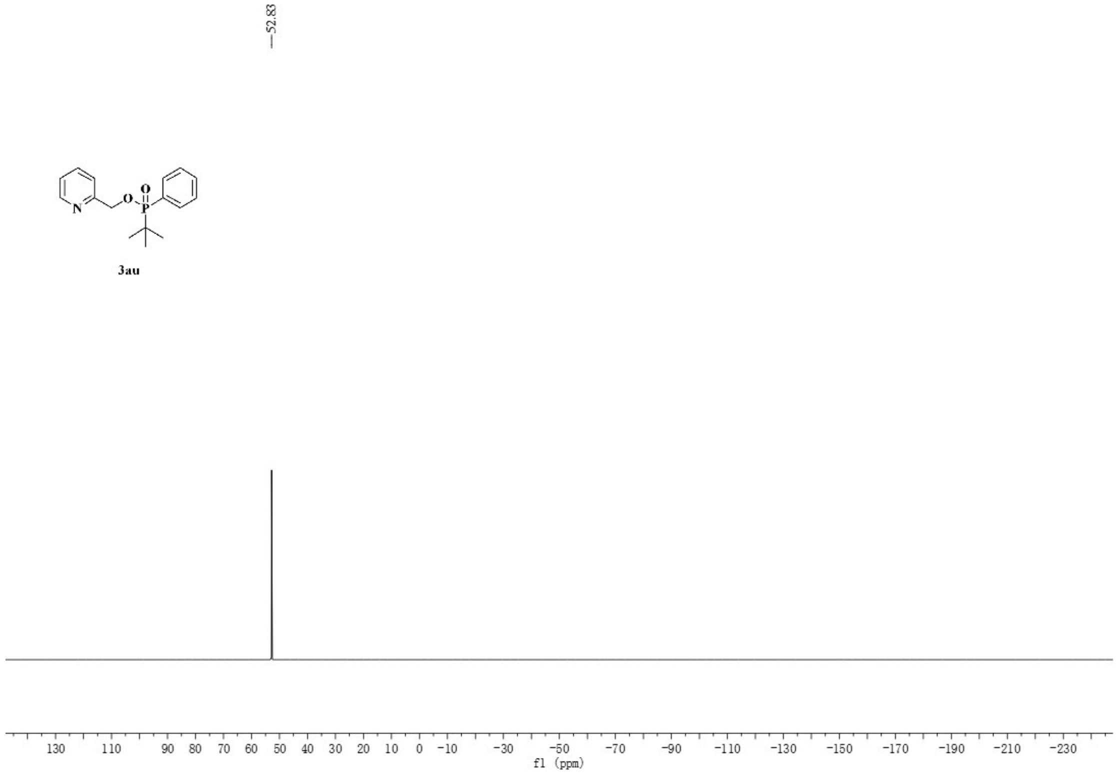

^{1}H NMR of **3ba** (400 MHz, $CDCl_3$)

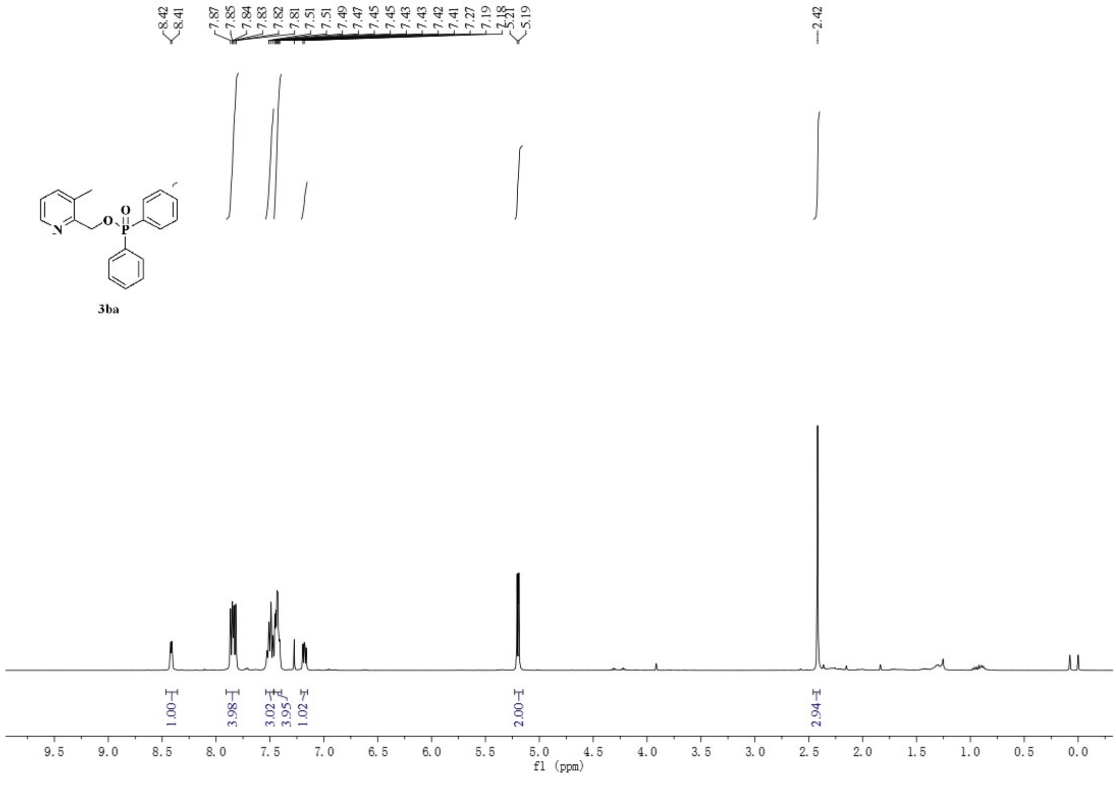

^{13}C NMR of **3ba** (101 MHz, $CDCl_3$)

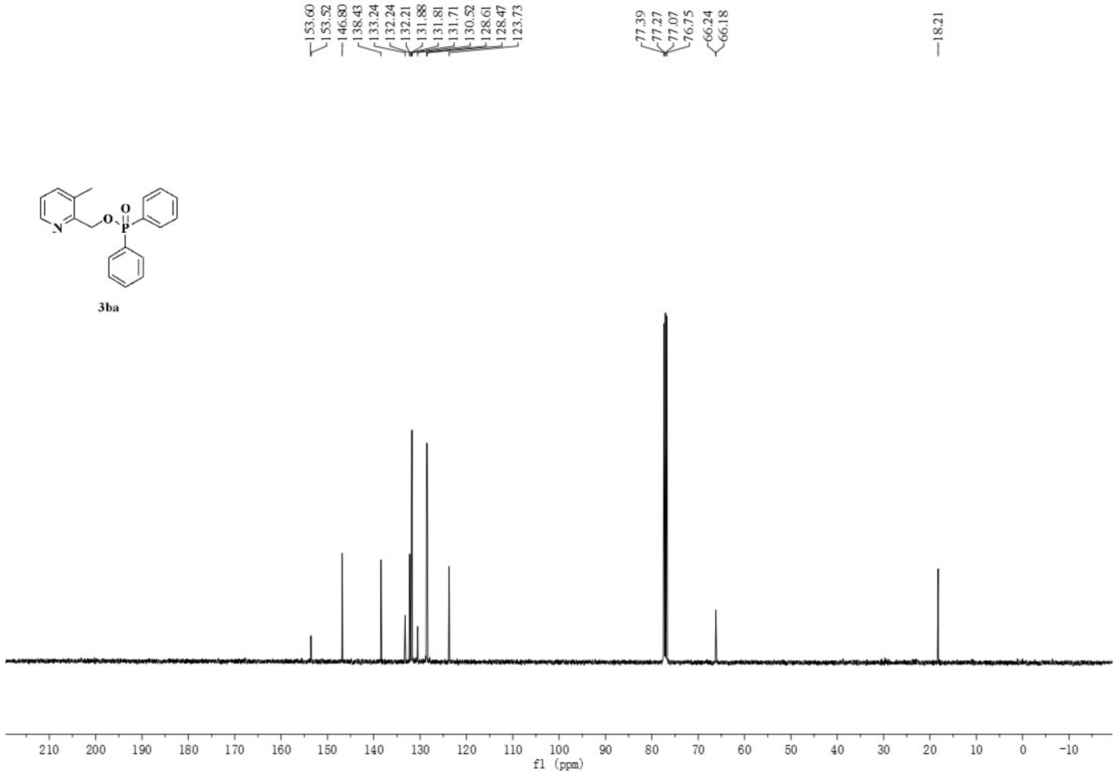

^{31}P NMR of **3ba** (121 MHz, $CDCl_3$)

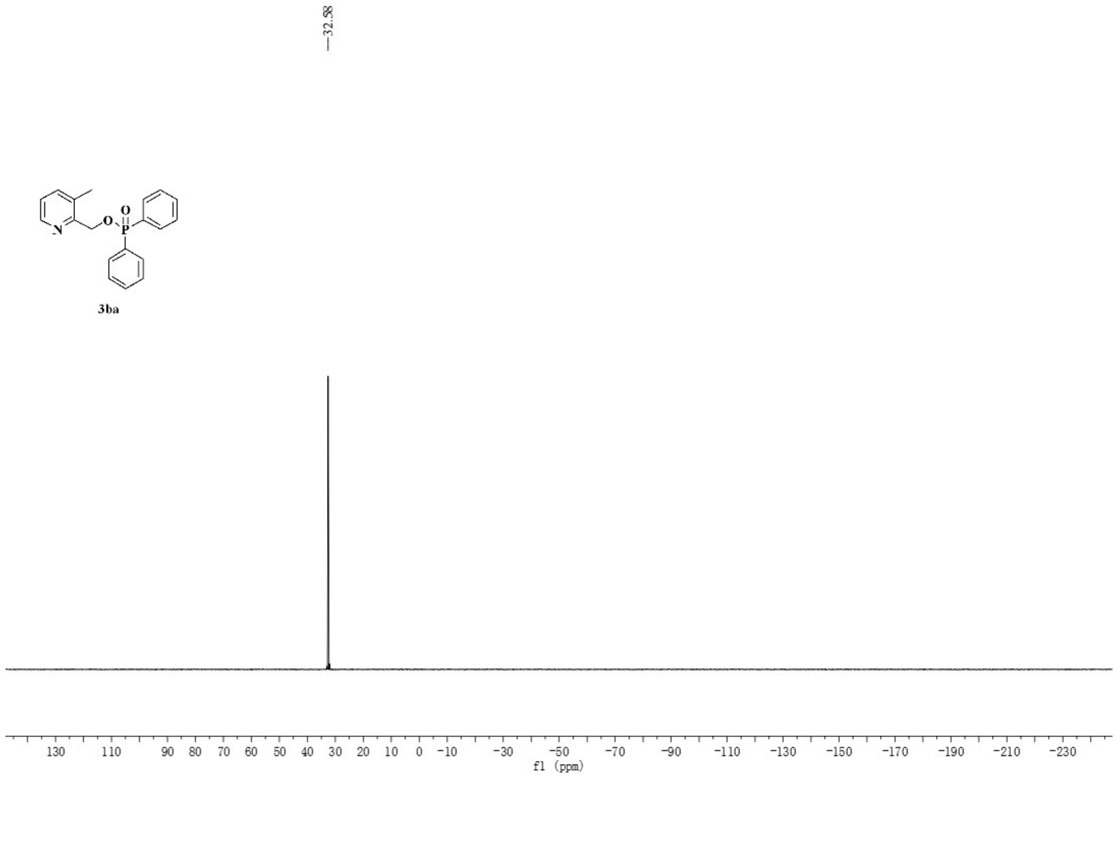

^{1}H NMR of **3bb** (400 MHz, $CDCl_3$)

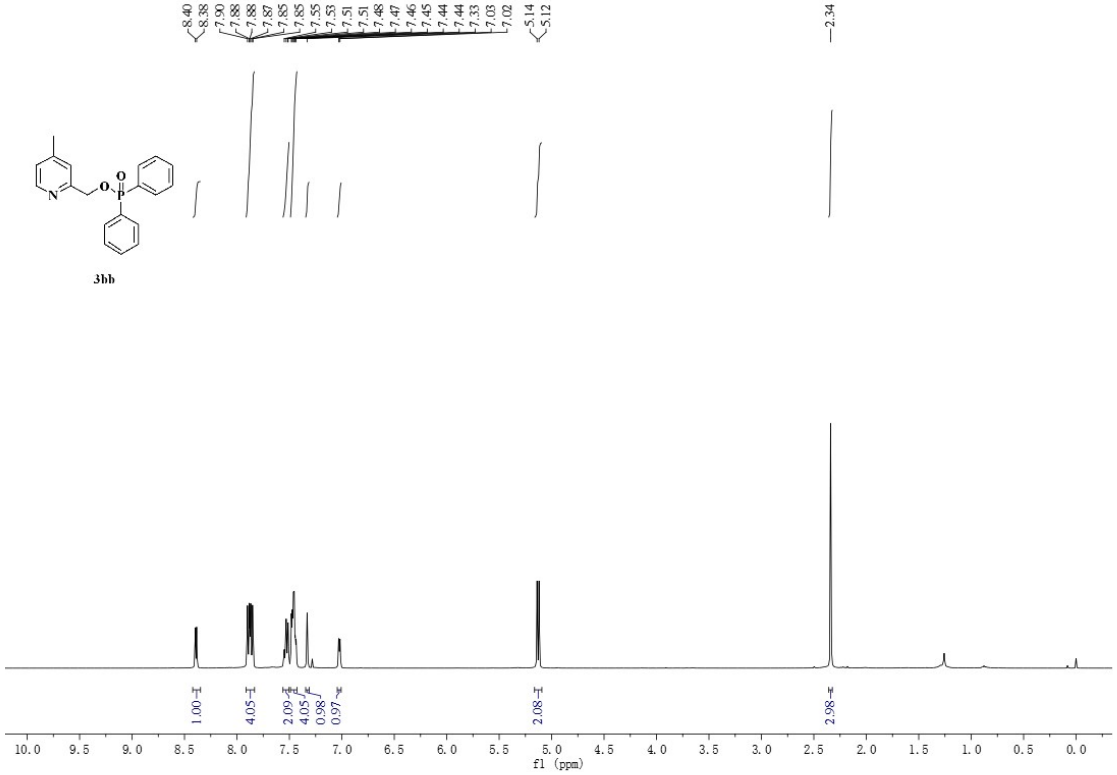

^{13}C NMR of **3bb** (101 MHz, $CDCl_3$)

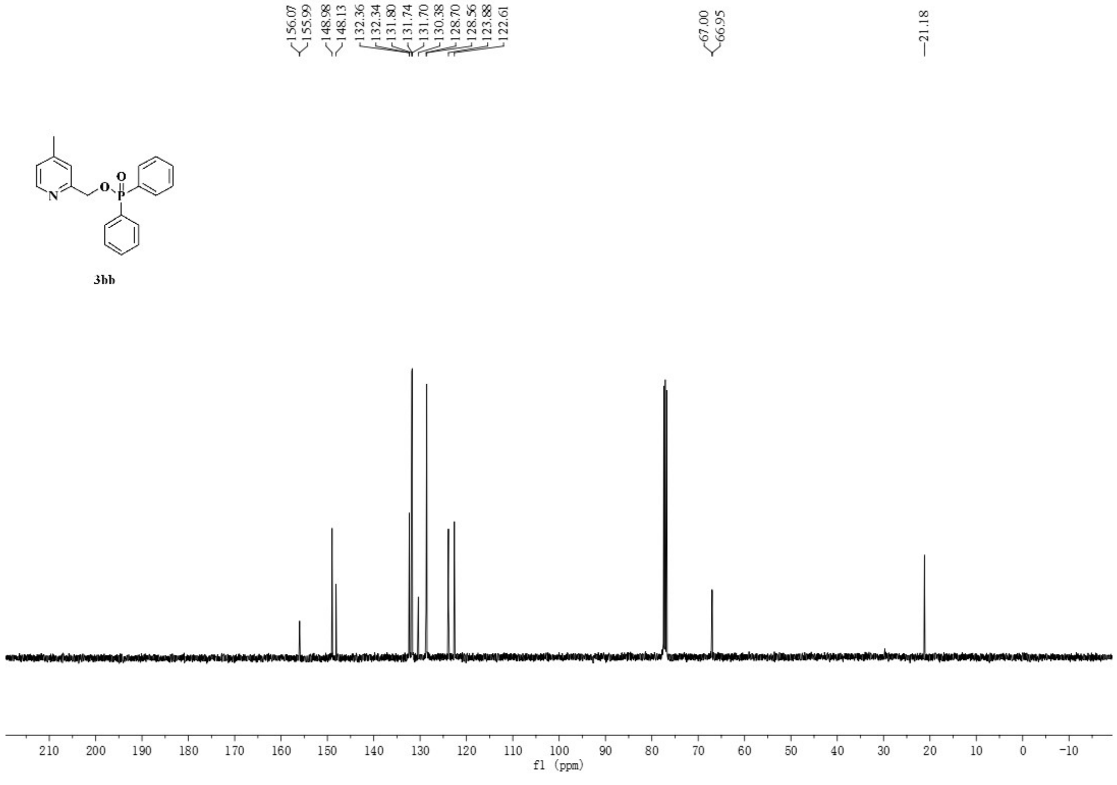

^{31}P NMR of **3bb** (121 MHz, $CDCl_3$)

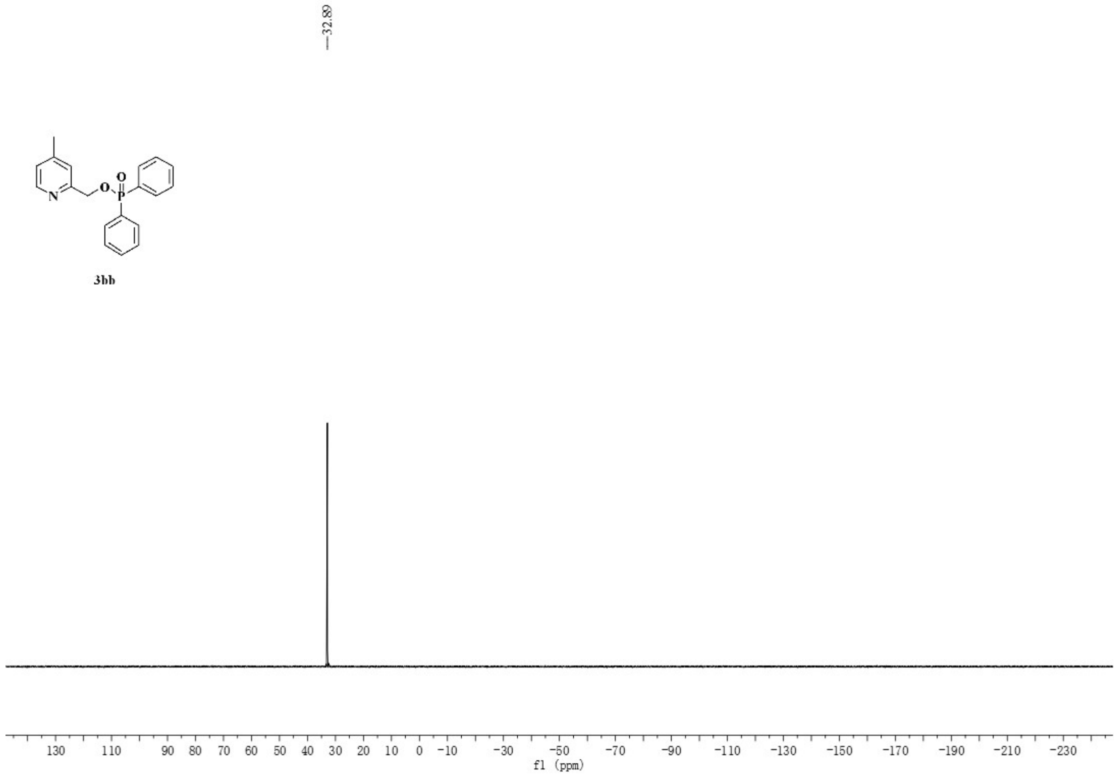

^{1}H NMR of **3bc** (400 MHz, $CDCl_3$)

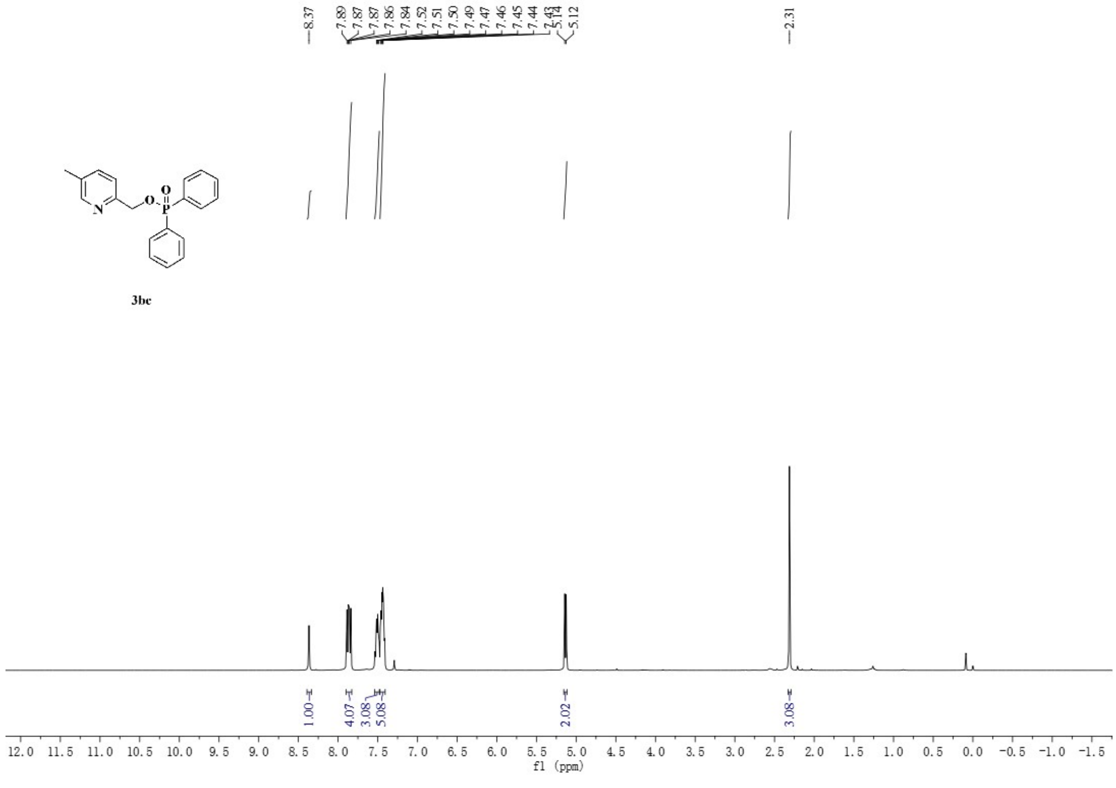

^{13}C NMR of **3bc** (101 MHz, $CDCl_3$)

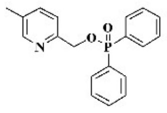

3bc

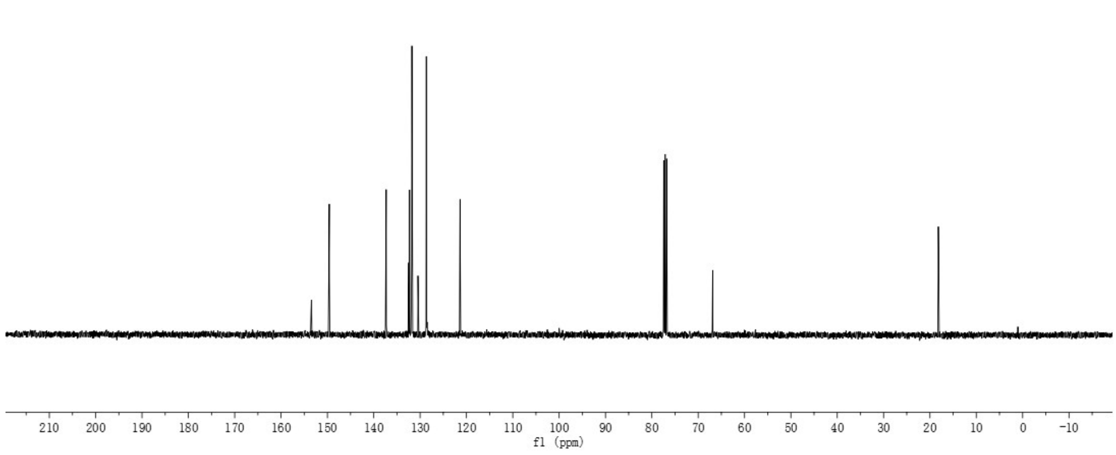

^{31}P NMR of **3bc** (121 MHz, $CDCl_3$)

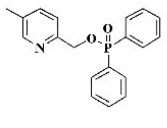

3bc

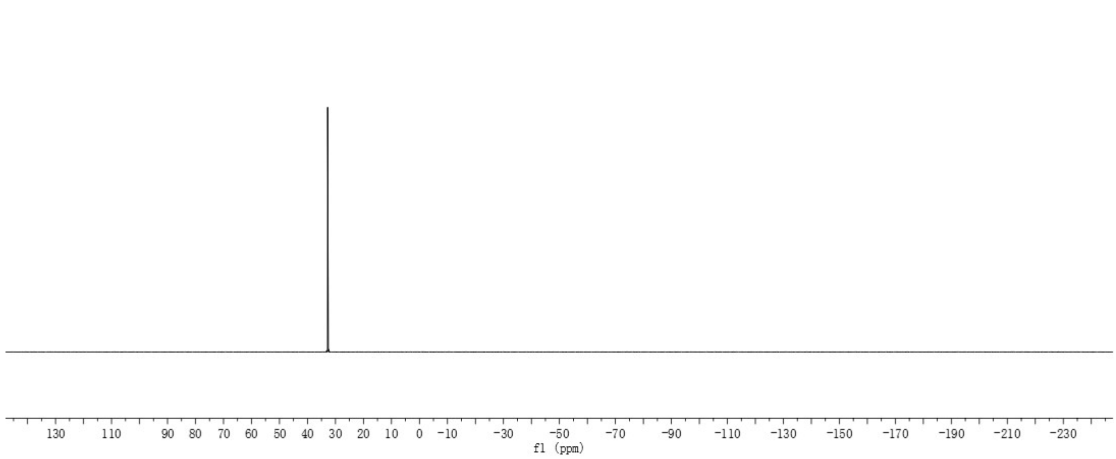

^{1}H NMR of **3bd** (400 MHz, $CDCl_3$)

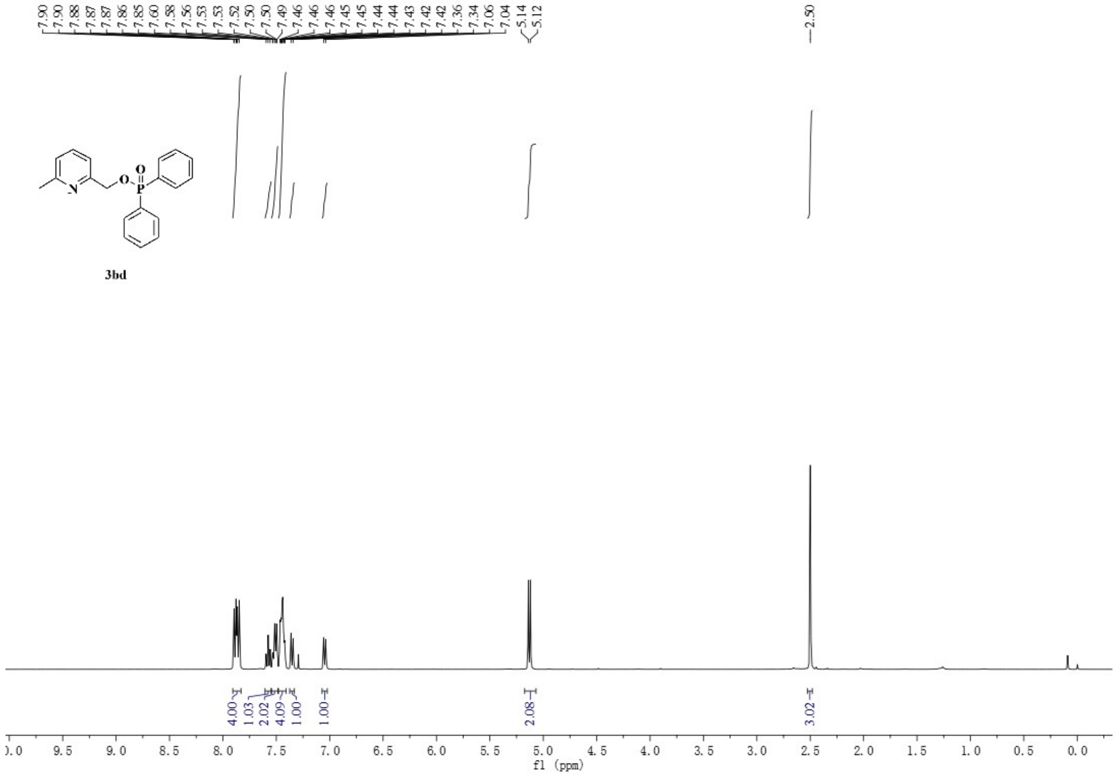

^{13}C NMR of **3bd** (101 MHz, $CDCl_3$)

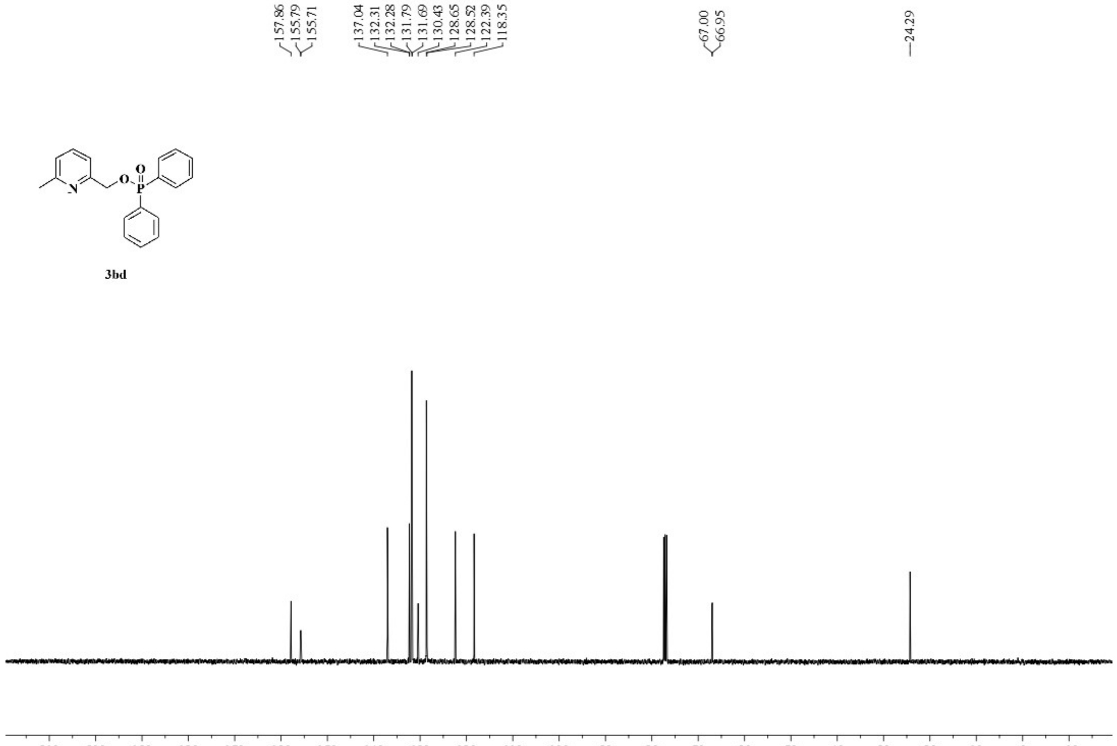

^{31}P NMR of **3bd** (121 MHz, $CDCl_3$)

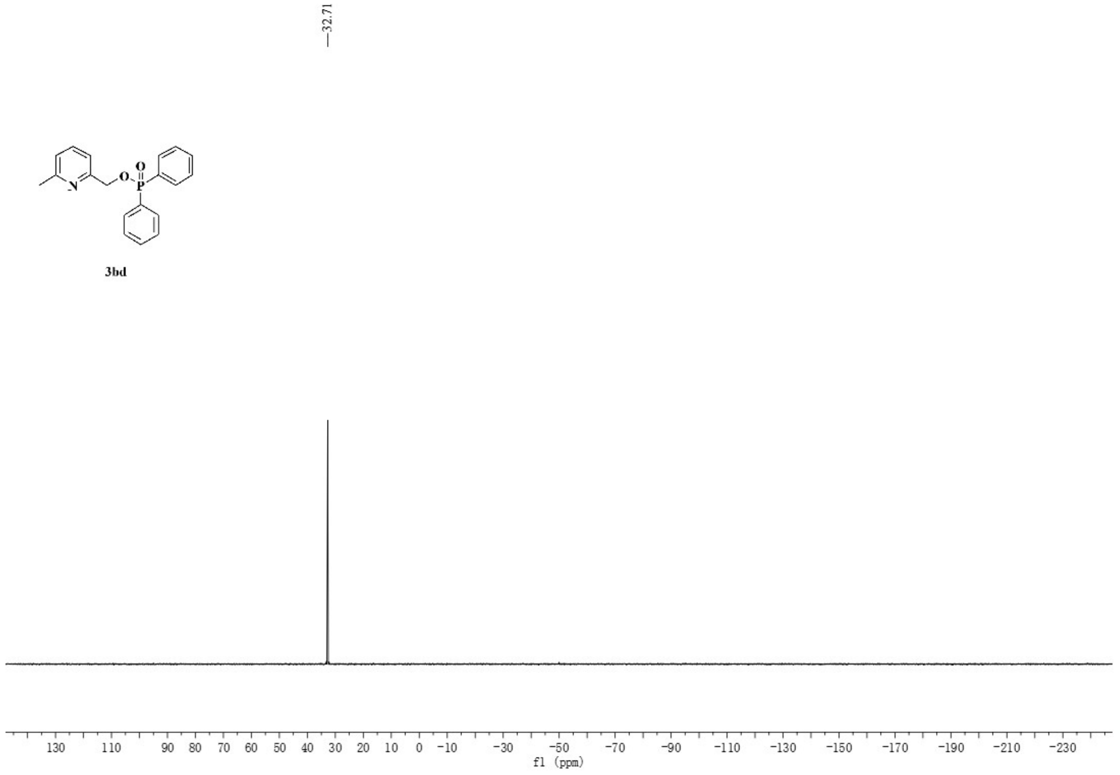

^{1}H NMR of **3be**(400 MHz, $CDCl_3$)

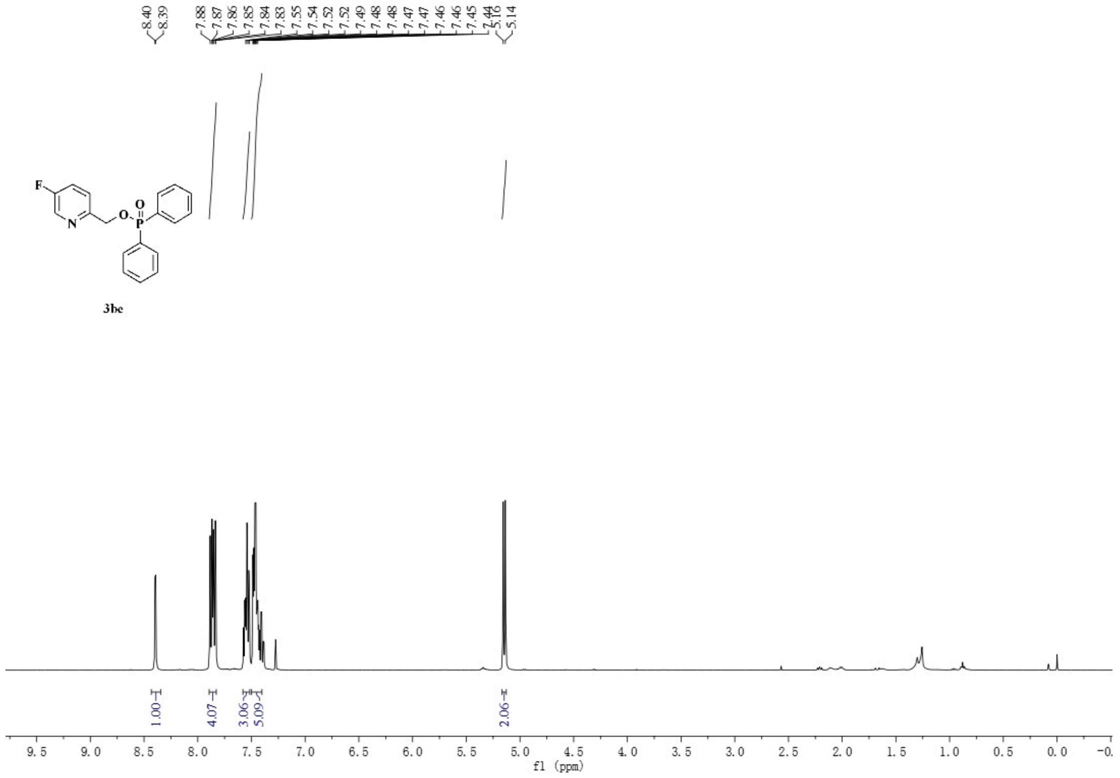

^{13}C NMR of **3be** (101 MHz, $CDCl_3$)

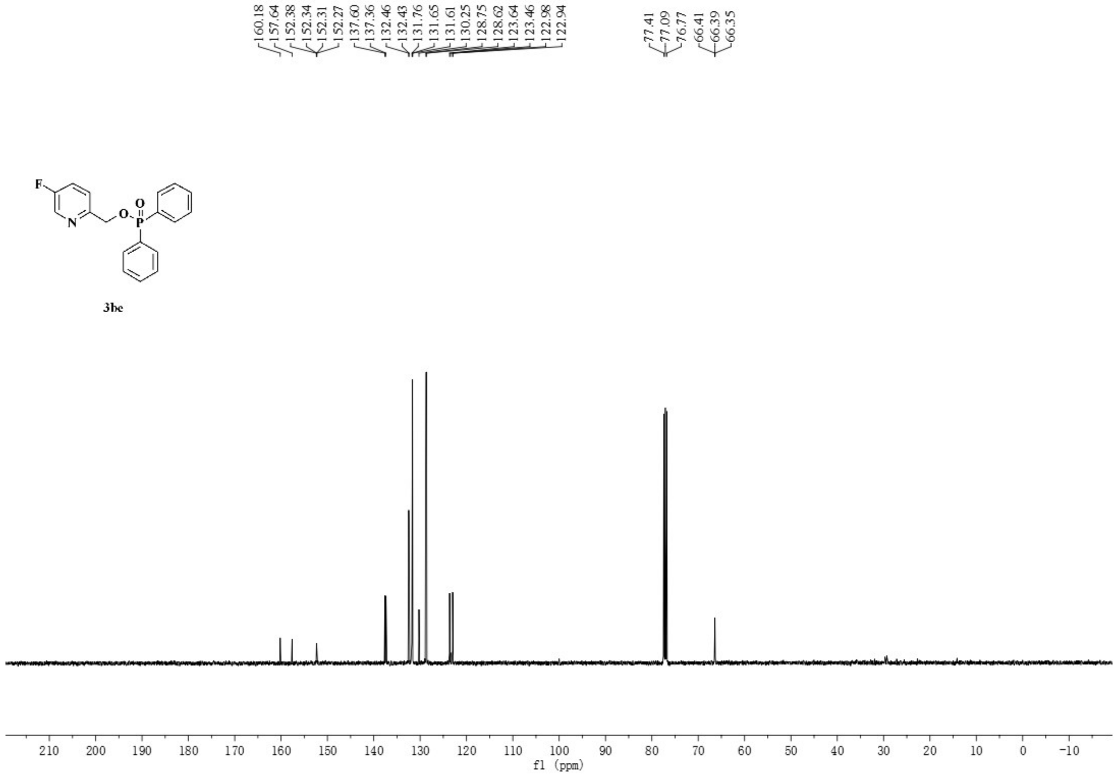

^{19}F NMR of **3be** (282 MHz, $CDCl_3$)

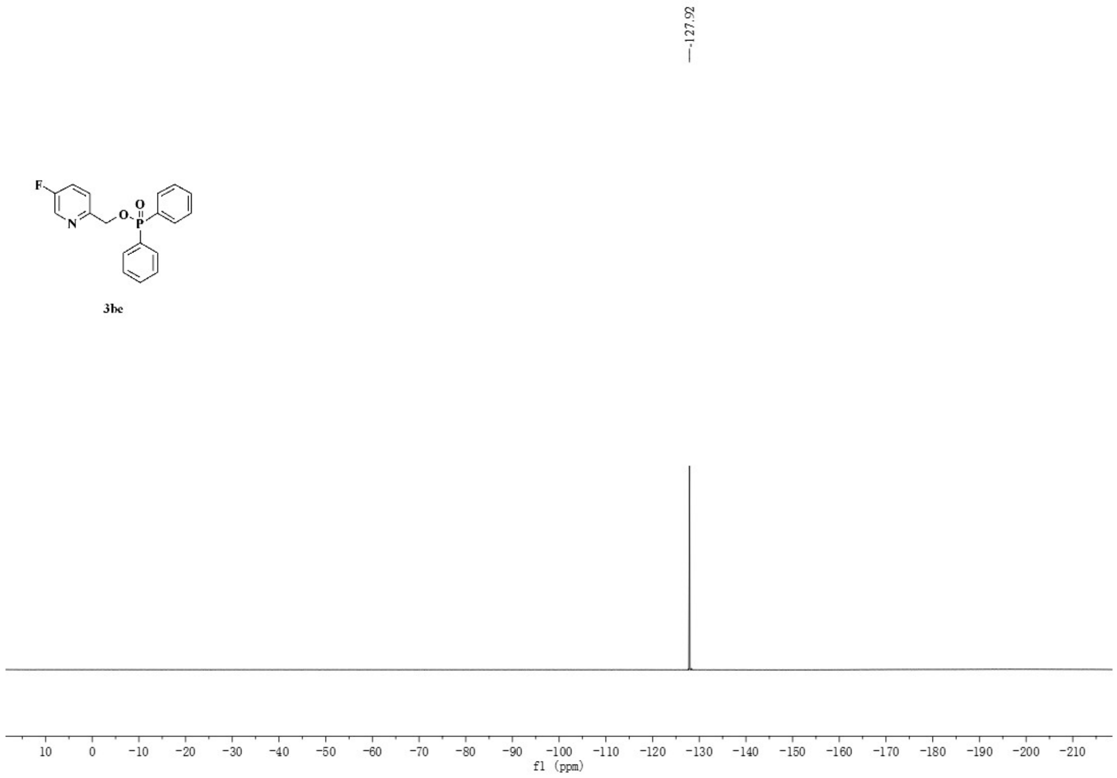

^{31}P NMR of **3be** (121 MHz, $CDCl_3$)

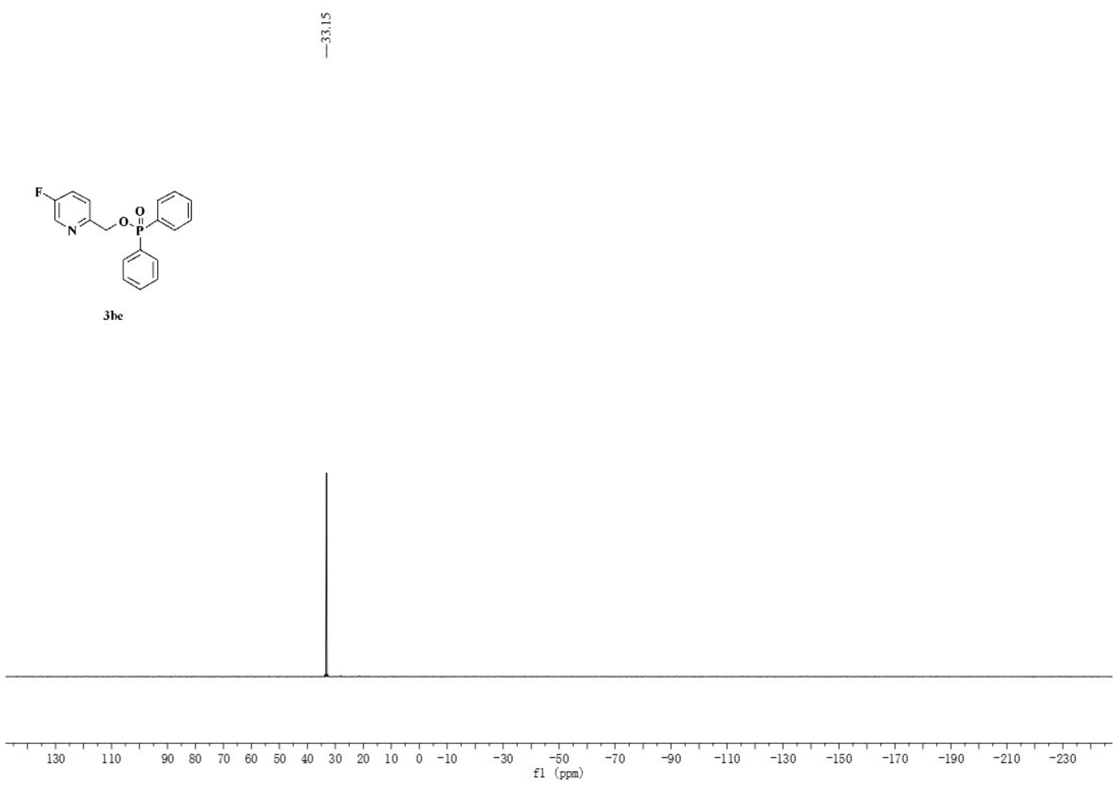

^{1}H NMR of **3bef**(400 MHz, $CDCl_3$)

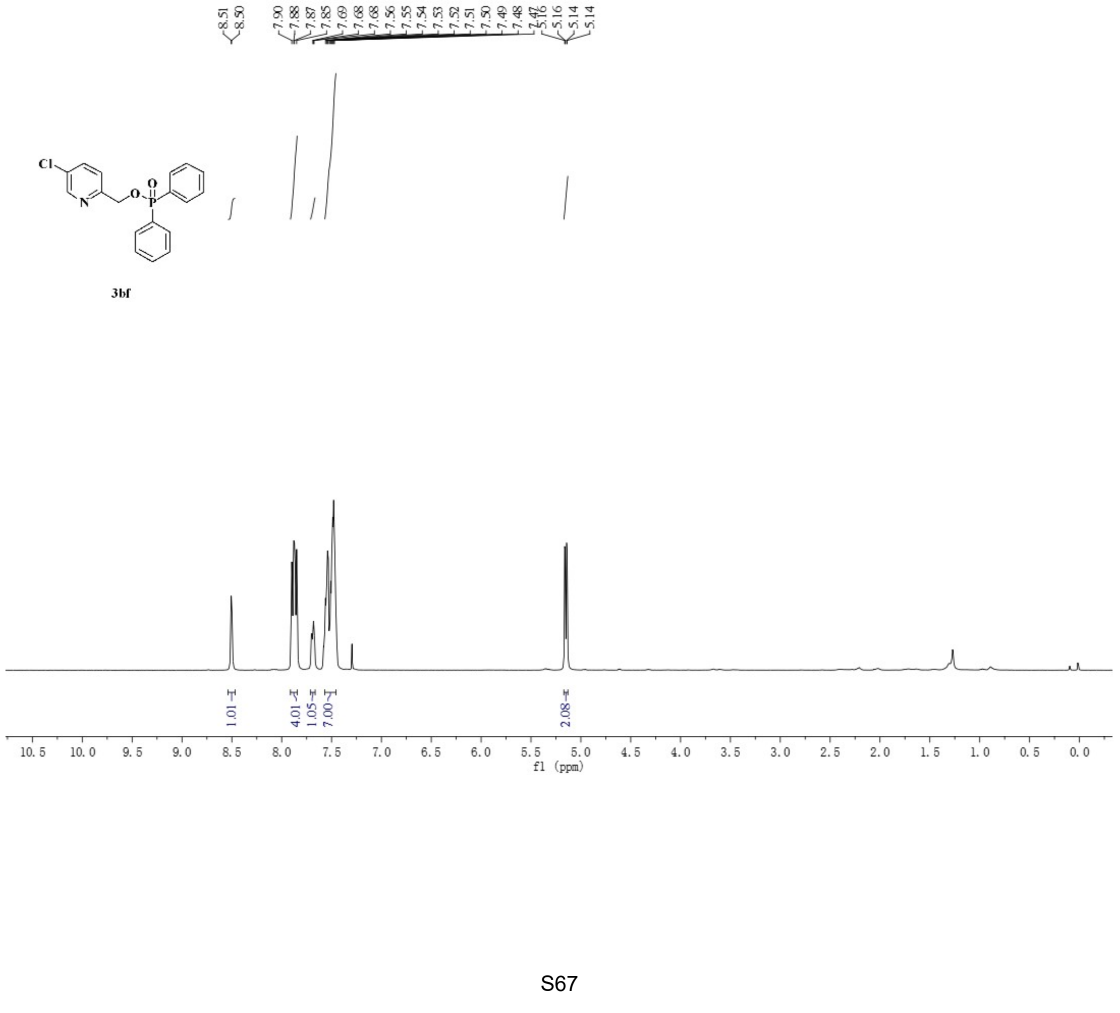

^{13}C NMR of **3bf** (101 MHz, $CDCl_3$)

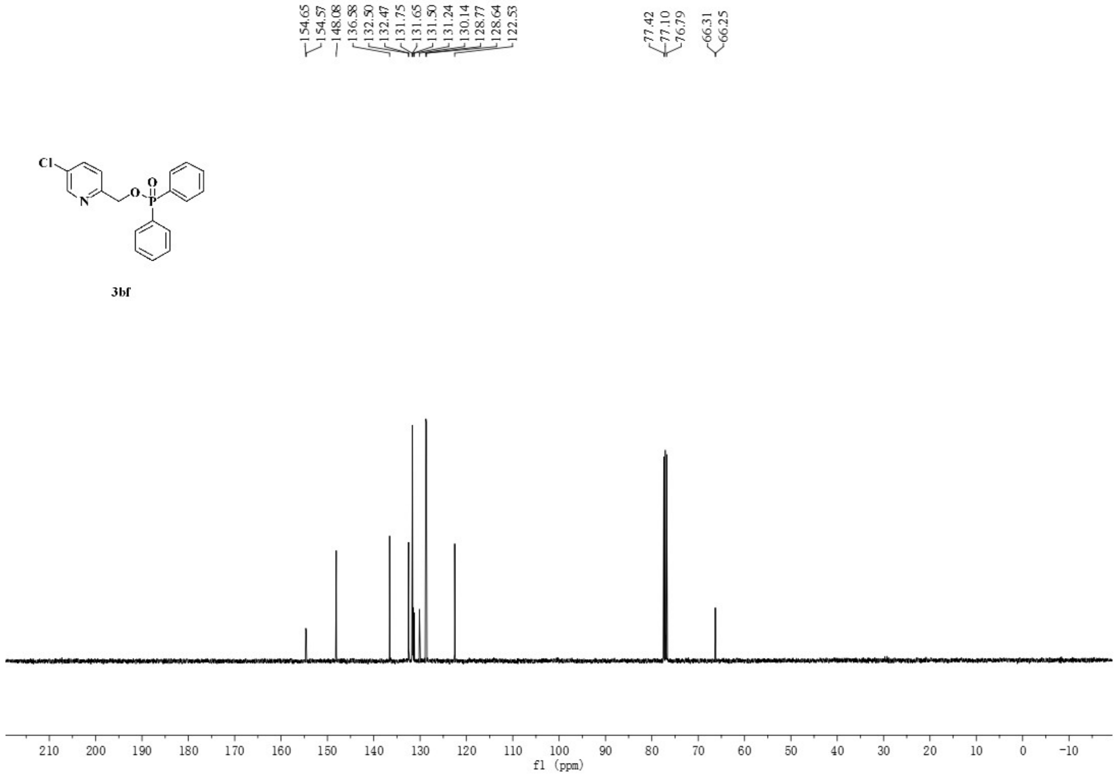

^{31}P NMR of **3bf** (121 MHz, $CDCl_3$)

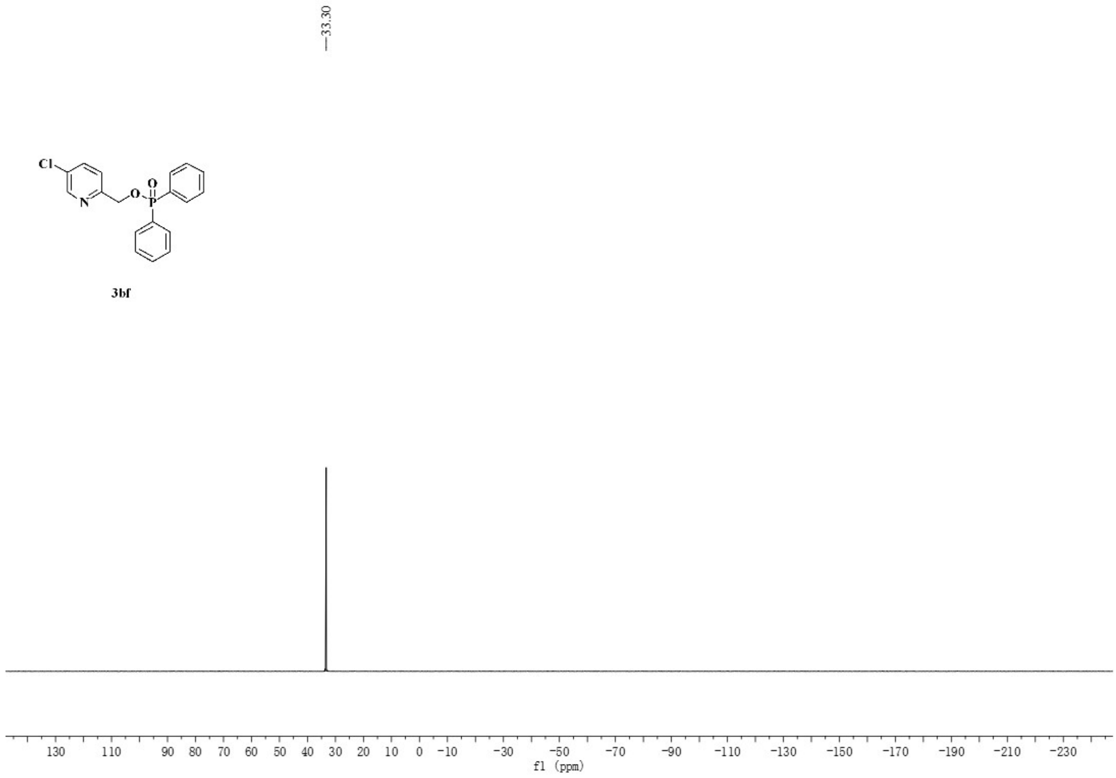

^{1}H NMR of **3bg** (400 MHz, $CDCl_3$)

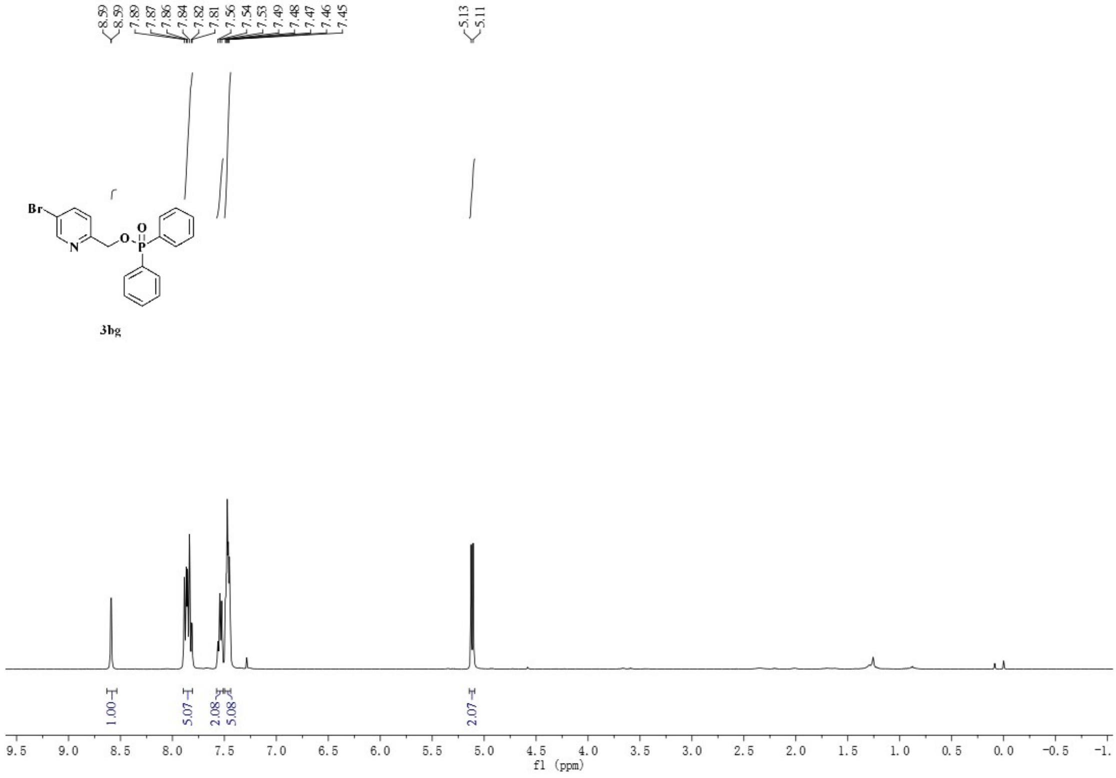

^{13}C NMR of **3bg** (101 MHz, $CDCl_3$)

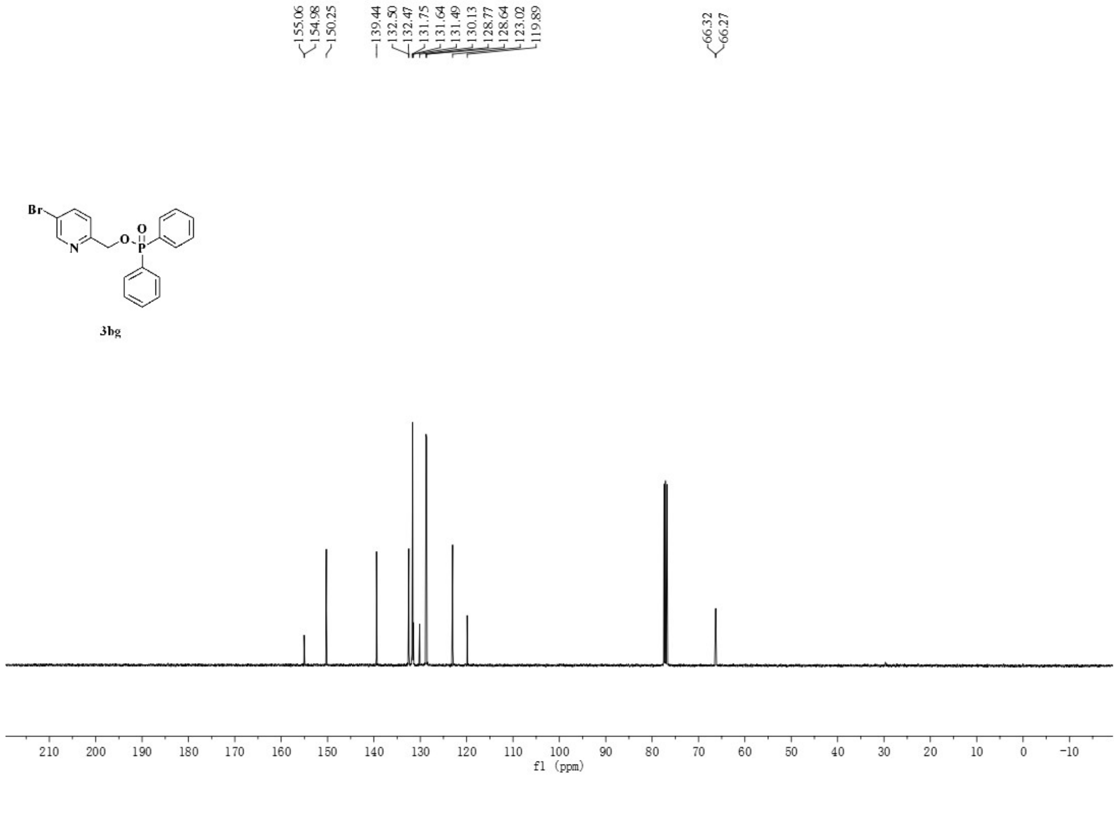

^{31}P NMR of **3bg** (121 MHz, $CDCl_3$)

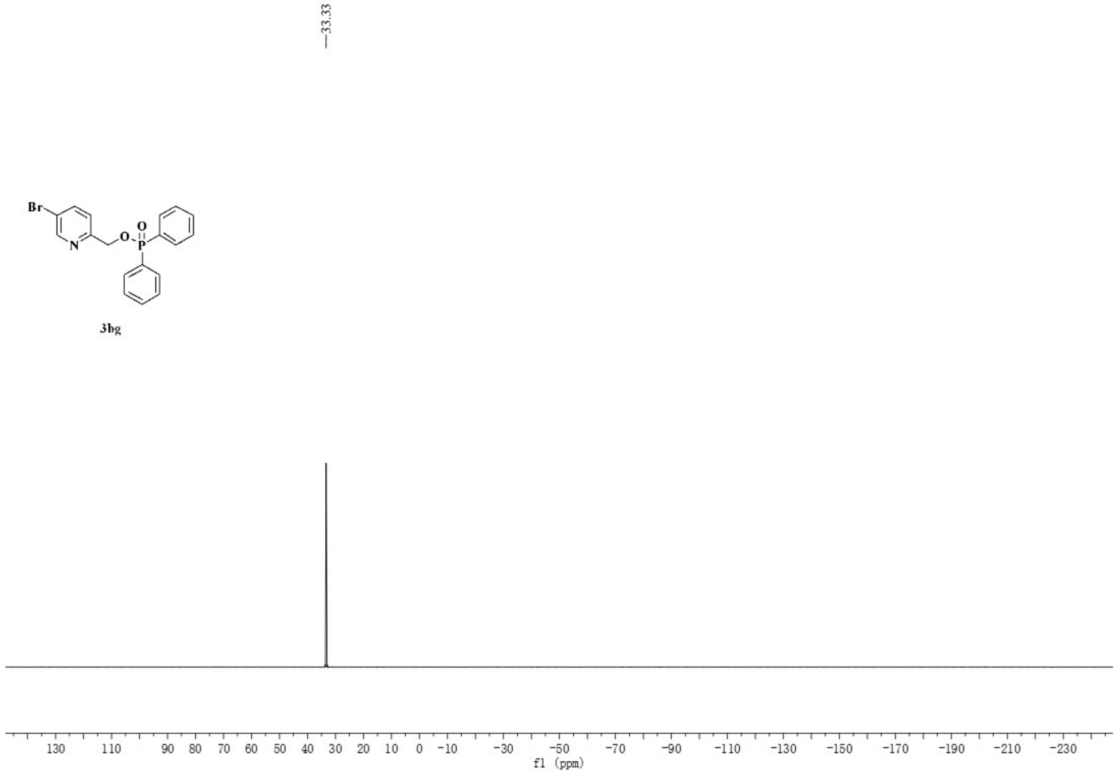

^{1}H NMR of **3bh** (400 MHz, $CDCl_3$)

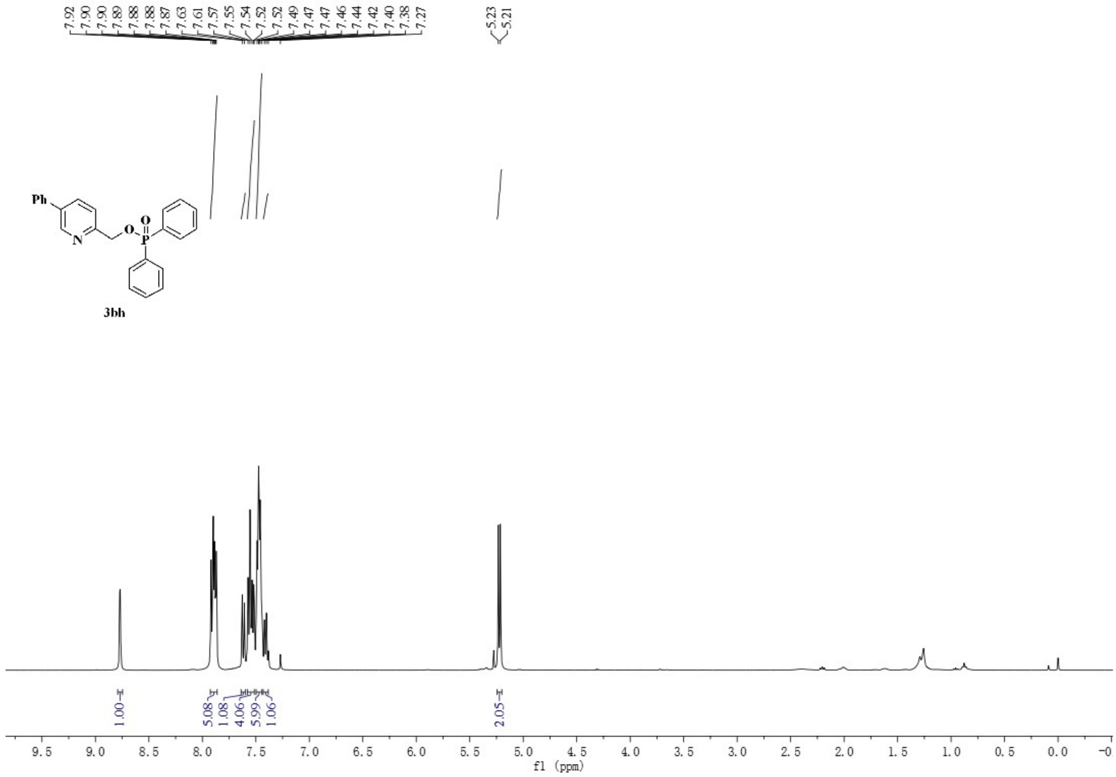

^{13}C NMR of **3bh** (101 MHz, $CDCl_3$)

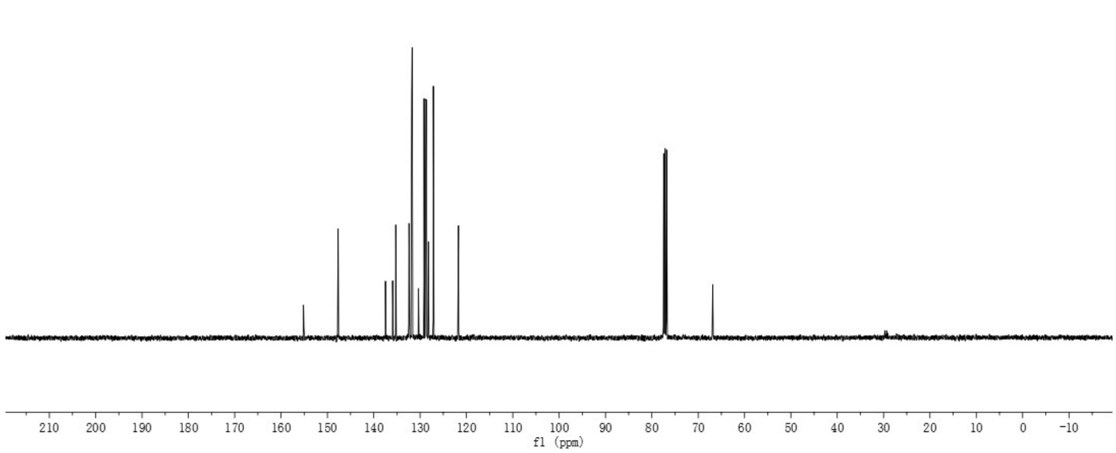

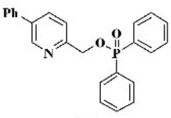

3bh

^{31}P NMR of **3bh** (121 MHz, $CDCl_3$)

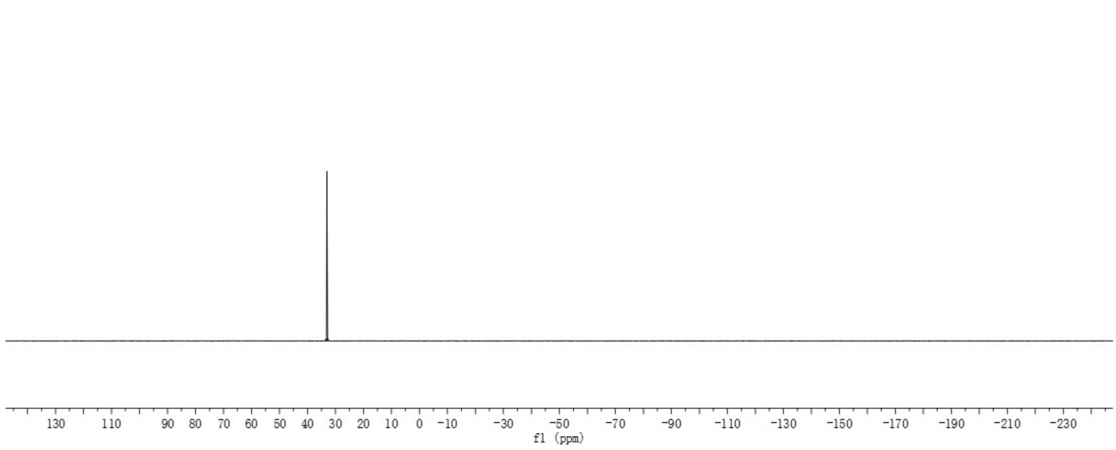

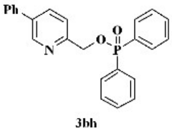

3bh

^{1}H NMR of **3bi** (400 MHz, $CDCl_3$)

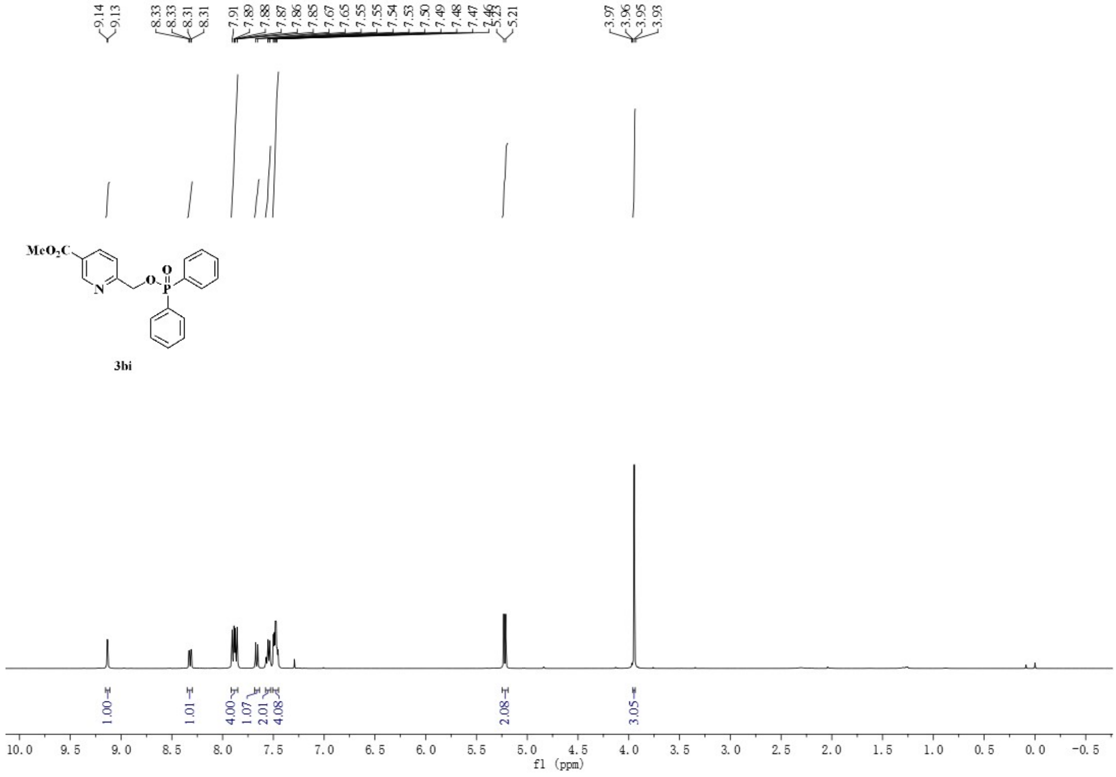

^{13}C NMR of **3bi** (101 MHz, $CDCl_3$)

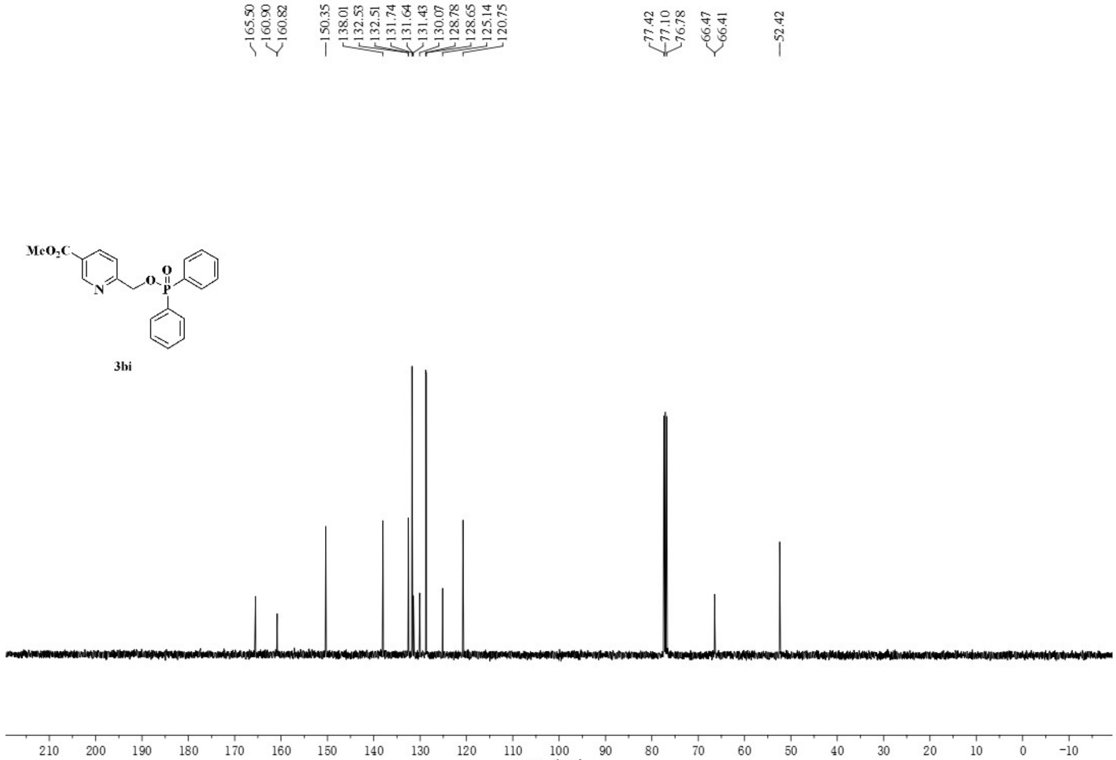

^{31}P NMR of **3bi** (121 MHz, $CDCl_3$)

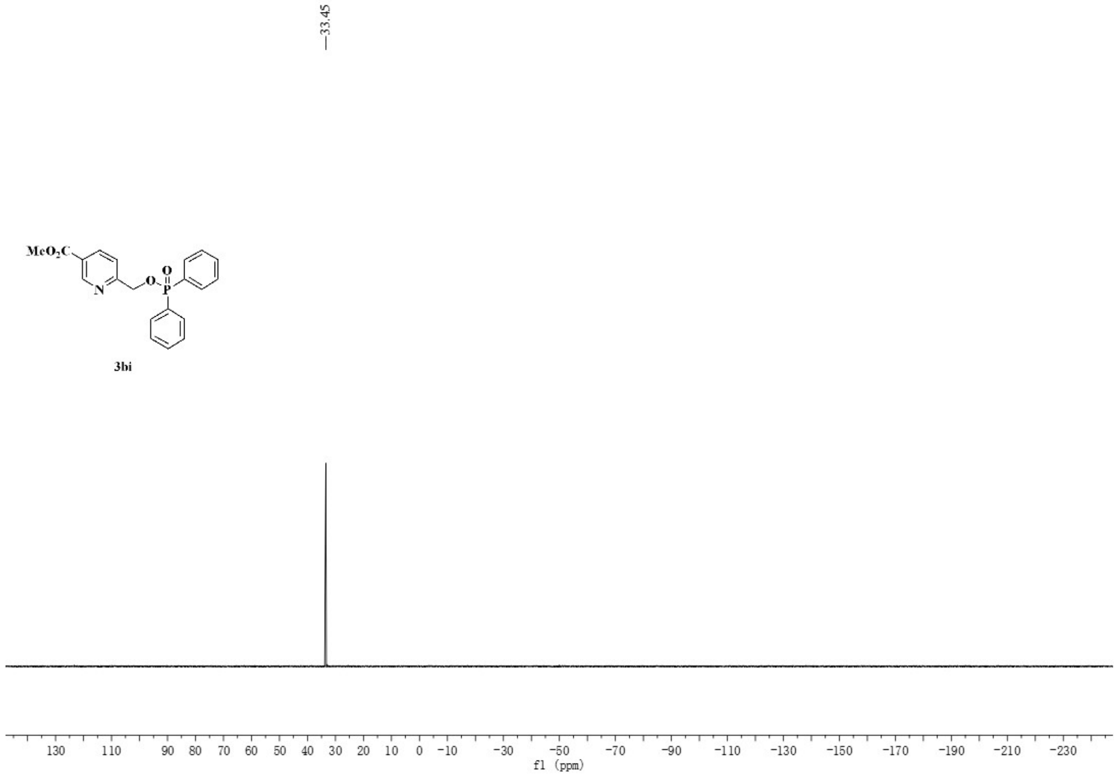

^{1}H NMR of **3bj** (400 MHz, $CDCl_3$)

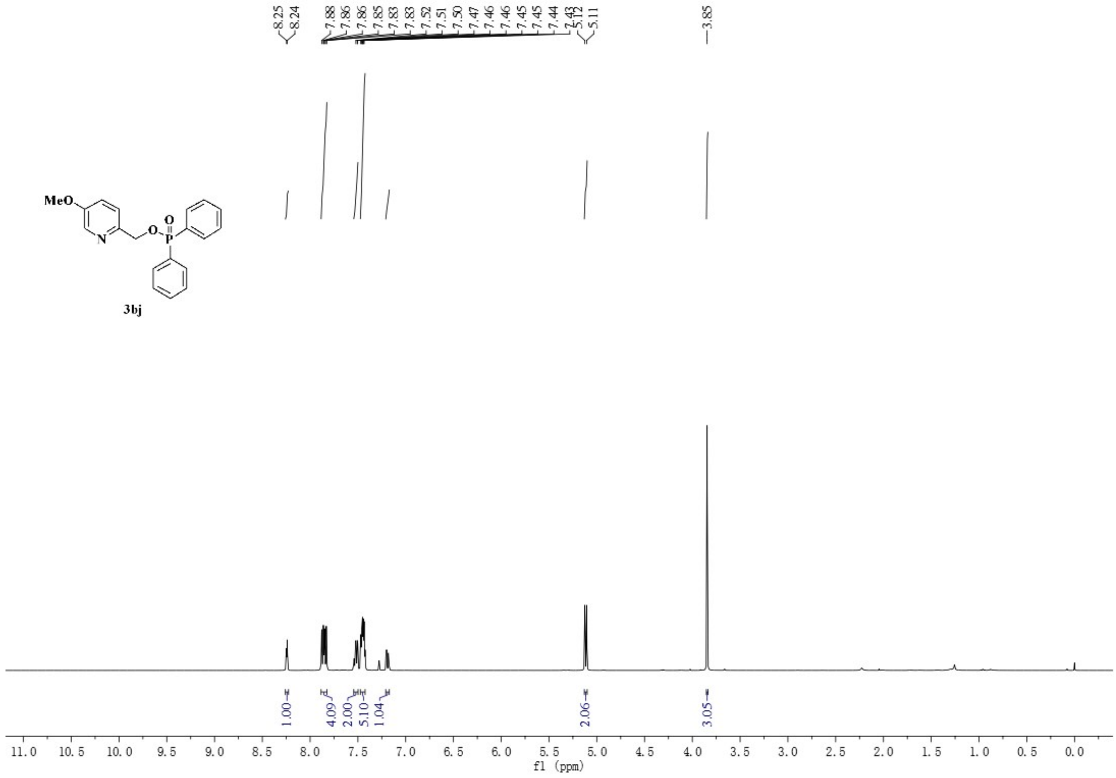

^{13}C NMR of **3bj** (101 MHz, $CDCl_3$)

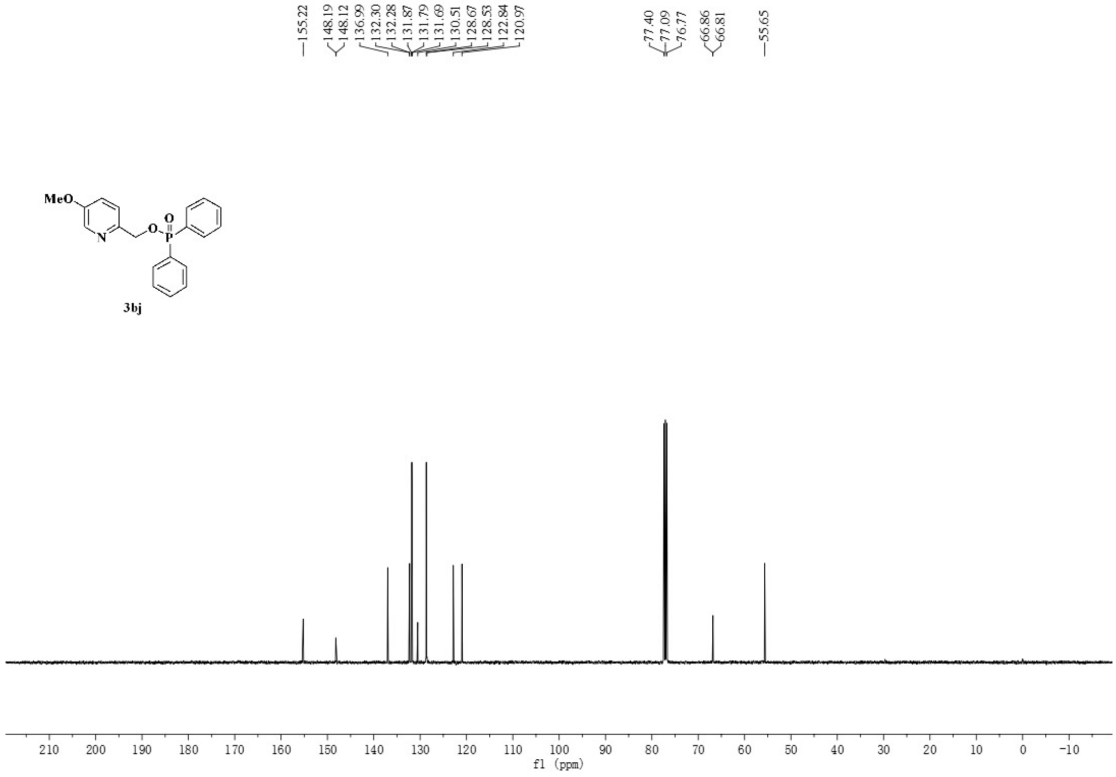

^{31}P NMR of **3bj** (121 MHz, $CDCl_3$)

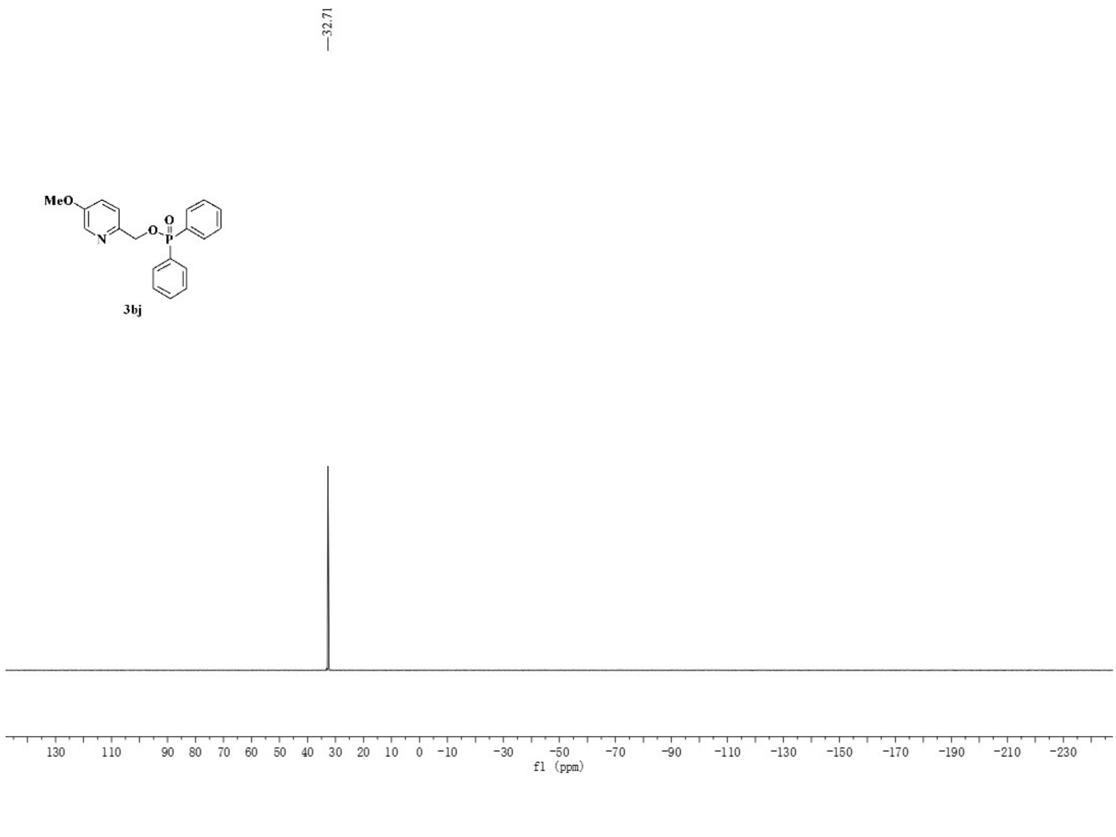

^{1}H NMR of **3bk** (400 MHz, $CDCl_3$)

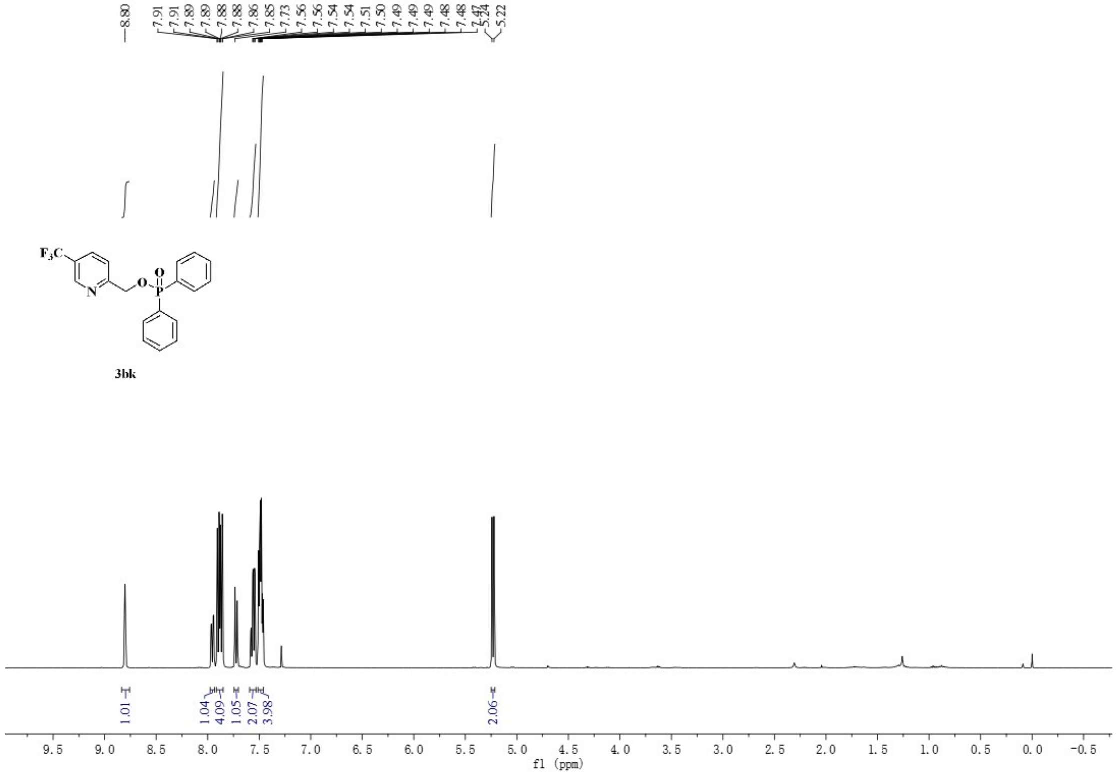

^{13}C NMR of **3bk** (101 MHz, $CDCl_3$)

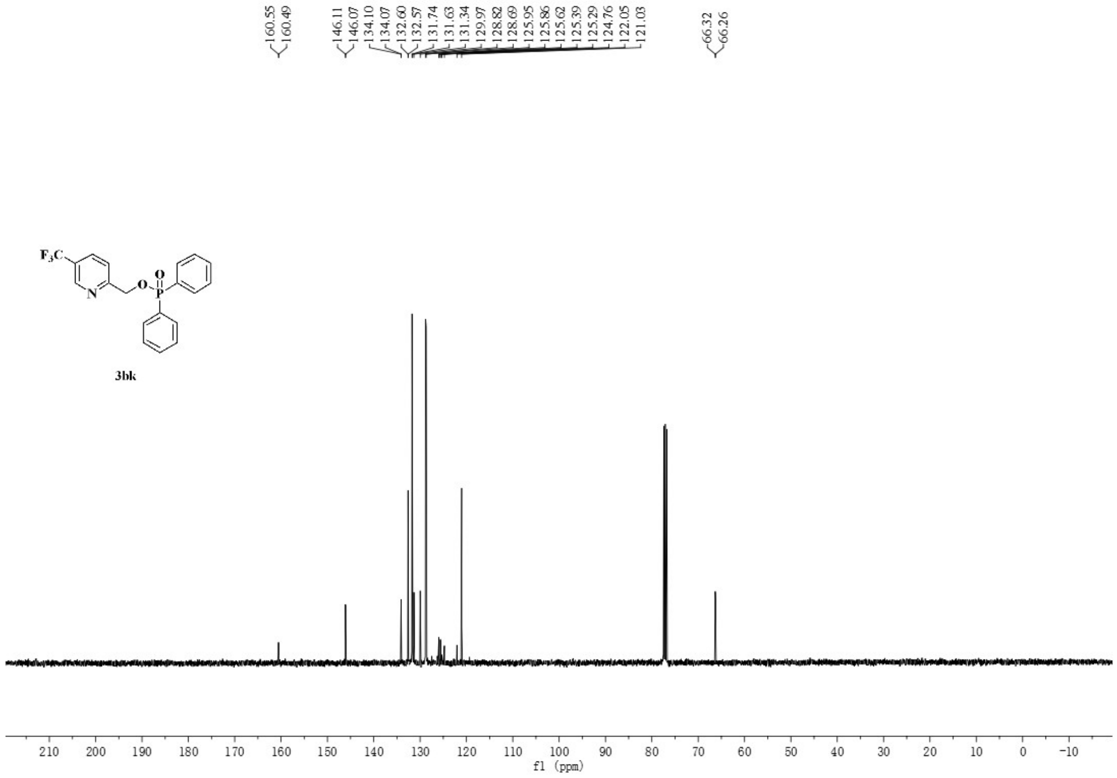

^{19}F NMR of **3bk** (282 MHz, $CDCl_3$)

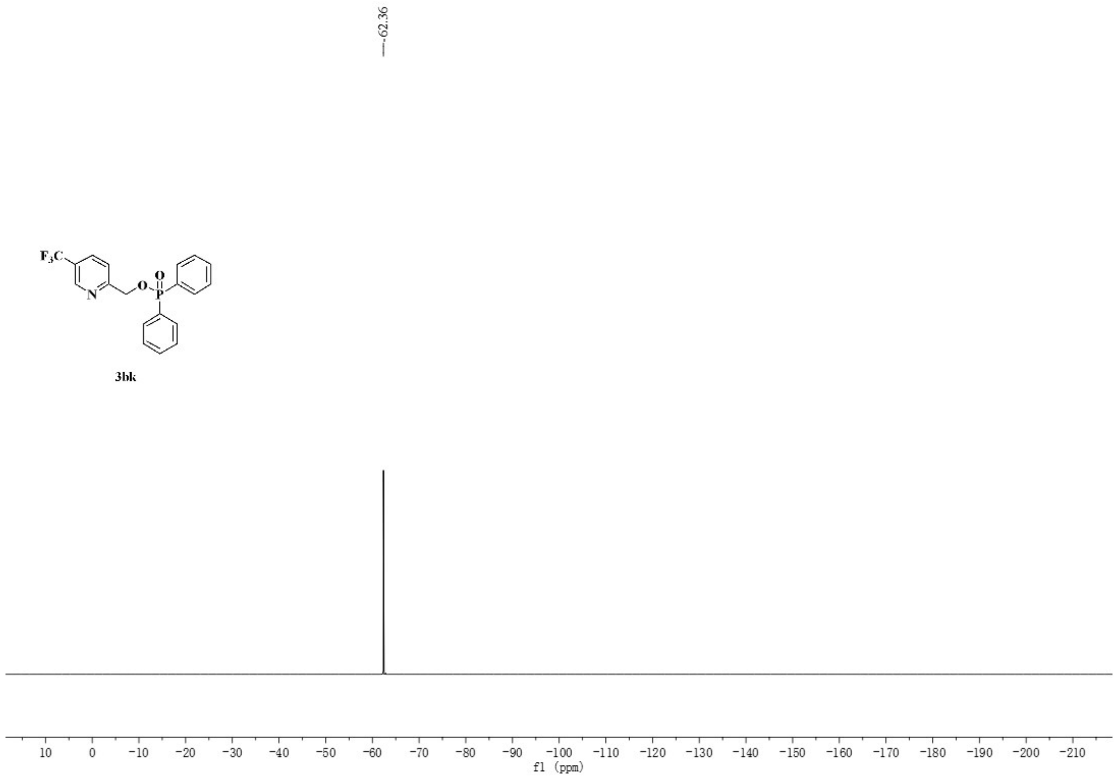

^{31}P NMR of **3bk** (121 MHz, $CDCl_3$)

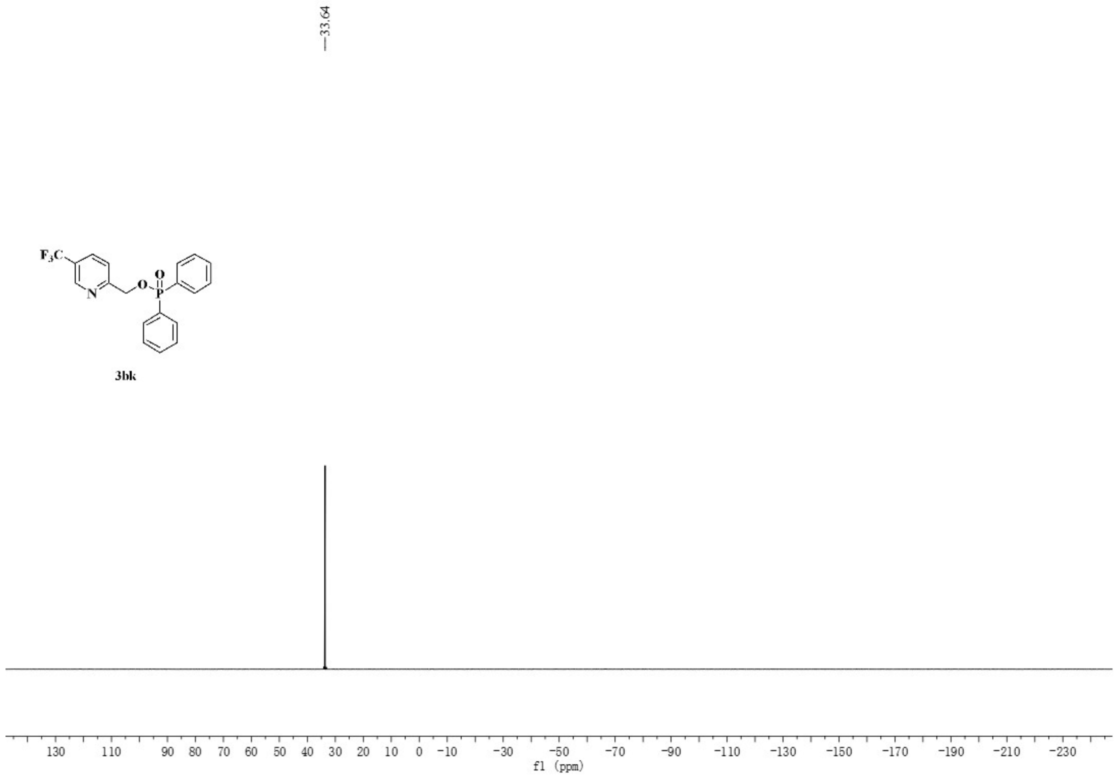

^{1}H NMR of **3bl** (400 MHz, $CDCl_3$)

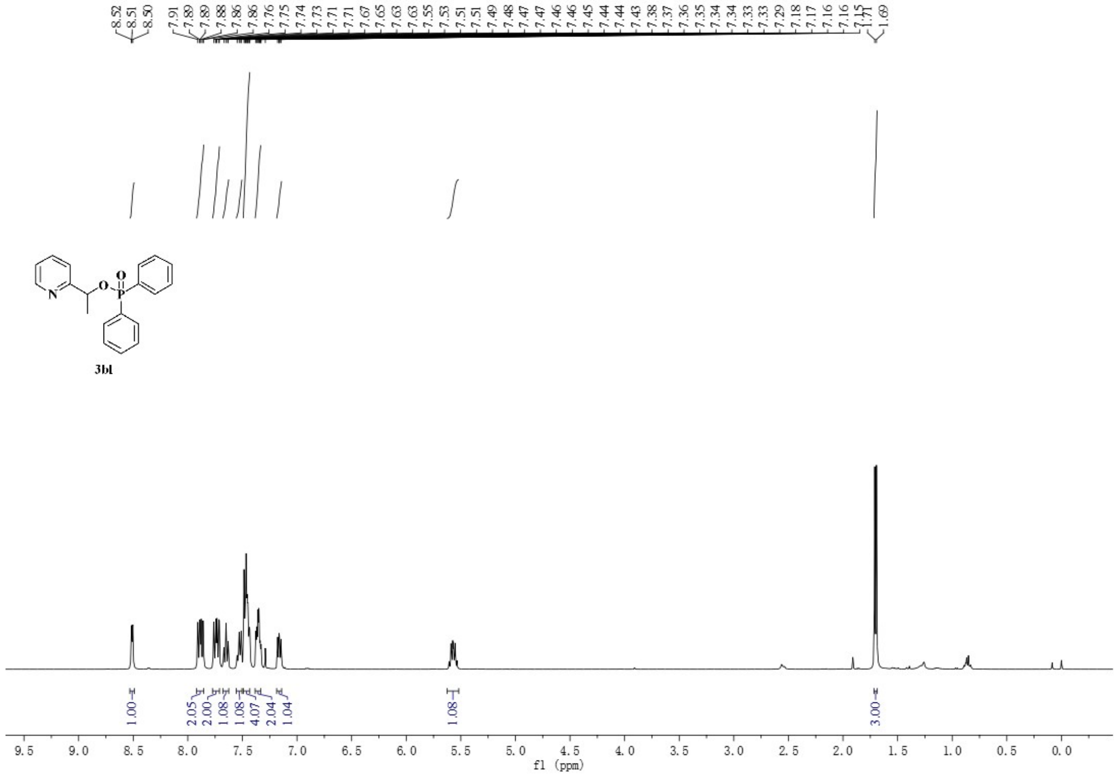

^{13}C NMR of **3bl** (101 MHz, $CDCl_3$)

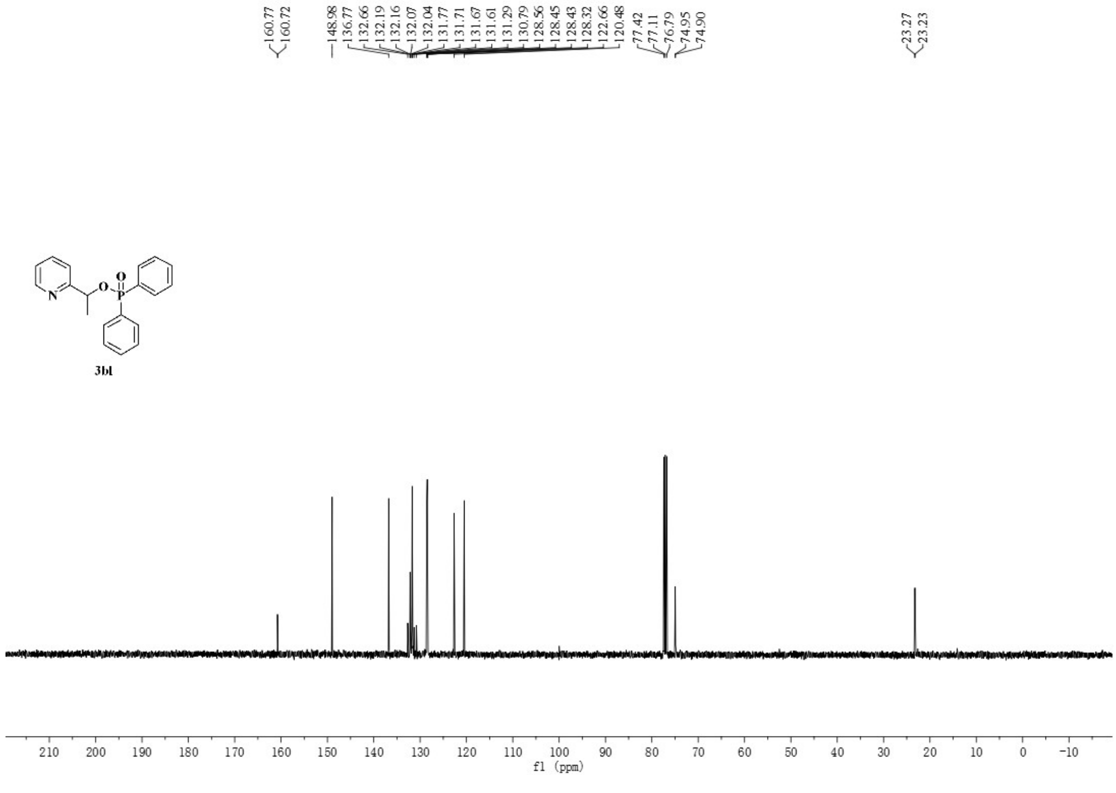

^{31}P NMR of **3bl** (121 MHz, $CDCl_3$)

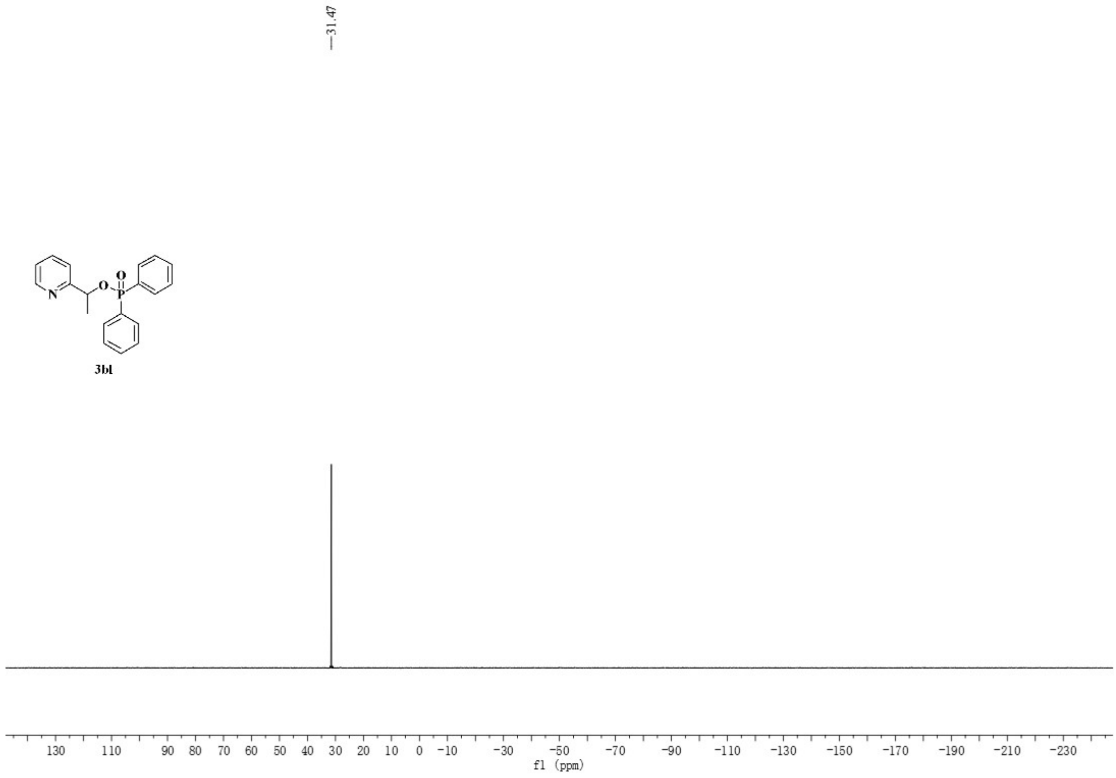

^{1}H NMR of **3bm** (400 MHz, $CDCl_3$)

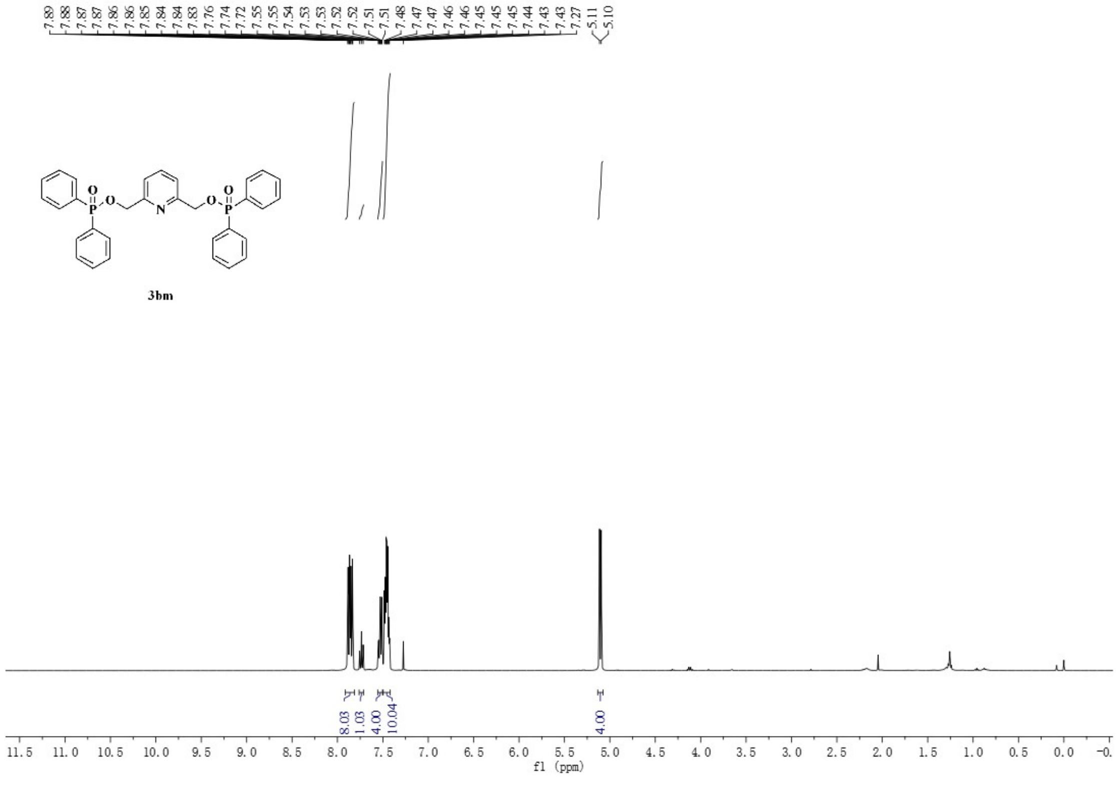

^{13}C NMR of **3bm** (101 MHz, $CDCl_3$)

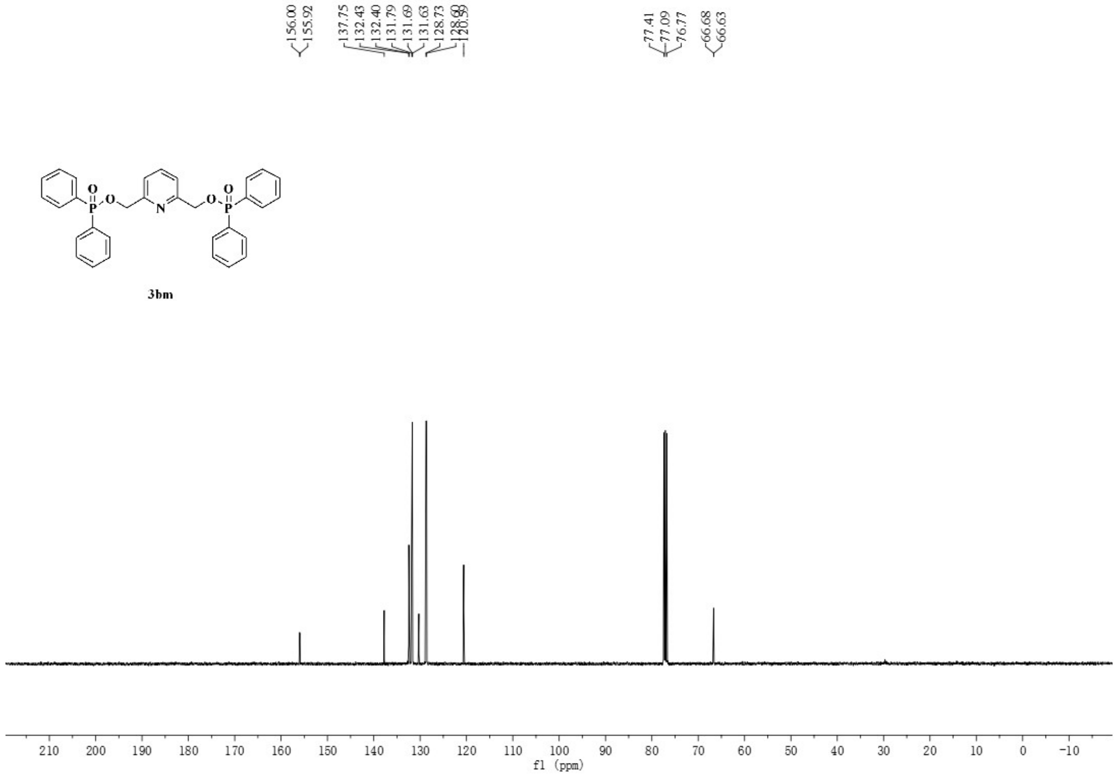

^{31}P NMR of **3bm** (121 MHz, $CDCl_3$)

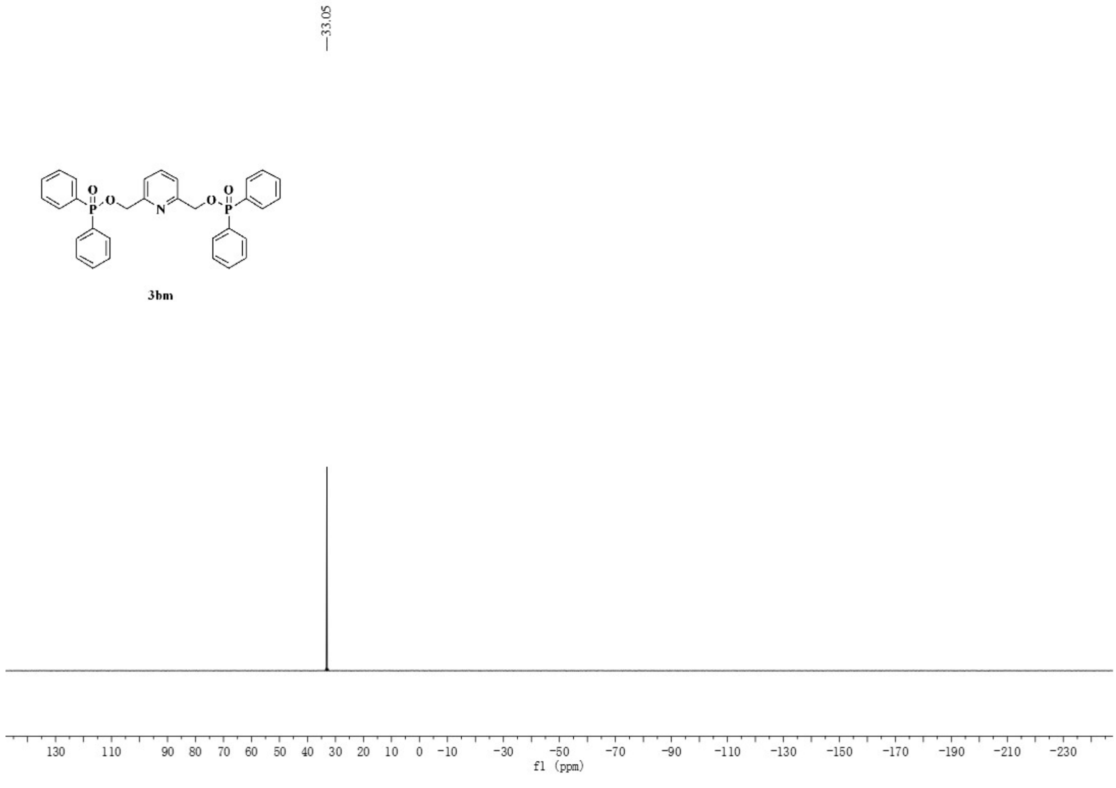

^{1}H NMR of **3bn** (400 MHz, $CDCl_3$)

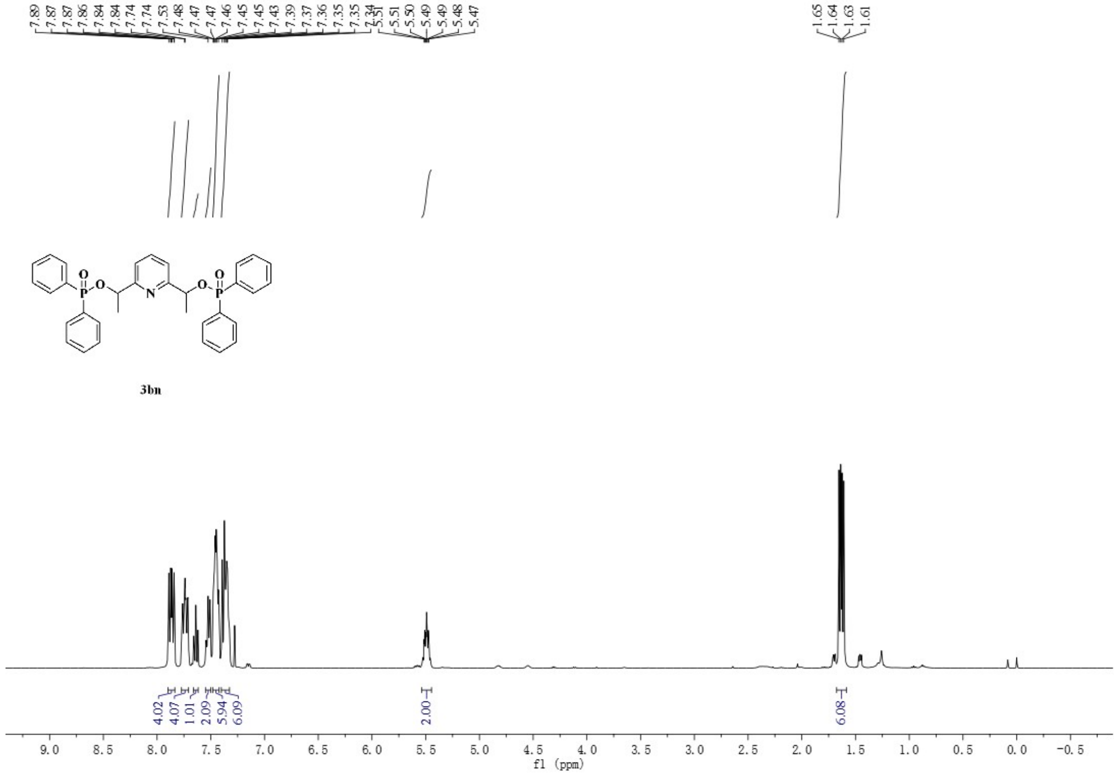

^{13}C NMR of **3bn** (101 MHz, $CDCl_3$)

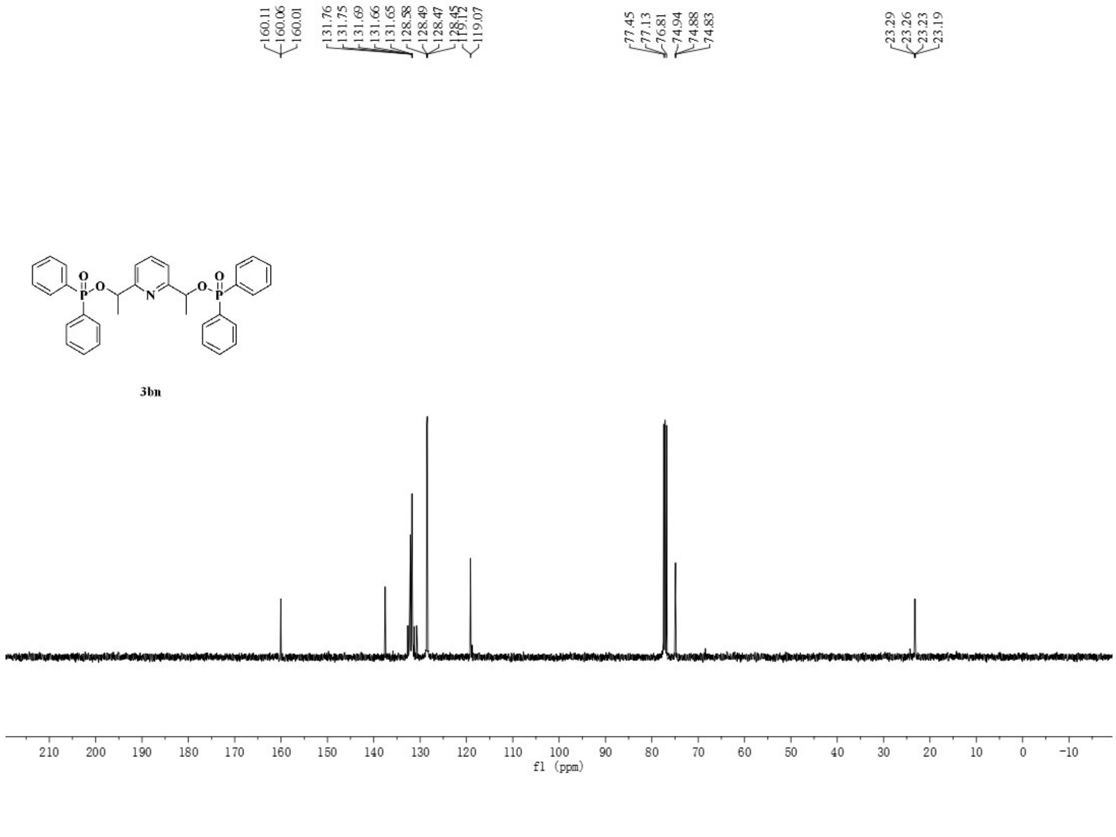

^{31}P NMR of **3bn** (121 MHz, $CDCl_3$)

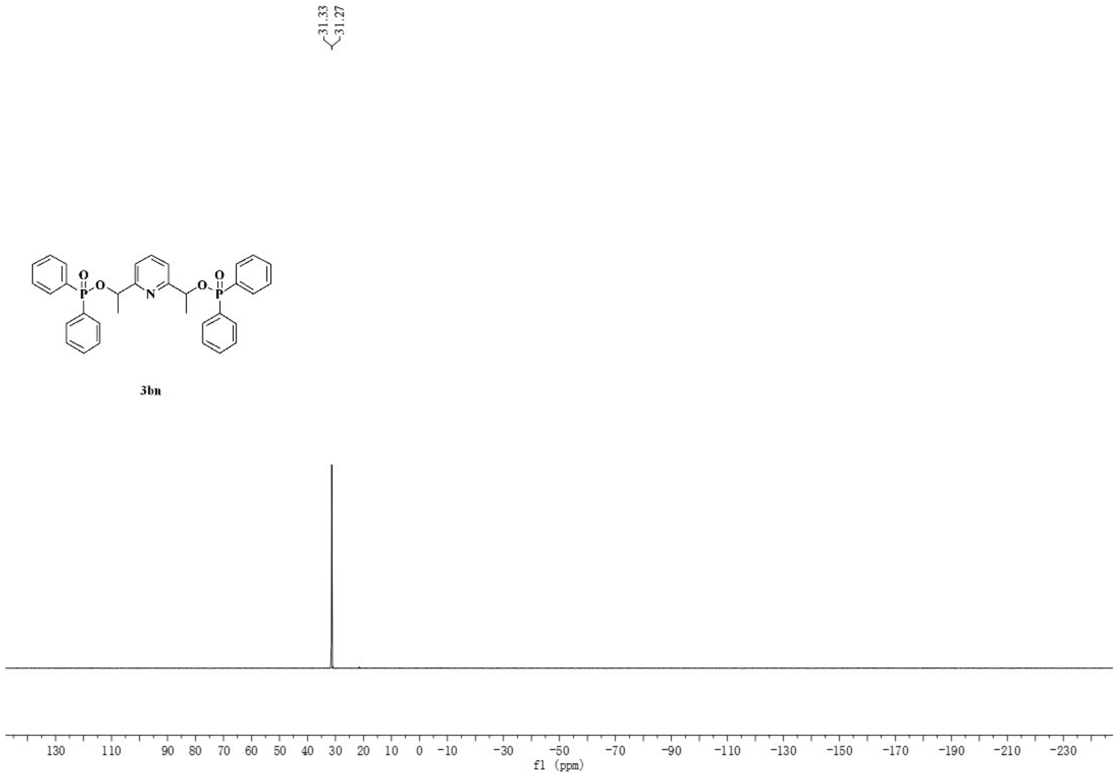

3bn

^{1}H NMR of **3bo** (400 MHz, $CDCl_3$)

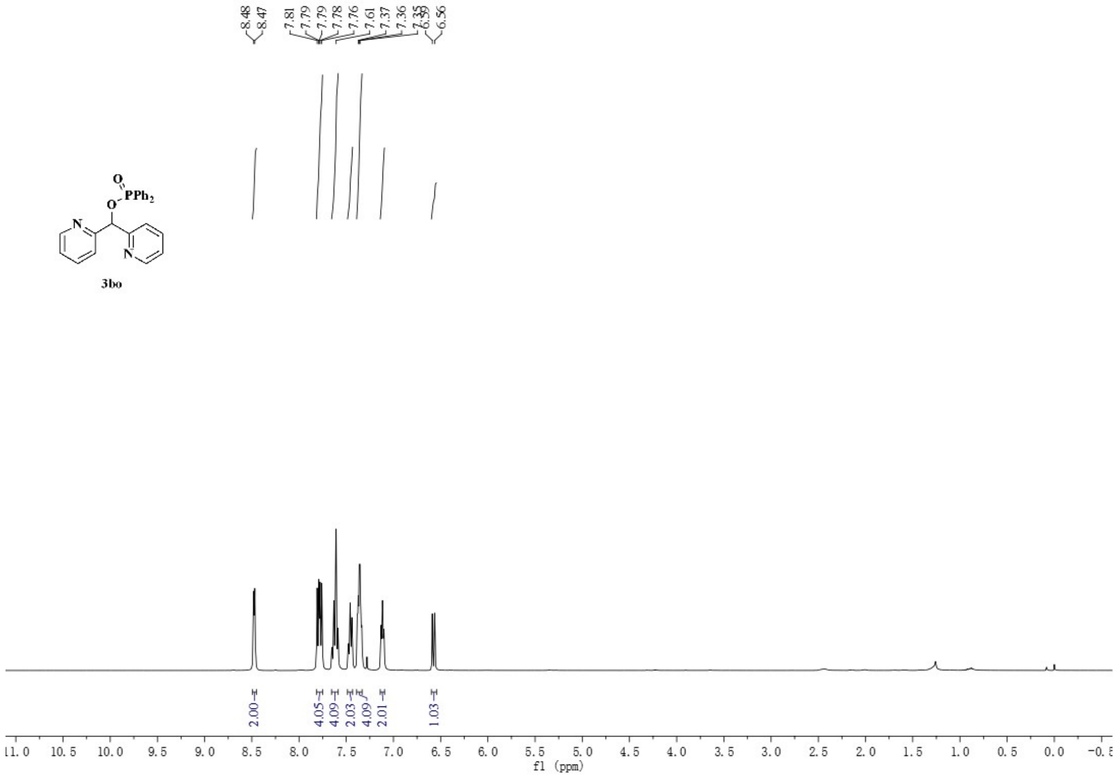

3bo

^{13}C NMR of **3bo** (101 MHz, $CDCl_3$)

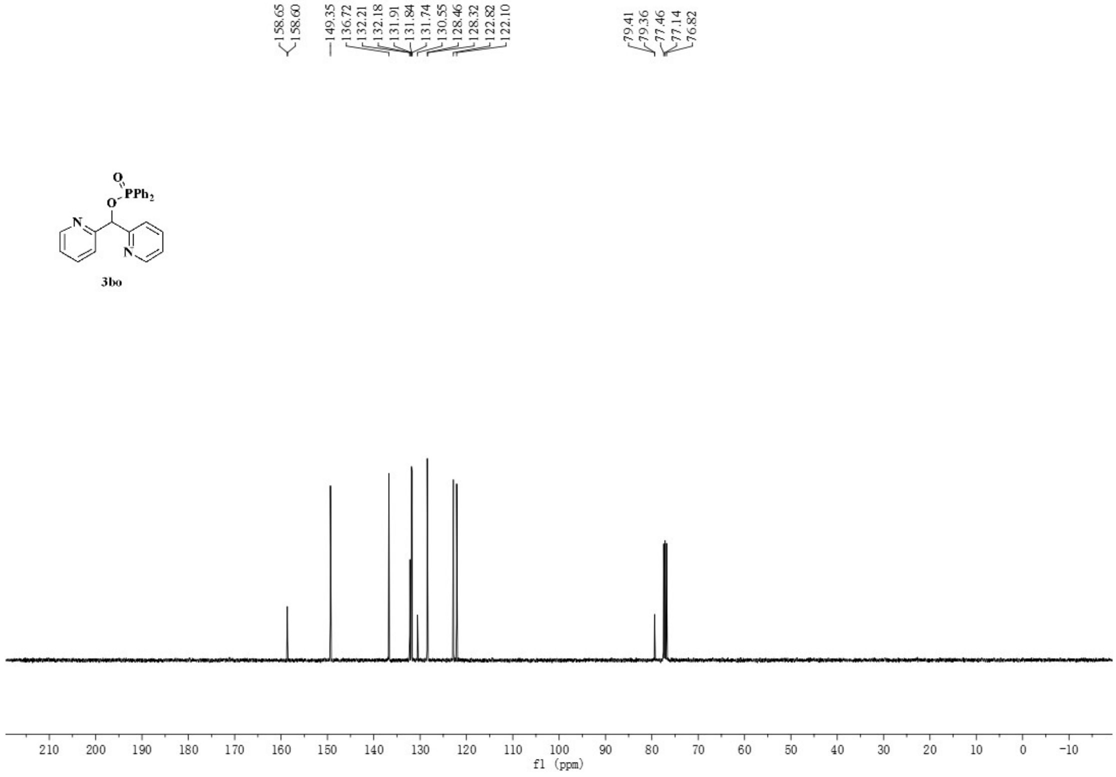

^{31}P NMR of **3bo** (121 MHz, $CDCl_3$)

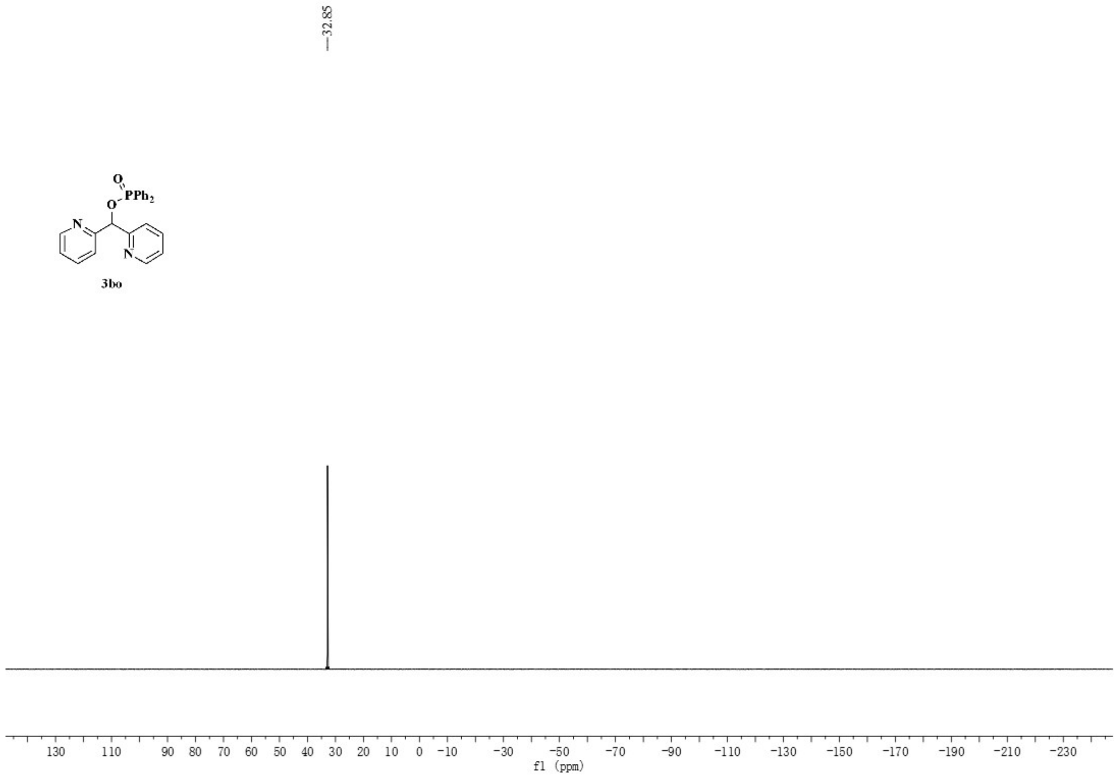

^{1}H NMR of **3bp** (400 MHz, $CDCl_3$)

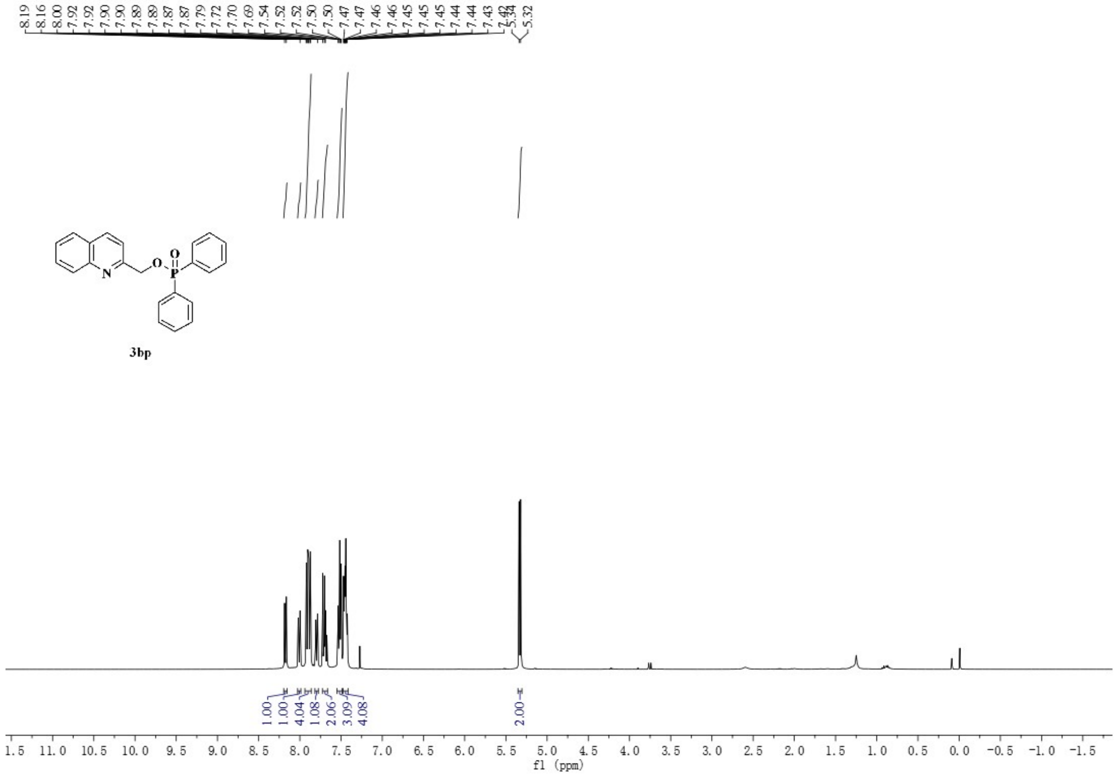

^{13}C NMR of **3bp** (101 MHz, $CDCl_3$)

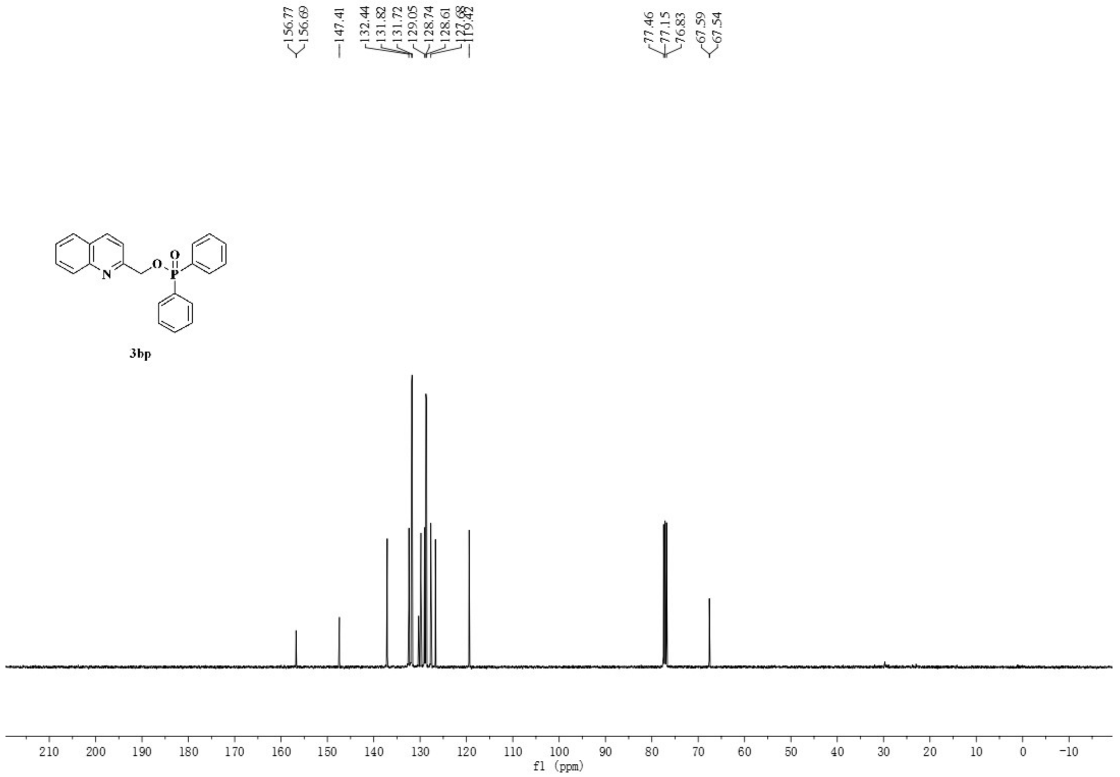

^{31}P NMR of **3bp** (121 MHz, $CDCl_3$)

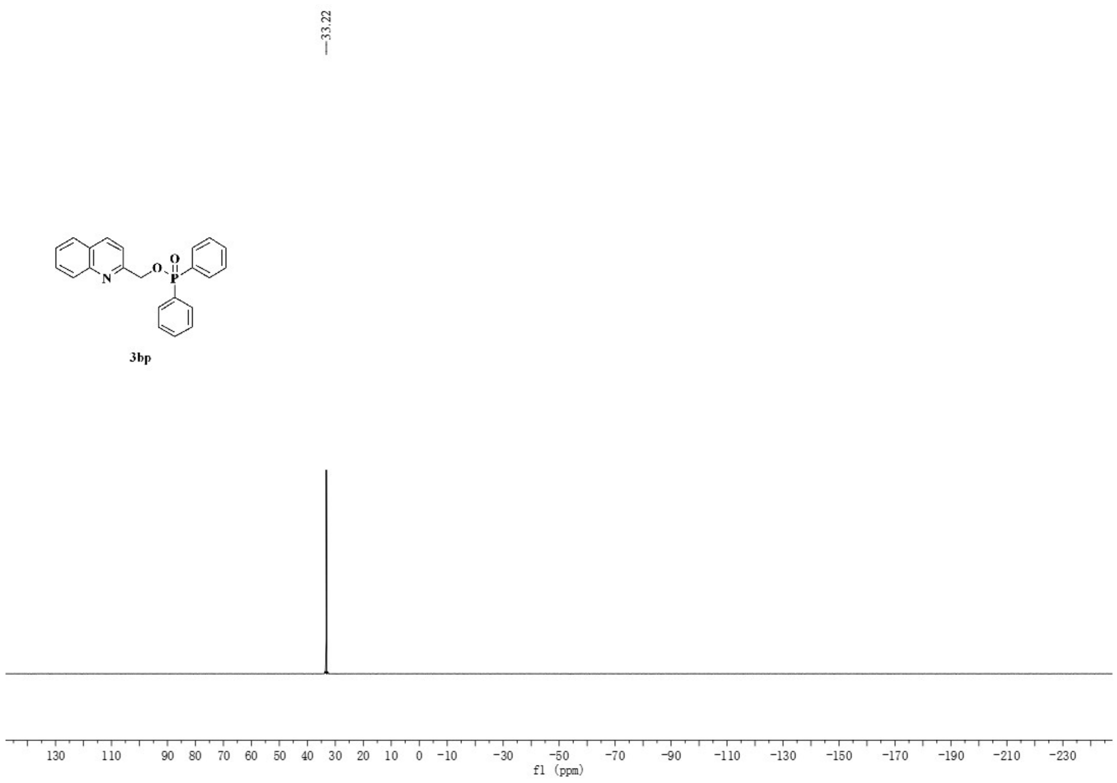

^{1}H NMR of **3bq** (400 MHz, $CDCl_3$)

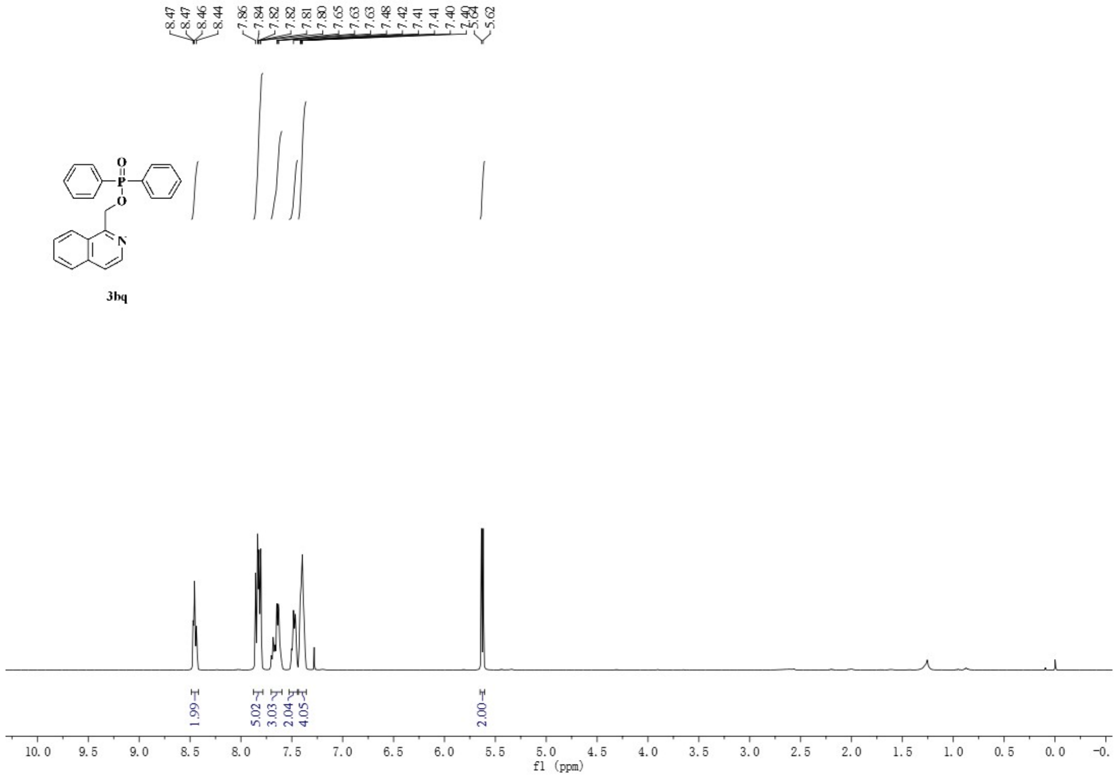

^{13}C NMR of **3bq** (101 MHz, $CDCl_3$)

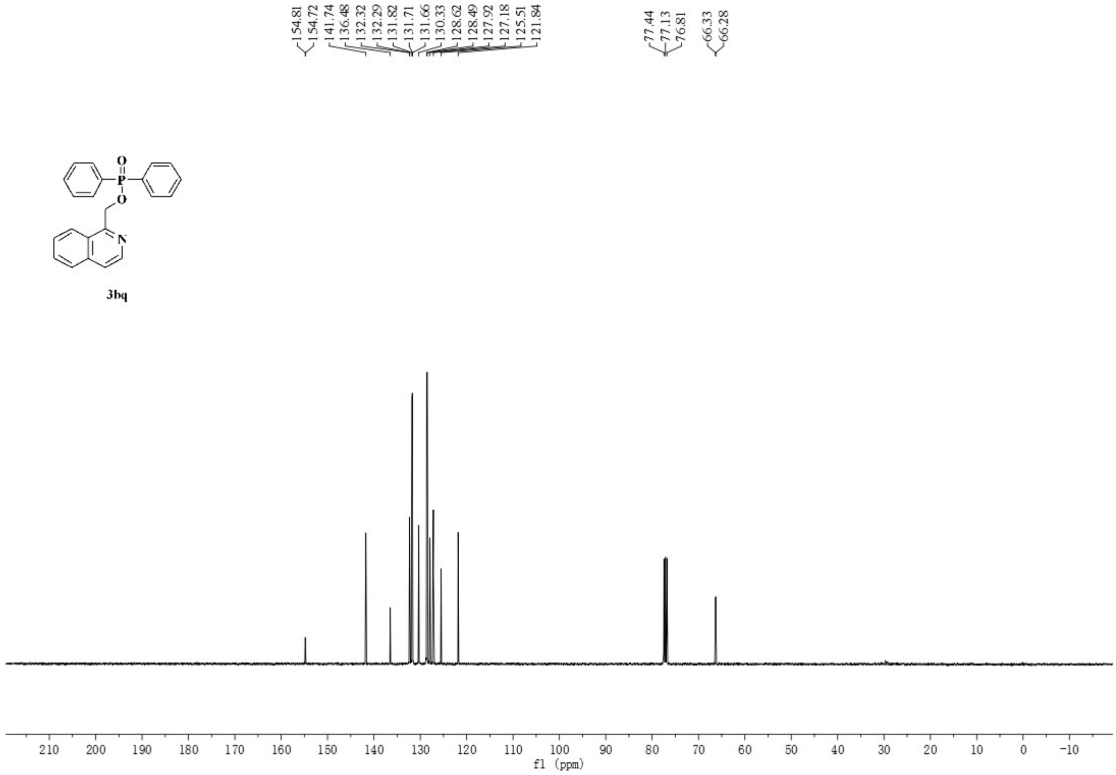

^{31}P NMR of **3bq** (121 MHz, $CDCl_3$)

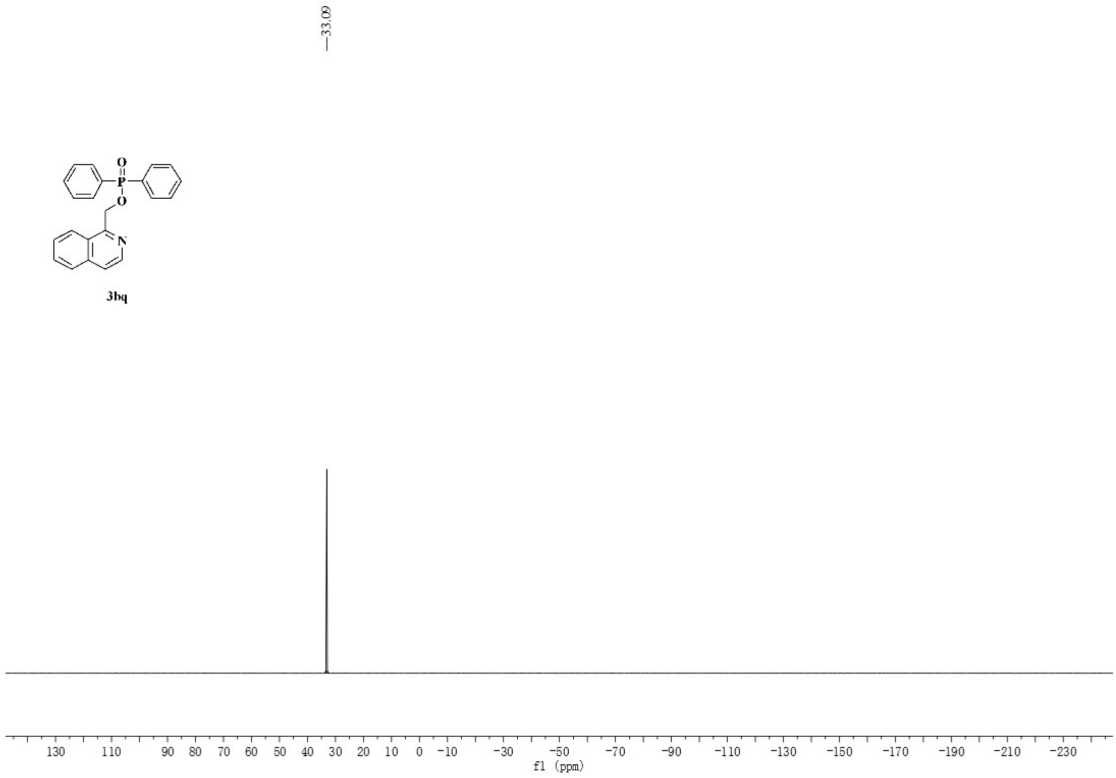

Supplement: File 1 — Experimental details and characterization data (1H, 13C, and 31P NMR as well as chromatograms) of products. [file Beilstein_J_Org_Chem-18-1188-s001.pdf]
